# A database of biological and geomorphological sea-level markers from the Last Glacial Maximum to present

**DOI:** 10.1038/sdata.2018.88

**Published:** 2018-05-29

**Authors:** F.D. Hibbert, F.H. Williams, S.J. Fallon, E.J. Rohling

**Affiliations:** 1 Research School of Earth Sciences, The Australian National University, Canberra, ACT 2601, Australia; 2 Ocean and Earth Science, University of Southampton, National Oceanography Centre, Southampton SO14 3ZH, UK

**Keywords:** Palaeoclimate, Climate change

## Abstract

The last deglacial was an interval of rapid climate and sea-level change, including the collapse of large continental ice sheets. This database collates carefully assessed sea-level data from peer-reviewed sources for the interval 0 to 25 thousand years ago (ka), from the Last Glacial Maximum to the present interglacial. In addition to facilitating site-specific reconstructions of past sea levels, the database provides a suite of data beyond the range of modern/instrumental variability that may help hone future sea-level projections. The database is global in scope, internally consistent, and contains U-series and radiocarbon dated indicators from both biological and geomorpohological archives. We focus on far-field data (i.e., away from the sites of the former continental ice sheets), but some key intermediate (i.e., from the Caribbean) data are also included. All primary fields (i.e., sample location, elevation, age and context) possess quantified uncertainties, which—in conjunction with available metadata—allows the reconstructed sea levels to be interpreted within both their uncertainties and geological context.

## Background and Summary

Curated and complete archiving (i.e., with full observational and geochemical metadata) of indicators of former sea levels from multiple archives (e.g., corals, salt marshes, shorelines) is essential not only to address questions related to past changes in sea level, but also to couch current and future changes within a wider geological context. There is no single repository used by the community to archive information, and data are often drawn from disparate publications and repositories, with different data quality standards for each sub-discipline. Here we bring together published sea-level data from a wide range of sub-disciplines that encompass both biological and geomorphological archives.

Consistent treatment of each of the individual records in the database, and incorporation of fully expressed uncertainties, allows datasets to be easily compared. We focus on the transition from the last glacial to the current interglacial period, which is relevant to our understanding of future extreme sea-level change because it provides a suite of data beyond the range of modern/instrumental variability with which to robustly test simulations^1–4^. Notably, the interval incorporates the last deglaciation, the most recent period of widespread destabilisation and collapse of major continental ice sheets. In model-based projections of future sea-level change^5^, the contribution of polar ice-sheet collapse is associated with large uncertainties, for example, regarding the rates and mechanisms of response to climate forcing. Past sea-level records provide some constraint on the natural bounds to the rates and magnitudes of polar ice-sheet decay^6–8^. Our overarching goal therefore is to establish an open-source, community-led archive that will accelerate research on the rates and magnitude of past sea-level change through the interval of time for which the most detailed information exists.

**Figure 1:**
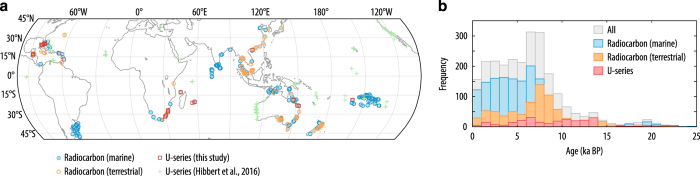
Location and age of fossil samples within the database. (**a**) Location of fossil samples: U-series dates (this study, red, open squares; Hibbert *et al.*^12^, green crosses); marine radiocarbon (blue, filled circles) and terrestrial radiocarbon samples (orange, open circles). (**b**) Age frequency of fossil samples: all samples in the database (Data Citation 1, grey); U-series (red); and radiocarbon dated (marine, blue; terrestrial, orange).

Spatially, the database is global in scale, with a focus on far-field sites (Fig. 1). We concentrate on far-field sites, because other compilations are available for near-field sites, mainly based on salt marsh samples^9–11^. We currently include microatoll data only where we are able to relate the elevation to a tidal datum, as we lack sufficient expertise to fully assess the physical and ecological relationship of this indicator to sea level. Temporally, the database concentrates on the interval 0 to 25 ka. At present, the database incorporates both U-series and radiocarbon dated samples. The database will be continually maintained and updated.

The compilation contains 194 studies (Table 1 (available online only)) from 40 locations (~2,600 data points) and includes all raw information and metadata, in contrast to other compilations where only a finalised age and relative sea level is given. This dataset complements and enhances the dataset of Hibbert *et al.*^12^, adding different types of sea-level indicators (e.g., mangroves, bivalves and gastropods) and incorporating both radiocarbon and U-series dating methods. The present compilation contains ~2,600 sea-level markers for the past 25 ka, compared to 630 in Hibbert *et al*.^12^.

Four broad types of information are required to reconstruct former relative sea levels^13–15^: (1) location (including tectonic setting); (2) sample elevation and uncertainty; (3) sample age and uncertainty; (4) sample information and context, which includes how the sample relates to sea level at the time of formation. The inclusion of all available data (i.e., published, with some clarification from authors, where necessary) and associated uncertainties in these four categories, for each dataset in the compilation, places the sea-level indicators within a well-defined wider environmental context. This aids interpretation and ensures the continued utility and value of each contributing dataset. In addition, as all available ‘raw’ age data are included for both U-series and radiocarbon dated samples (e.g., activity ratios, spike calibration, decay constants, corrections applied), users are able to recalculate ages for the samples, if desired, which ensures continued utility of the data into the future.

No correction has been made for glacio-isostatic (GIA) processes. Instead, we present relative sea level records with extensive documentation, and refrain from making any interpretations. However, when the database is applied, GIA considerations and corrections will become necessary.

## Methods

**Figure 2:**
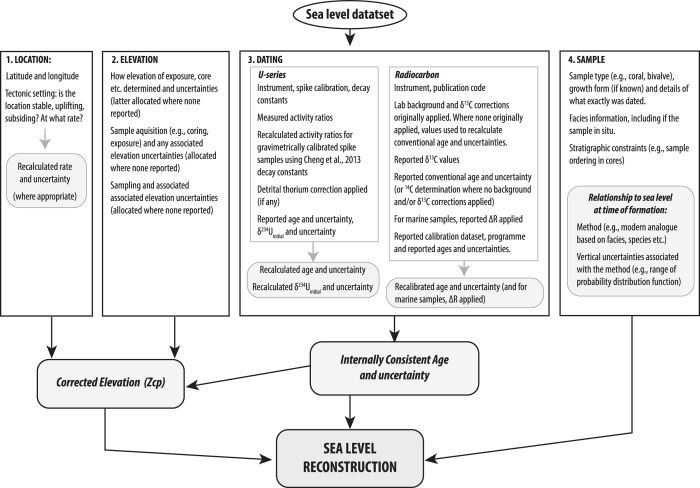
Simplified schema of the deglacial sea level database giving an overview of data acquisition and processing. The numbered boxes are the four essential components needed to reconstruct former sea levels: (1) location; (2) elevation; (3) age and; (4) sample information and other contextual information (including how the sample dated relates to sea level at the time of formation). Within each of these boxes we list the primary information recorded. Grey boxes indicate additional processing of data from original publications and new outputs (also included in the database, Data Citation 1).

All data have been obtained from peer-reviewed papers and books. Authors were contacted where information was missing or clarification was needed. Samples that still fail to reach a complete set of database fields have been excluded from our relative sea-level reconstructions. However, such samples are retained in the database because they may be important for other analyses. Figure 2 summarises the treatment of datasets within the database, and a brief outline of data acquisition and processing is given below.

### Location

Each data point in the database has been assigned a unique identifier, along with the original sample or analysis identifier. Sample locations are as originally reported. Where this information was lacking or insufficiently detailed, the latitude and longitude were estimated.

### Tectonic setting

The tectonic setting of a sample affects the reconstructed sea level through the interaction of uplift or subsidence with the measured elevation. Ideally, uplift/subsidence rates should be independently constrained. However, only Tahiti^16,17^ and Mururoa Atoll^18^ have such independent constraints. For most sites, the rates are often determined using the maximum elevation of the fossil coral terrace corresponding to the Last Interglacial, and an assumed age and relative sea-level position for the Last Interglacial. Occasionally, independent data (e.g., radiometrically dated lava flows) constrain the uplift/subsidence rate and we use these constraints where available (Mururoa Atoll^19^; Tahiti^17,20,21^). Where no independent constraints are available, we have recalculated the uplift rates from the elevation of the maximum Last Interglacial terrace and an assumed Last Interglacial age and sea level (Table 2 (available online only), as per Hibbert *et al.*^12^).

### Sample elevation and uncertainty

The elevation uncertainty of a sample falls into two broad categories: (i) the measurement uncertainty related to the method used for establishing the elevation of the outcrop or core and (ii) sampling uncertainties associated with both the method of sample acquisition (e.g., core stretching or shortening errors), which is dependent upon the method, and uncertainties that arise from sampling a core or section. Where information is missing in the original publication, we allocate a method-appropriate uncertainty. For example, where there is no mention of how the elevation was obtained or where only the method is given (e.g., levelling), we allocate a±0.5 m and±0.03 m (cf. ref. 22) uncertainty (2σ), respectively. Table 3 details the allocated uncertainties used in the database. The elevation uncertainty therefore is the root mean square of: (i) uncertainty associated with the method of establishing the elevation (e.g., levelling); (ii) uncertainties accounting for any distortion in obtaining the record (i.e., those resulting from coring methods) and; (iii) sampling uncertainties.

In order to compare elevations, a common datum is required. Within the database, we note the datum to which all measurements relate and, where possible, we reference all elevations to mean sea level (MSL) using appropriate tidal parameters (e.g., when converting elevations referenced to mean low water springs (MLWS) to MSL). We do not include any tidal errors; the modern tidal range often is not reported and variations in the past are poorly constrained.

### Sample age and uncertainty

The database incorporates samples dated using U-series and radiocarbon methods. Detailed descriptions of the systematics of both these techniques are available elsewhere (e.g., for U-series dating^23–25^; for radiocarbon dating^26–28^). A brief summary of data type and processing is given in the following.

#### U-series analyses

We record the instrument, method of spike calibration, decay constants, activity ratios, and detrital thorium correction used in the original age determination (also included). For samples where the spike was calibrated gravimetrically, we recalculate the activity ratios using the most recent decay constants^29^. For all samples, we then iteratively recalculate ages (equation 1) and δ^234^U_intial_ (equation 2) assuming a closed system and using the most recent decay constants^29^ (calculations were made using Isoplot v. 3.5 ref. 30). The reported uncertainties include the error associated with the decay constants.

(1)1−[T230hU238]act=e−λ230T−(δ234U(meas)1000)(λ230λ230−λ234)(1−e(λ234−λ230)T)

(2)δ234Uinitial=(δ234Umeasured)e(λ234T)

where, [^230^Th/^238^U]_*act*_ is the ^230^Th/^238^U activity ratio; λ_238_, λ_234_, λ_230_ are the decay constants of ^238^U, ^234^U and ^230^Th respectively^29,31^; δ^234^U_(meas)_ is the measured value of the activity ratio of ^234^U/^238^U relative to secular equilibrium in per mille (δ^234^U=([^234^U/^238^U] – 1) x 1000); and *T* is the age of the sample in years.

We make no attempt to account for any open-system behaviour (i.e., the remobilisation of nuclides) within the U-series dated datasets because the identification and correction of open system behaviour continues to be complex and debated (e.g., ref. 24). In addition, we do not screen the recalculated ages for reliability; there are multiple approaches to assess age reliability and the inclusion of all metadata and the original reported ratios etc., allows users to determine appropriate age-reliability screening criteria (e.g., the bounds of acceptable δ^234^U_initial_ values, % calcite etc.).

Ages are reported as ka BP in order to ensure that they are comparable to the radiocarbon ages, which are by convention reported as years before 1950 AD. We adjust the age for the time elapsed since analysis. Where no date of analysis is given, we have assumed this was the year of publication. We recognise that this may introduce additional age uncertainty but anticipate that this only a few years and typically less than a decade.

#### Radiocarbon analyses

We record the laboratory, instrument, publication code, any corrections applied by the laboratory (i.e., background and δ^13^C corrections) and both the conventional and calibrated ages and associated uncertainties for each sample (including any regional marine reservoir age correction, ΔR, applied by the authors). We also report the δ^13^C values for samples, and the calibration dataset and programme where provided. Where no background and/or δ^13^C correction was applied by the laboratory, we apply a sample-specific normalisation (terrestrially derived organic material δ^13^C=−25±2 ‰; marine carbonates δ^13^C=0±2 ‰). The conventional age can then be calculated using the appropriate (instrument dependent) ^14^C/^12^C or ^14^C/^13^C equations^32^. Age uncertainty is reported at the 1σ level in accordance with standard radiocarbon reporting protocols^33–35^.

We assume that sample materials obtained their carbon from only one reservoir (i.e., atmospheric or marine). Additionally, we assume that estuarine bivalve and mollusc samples are fully marine because additional information, such as δ^18^O and δ^13^C analyses, that would help establish the environment in which the sample was living is often not available. We recognise that there may be considerable variation in the regional marine reservoir correction (ΔR) for estuarine bivalve and mollusc samples due to the varying mixing of marine and freshwater^36–39^ which potentially results in an older apparent age for specimens living in estuarine environments.

A radiocarbon measurement requires an additional step of calibration to obtain an age estimate due to the non-linear nature of the ^14^C timescale^40^. Both the calibration procedure itself (given the complexity of the calibration dataset) and the choice of software and parameters (such as the use of Bayesian statistics to construct age-depth models)^41,42^ influence the final calibrated age of a sample.

The calibration curve may affect the statistical inference of time because the relationship between the radiocarbon age and the calendar age changes through time, due to variations in the radiocarbon concentration (e.g., refs 43,44). In addition, the shape of the calibration curve (non-monotonic with inversions) means that calibration is non-commutative and directional^45^, with distortions due to the structure of the curve itself^43^, the potential for the production of artificial peaks^46^, and the amplification of the output probability density function by steep sections of the calibration curve^45,47^. This can result in the summed probability density function of a calibrated date exceeding the ‘true’ time interval of the event^48^.

Different calibration algorithms may affect the final calibrated age probability distribution, particularly when comparing results from software packages that do, or do not apply Bayesian statistics, i.e., where the age-depth model uses different depositional models to mimic sediment deposition processes. For example, OxCal^49^, BChron^50^, and Bacon^51^ utilise Bayesian statistics to incorporate stratigraphic and other chronological information to formulate prior distributions for the calibrated dates, and to provide ‘best-estimate’ age-depth models with uncertainties. In the database, we have chosen not to implement such age-depth modelling routines for datasets with stratigraphic ordering when recalibrating the radiocarbon dates, for several reasons: (1) to ensure consistency within the database; (2) because not all samples in the database have simple stratigraphic relationships, for example, coral reefs are complex 3-dimensional structures that do not necessarily accumulate monotonically like sediment cores, and; (3) to refrain from imposing any structure on future analysis. Overviews and comparisons of the main age-depth modelling routines are available^41,42^, should users wish to apply these on appropriate, individual subsets of the database. Samples with stratigraphic ordering are clearly identified in the database with a numeric identifier for each group, and ordering given by subdivision of that number, smallest/topmost to largest/lower-most sample.

The conventional radiocarbon age and uncertainty for each sample were recalibrated using OxCal version 4.3. (ref. 52) and the latest calibration datasets: IntCal13 (ref. 53) for northern hemisphere terrestrial samples; SHCal13 (ref. 54) for southern hemisphere terrestrial samples; and Marine13^53^ for all marine samples. For marine samples, we apply a local marine reservoir correction (ΔR^55^) to account for regional variations in the offset between the marine and terrestrial carbon reservoirs (the marine reservoir effect). The marine reservoir effect (i.e., the offset in the radiocarbon age of marine materials compared to materials deriving their ^14^C from the atmospheric at the same time) is spatially and temporally variable. The spatial variation from a calculated global average is accounted for by using a regional offset (ΔR). A consistent value of ΔR was applied for each coherent geographical region (i.e., for all sites influenced by the same surface oceanographic circulation) and estimated from the online database^56^, double checked with previous ΔR determinations (Table 4 (available online only)). The online database^56^ of values (and calculations of ΔR^57^) is used to ensure both the correct and consistent calculation of ΔR. Note that the method used to calculate ΔR in the online database incorporates the full probability distribution unlike ‘classical’ intercept methods, so that the resulting ΔR uncertainties are more accurate (full discussion of the methodology^57^). Where more than one ΔR value is used, we calculated an error weighted mean and uncertainty. We apply the pre-industrial calculated ΔR, but recognise that ΔR is also temporally variable^58–60^. Applying a pre-industrial ΔR does not account for any variations through time as a result of changing climatic and surface-ocean conditions, or variations in the production of ^14^C in the atmosphere with variations in the Earth’s magnetic field e.g., ref. 61. In general, there are few locations in the database and a limited number of studies where the temporal variability in ΔR has been investigated. As this variability is largely unconstrained at present, we do not attempt to account for this uncertainty in the database but the effect would be most pronounced for sites with data spanning the transition from the glacial to interglacial, when reorganisations of ocean circulation and of carbon stores within the ocean may have led to potentially large variations in ΔR. It should be noted that any such age uncertainty may additionally affect the resulting P_RSL_ reconstruction of some sites through interaction with uplift or subsidence rates.

The output of a calibrated radiocarbon date is a probability density function. The calculated posterior probability distributions are often multimodal and difficult to summarise, except via graphical representations^41^. Reporting of the 68 and 95% confidence interval has become common, although not universal, in part due to the ease of plotting a point estimate. Point estimates (such as the mean, mode, median etc.) do not fully account for the variation in the output of calibration (i.e., the resulting multimodal distributions), and none of these point-based estimates can be considered a good estimate of the full complexity of the calibrated date^44,62^. It is difficult within a database to accurately record the outcome of calibration. However, because all information required for calibration of a date is included in the database (*inter alia*: conventional radiocarbon date and uncertainty; material dated; ΔR for marine samples; calibration curve, programme and version), users can recalibrate the data and obtain the same probability density function as captured by the 68 and 95% confidence intervals listed in the database. The complete documentation also allows recalibration of the dates following future refinements of the calibration datasets, etc.

In our recalculation (where appropriate) and recalibration of radiocarbon samples, we take care to ensure that we round the calibrated age (to nearest whole number) only at the end of the process. However, we are unable to guarantee that is the case of the reported values used in each of the processing steps.

### Sample information and context

Detailed information on both the sample and its geological context is vital. We record available information from the publications including: what material was dated (and species, if given); the facies context and/or other outcrop and unit information; whether the authors determined the sample to be in growth position and/or *in situ*; and the growth form (e.g., branching or massive corals, if given).

In addition, to reconstruct past sea levels, we must establish the relationship between the sample and sea level at the time of its formation (i.e., the ‘indicative meaning’ which describes the range of elevations, with respect to a specified tidal datum, that a particular indicator forms^13,14,63^). This is often achieved using a modern analogue, i.e., looking at the modern elevation range of a sea-level indicator in relation to present sea level (or some tidal datum). This approach is subject to key assumptions: (i) that the modern depth distribution is the most appropriate analogue; (ii) that the relationship is stable through time and; (iii) that the fossil record is a faithful approximation of the living diversity and distribution (i.e., minimal loss of detail due to taphonomic processes).

We use two different approaches for representing these relationships. The first uses a specific probability distribution for each taxon (e.g., the modern depth distribution of a coral species; following the methodology of Hibbert *et al.*^12^), and the second assumes a uniform probability distribution because the sea-level indicator forms somewhere within an altitudinal range but we have no further information as to the most likely depth or elevation (e.g., an oyster living somewhere within the intertidal to low-supratidal range at a given site).

#### Using a specific probability distribution of a species

For coral sea-level indicators, we are able to define a probability distribution for the depth-habitat (using the methodology detailed in Hibbert *et al.*^12^ and summarised here). In this iteration of our analysis, we update the datasets used to define each taxon-specific depth distribution using the latest release from the Ocean Biogeographical Information System (www.iobis.org). The data in the OBIS dataset have been rigorously quality controlled. We use only observational and live-collected data with a vertical precision of ≤0.25 m. In some instances, there are insufficient observations (<150) to constrain the depth distributions and so the depth precision criterion was relaxed: *Alveopora* sp. has a depth precision of ≤0.5 m (*n*=171); *Favia fragum* and *Porites solida* have a depth precision of ≤ 2 m (*n*=183 and 149 respectively) and; *Acropora abrontanoides* has a depth precision of ≤ 5 m (*n*=132). For some fossil species used to reconstruct past sea levels (*Goniopora lobata* and *Gardinerosis planulata*), little or no modern observational data were available and, in these instances, we use the modern genus depth distributions. We urge caution where fewer than 150 observations constrain the depth distributions.

**Figure 3:**
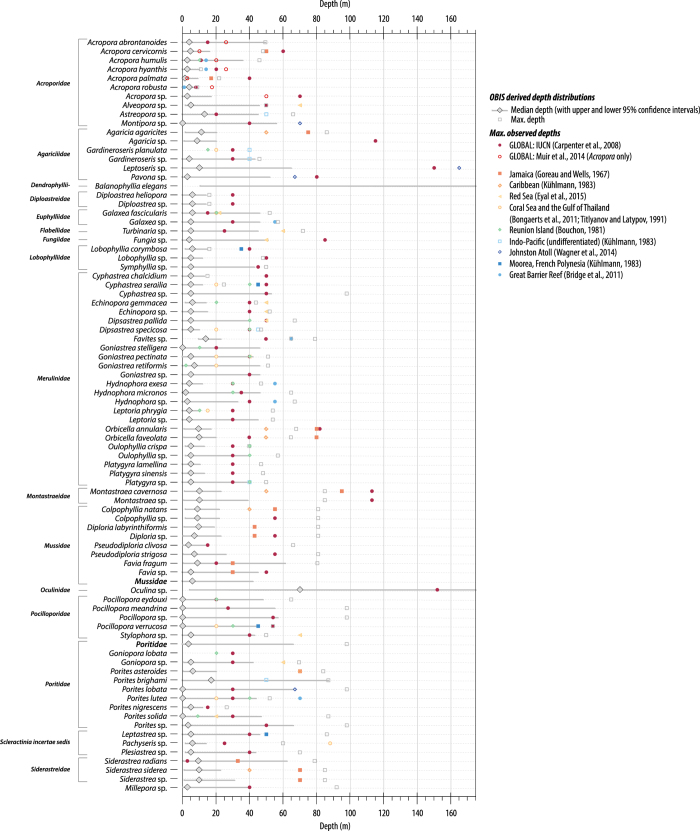
Coral depth distributions. Median (grey, filled diamond) and 95% confidence intervals (grey horizontal bars) for the ecologically derived depth distributions. The ICUN^89^ global estimates of maximum depth (dark red, filled circles) and those derived for *Acropora* sp.^90^ (red, open circles) are given for comparison. Also plotted are the maximum depths for certain recorded at various locations: Jamaica^75^ (orange, filled squares); the Caribbean^92^ (orange, open diamonds); The Red Sea^97^ (yellow, filled triangles); the Coral Sea^94^ and Gulf of Thailand^93^ (yellow, filled circles); Reunion Island^91^ (green, filled diamonds); the Indo-Pacific^96^ (blue, open squares); Johnston Atoll^92^ (dark blue, open diamonds); Moorea, French Polynesia^92^ (dark blue, filled squares) and; the Great Barrier Reef^95^ (blue, filled circles).

For each taxon, we derive an estimate of the median water depth in which the modern species lives (Fig. 3). We have chosen the median rather than the mean because the depth distributions are not Gaussian or symmetrical and because the mean is more sensitive to outliers. The lower and upper bounds of the 95 and 68% confidence intervals were also determined using the 2.5, 97.5, 16 and 84 percentiles, respectively (Table 5 (available online only); all depth observations used can be found in Data Citation 2 so that users may ‘draw’ directly from the distribution, if desired). We compile depth distributions at a ‘global’ scale (i.e., using all information available for the species) as well as geographical subsets: ocean basin, sub-basin and, where sufficient information is available, regional subsets (for example, Atlantic, Caribbean, Belize or Pacific, SW Pacific, Great Barrier Reef). These regional distributions are included as a first-order approximation of the modern variability (both geographically and with depth) of coral taxon distribution^64,65^. Our ecological depth distributions are especially useful for sites lacking site-specific assemblage work that would constrain the modern relationship between coral depth and sea level.

In general, there are very few observations in the Indian Ocean and so it was not possible to further constrain the depth distributions for this region.

**Figure 4:**
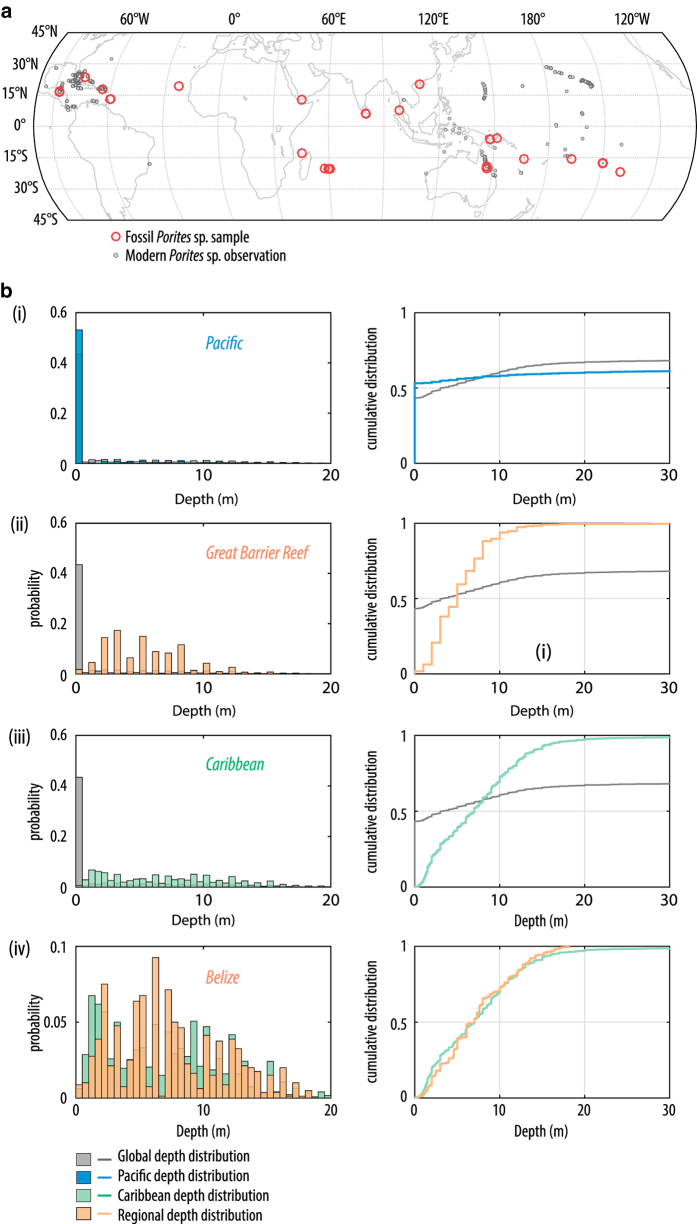
Modern observations of *Porites* sp. used to constrain depth distributions. (**a**) Map of fossil (red, open circles) and modern *Porites* sp. observations (plotted only those observations used to derived the depth distributions) (grey, filled circles). (**b**) Global (grey), basin and sub-basin (pink) depth distributions for *Porites* sp. represented as relative probability (normalised histograms, left panels) and cumulative frequency distributions (right panels): (i) ‘Global’ (grey) and Pacific (blue); (ii) ‘Global’ (grey) and Great Barrier Reef (orange); (iii) ‘Global’ (grey) and Caribbean (green); (iv) Caribbean (green) and Belize (orange).

In the Pacific, there are significant numbers of observations but once sub-divided into sub-basin and regional locations, only the Great Barrier Reef (GBR) has sufficient, systematic observations (i.e., regular recording of data to depths of ~10 m and greater) to allow determination of robust regional depth distributions. For the most of the Pacific region, despite large numbers of observations, there appears to be a shallow-water bias, with observations concentrated within the upper couple of meters (for example using *Porites* sp., Fig. 4). Additionally, there are too few observations to allow determination of regional depth distributions with any confidence, particularly for the east and southeast of the basin. The depth distributions determined for the GBR region are based on numerous observations and span a greater depth range than other Pacific observations. However, collating observations from such a large geographical area likely masks the modern complexity of coral distribution within the reef system e.g., refs 66–68. Nonetheless it represents a first step in refining sea-level reconstructions, by incorporating a first-order approximation of the geographic variation in coral diversity and distribution. It should be noted that at present there are relatively few fossil corals in the database from the Great Barrier Reef (GBR) itself (*n*=27 but, of these, 15 have been determined only to the genus level). The similarity between reef ecology, distributions and growth forms between Vanuatu and the GBR^69^ also allows us to use the GBR depth distributions to refine sea-level reconstructions for Vanuatu. This is especially useful given that most (~70%) fossil corals from Vanuatu do not have original water depth determinations from modern biozonation of corals, coralline algae etc.

**Figure 5:**
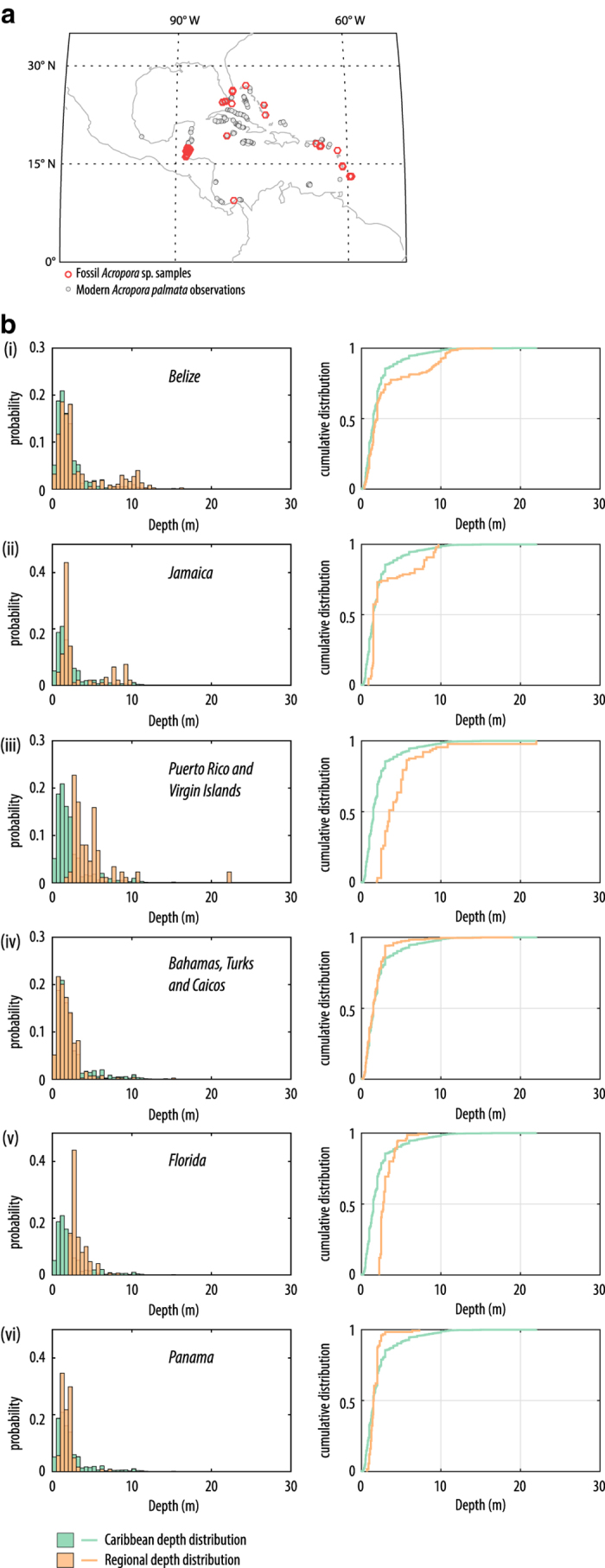
An example of regional depth distributions for *Acropora palmata* from the Caribbean sub-basin. (**a**) map of the fossil *Acropora* sp. samples (red open circles) and *A. palmata* observations used to constrain the depth distributions (grey, filled circles); (**b**) Caribbean depth distributions for *Acropora palmata* (green) and regional subsets (orange) represented as relative probability (normalised histograms, left panels) and cumulative frequency distributions (right panels).

In the Atlantic, there is a substantial number of observations, including for the Caribbean sub-basin and for many regional sites. This allows definition of several taxon-specific, regional depth distributions. Most of these regional depth distributions are constrained by at least 100 observations, with most regions having > 300 observations (see summary statistics in Table 6 (available online only)). For many regions within the Caribbean sub-basin, there are distinct differences in species depth preference (e.g., *Acropora palmata*, Fig. 5), with notable offsets to deeper or shallower habitats evident relative to the ‘global’ depth distributions. This likely represents spatial variations in the depth habitat of the species (given the site-specific factors governing coral distributions and diversity; see review of Hibbert *et al.*^12^) but may also be an artefact of sampling bias (i.e., shallow-water bias in sampling). For some Caribbean fossil samples (e.g., those from St Croix in the US Virgin Islands, Belize, and Panama), modern constraints on the relationship between (tectonically corrected) coral elevation and sea level at the time of formation (i.e., a palaeo-water depth relationship) are lacking. As such, the regional depth distributions generated here allow us to both reconstruct sea level, and to incorporate the modern complexity in the geographic variation in taxon depth preference. Without this information on the relationship between the sample and sea level at the time of its formation, only a (tectonically) corrected elevation could be calculated, not sea level.

It should be noted that both the ‘global’ and regional depth distributions are a ‘maximum’ representation of the vertical uncertainties associated taxon-specific depth distributions. Additional biological (e.g., associated species with a narrower depth range) or geomorphological (e.g., designation as reef crest facies) information might be used to reduce the total vertical range associated with the reconstructed sea levels, if such additional data were provided. Unfortunately, most samples currently lack such information.

The use of modern analogues (including our OBIS-derived depth distributions) to define the palaeo-water depth relationship has three primary caveats. First, for some sites the present may not be the most appropriate analogue due to human influences^70,71^. For example, the modern coral fauna of Barbados is not representative of the Pleistocene reefs due to reef destruction and loss of coral species, particularly the mass mortality of once dense populations of *Acropora palmata*^72,73^. Fortunately, given the number of fossil corals from Barbados in the database, the similarity between the recurrent patterns in species dominance and diversity observed between the raised reef terraces of Barbados and the living reefs of Jamaica^74,75^, first recognised by Mesolella^76^, justifies the use of modern regional depth distributions of Jamaica as an analogue for Barbados. Second, the fossil record may not faithfully capture the living reef assemblage and structure due to the potential for non-preservation and selective removal/alteration of material by physical, chemical and/or biological processes (i.e., taphonomic processes^77–81^). Third, a key assumption is the constancy and stability of the palaeo-water-depth relationship through time and, although difficult to determine, there is some evidence from the Caribbean that the large stands of branching *A. palmata* that dominated for the last 0.5 Ma are the same as those documented in the Caribbean until the early 1980’s, when human-induced habitat changes forced major changes in community structure^72,73^.

#### Using a facies formation range or biological indicative range

For the non-coral subset of samples, we use the depth range or facies formation depth range as determined by the original authors. Where this information is missing, we are unable to reconstruct past relative sea level. We assume a uniform distribution for the relationship, in that the indicator may occur equally anywhere within the given altitudinal range. Note, the original coral palaeo-water depth determinations would also have a uniform distribution, and could be treated in the same manner, if desired.

#### Limiting data

For some samples, we are only able to say confidently that sea level was above or below the (tectonically corrected) elevation of the sample at the time of its formation. For example, a fossilised tree provides an upper limit on sea level at the time of growth, in that sea level must have been lower than the elevation of the sample. This subset of data is included, although we are unable to confidently reconstruct relative past sea levels, as such data can be very useful for constraining models of glacio-isostatic processes.

### (Tectonically) Corrected position (*Z*_*cp*_)

Where appropriate, the modern elevation of the sample is corrected for uplift or subsidence since the time of formation, ensuring consistency between sites. For each sample, we are able to calculate the (tectonically) corrected position^12^ (*Z*_*cp*_) (equation 3)

(3)Zcp=Esam−(ΔHΔt*tsam)

where, *Z*_*cp*_ is the tectonically corrected elevation in m, and negative values are below sea level, *E*_*sam*_ is the elevation of the sea-level indicator referenced to mean sea level (MSL), ΔH/Δt is the recalculated uplift or subsidence rate in m/ka, with increasing positive ages in kilo-years before present and; *t*_*sam*_ is the recalculated (and recalibrated in the case of radiocarbon analyses) age of the sample in ka, with increasing positive ages in kilo-years before present (ka BP).

### Reconstructed Probability of Sea Level (P_RSL_)

**Figure 6:**
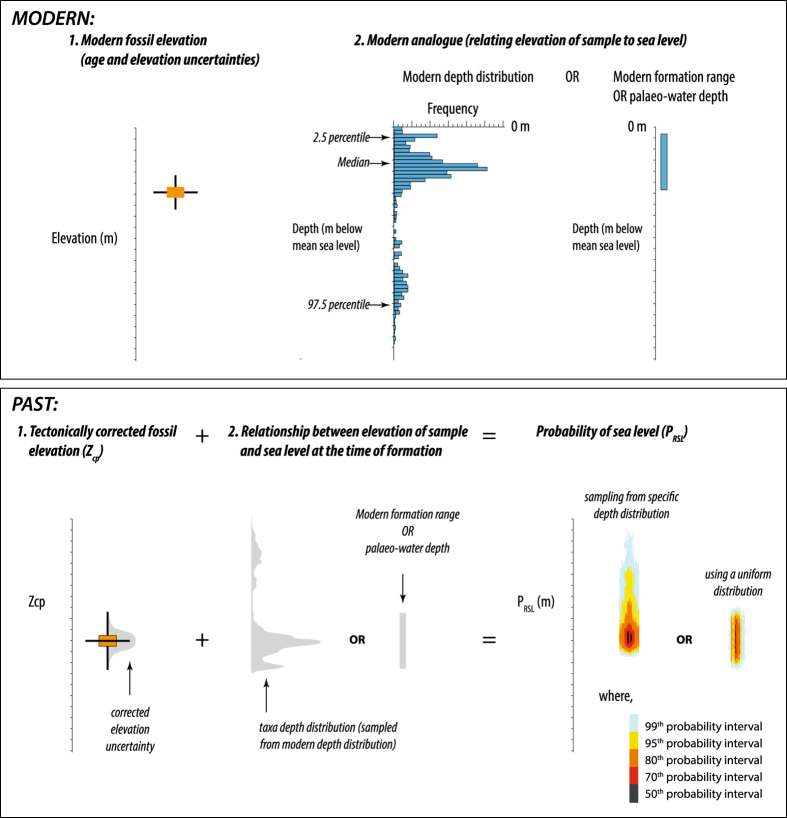
Schematic of relationship between, and uncertainty propagation for, the corrected coral position (Z_cp_) and the probability of sea level (P_RSL_).

We combine elevation uncertainties (including any uplift/subsidence correction) with the information relating the indicator to sea level at the time of formation (i.e., the modern altitudinal distribution for that indicator in relation to mean sea level) using the methodology of Hibbert *et al.*^12^. A schematic of this procedure is given in Fig. 6. We use a Monte-Carlo approach of 350,000 simulations to derive a probability maximum (P_RSL_) associated with each sea-level indicator position (*Z*_*cp*_) and a confidence interval around that point. For each sea-level indicator, we obtain a set of randomly sampled values from the corrected position (*Z*_*cp*_) uncertainty, and a set of randomly sampled values from the depth distribution (arising from either the empirically derived depth distributions for coral samples or a uniform distribution within a given formation range) and sum across the two errors. For each individual sea-level indicator, we then have multiple instances across a combined error distribution. From this set we can generate the probability distribution, and extract a probability maximum and the associated 1, 2- and 3- sigma equivalent levels (68%, 95%, and 99% probability intervals) (the code used is provided, Data Citation 3). Note, these are typically asymmetrical for fossil coral samples when our modern, taxon-specific depth distributions are used to calculate P_RSL_. Users of the database (Data Citation 1) are free to choose the relationship they deem most appropriate as we include the palaeo-water depth determined by the original authors, our OBIS-derived depth distributions (Data Citation 2), and the code (Data Citation 3) used to calculate P_RSL_.

The result is a probability distribution of relative sea level (P_RSL_) that incorporates both a eustatic, an isostatic and other (e.g., hydro-isostacy, compaction etc.) components. Note, we do not account for any glacio-isostatic processes as this is outside the scope of the present study. Additionally, we do not include any tidal corrections to our reconstructed sea levels to account for past variability in the magnitude and spatial variation of past tidal regimes. In many publications, the modern tidal range is not reported and variations in the past are poorly constrained at present.

## Data Records

The database (Data Citation 1) is designed to include all available data, for example we include all information relating to dating to enable users to recalculate the age, and associated metadata. ‘Data descriptors’ details all fields used in the database and can be found in Table 7 (available online only). The modern taxon-specific depth distributions (Data Citation 2) and the code (Data Citation 3) used to reconstruct past sea levels from fossil samples are also available from Figshare. A summary of the treatment of each the dataset in the database (Data Citation 1) can be found in ‘Supplementary Data’.

## Technical Validation

In addition to ensuring consistency of data processing and any recalculations (age recalculation, recalibration etc.), we have attempted to validate various data-processing steps, where appropriate, and details for this are given below.

### Age

Reported ages from the original publications are included in the database in addition to our recalculated ages (and recalibrated ages in the case of radiocarbon). This provides a first check of our age recalculations/recalibration. Note, that any uncertainty in the age determinations may propagate into our reconstructions of past relative sea-level through the interaction with uplift/subsidence.

#### U-series

All geochemical data are included in the database to enable users to recalculate the ages, if so desired. It should be noted that we do not screen the U-series ages for reliability. Users may select their own screening criteria (limits on acceptable δ^234^U_initial_ values, calcite content etc.) from the fields included in the database (for examples, see ref. 12).

#### Radiocarbon

Regional deviations from the global offset between the atmosphere and the surface mixed layer (i.e., the marine reservoir effect) are dealt with using an offset (ΔR) during calibration, with ΔR often assumed to be constant through time. The resulting final calibrated probability distribution of the sample therefore includes the uncertainty in the construction of the marine calibration curve (currently Marine13^53^), but not the uncertainty in the variation in ΔR through time^57^. The effect on resulting calibrated age of: (i) spatially and temporally variation of the regional marine reservoir correction (ΔR) and; (ii) the effect of assuming a uniform, rather the Gaussian distribution for ΔR is explored further here. The examples provided are for illustrative purposes only.

**Figure 7:**
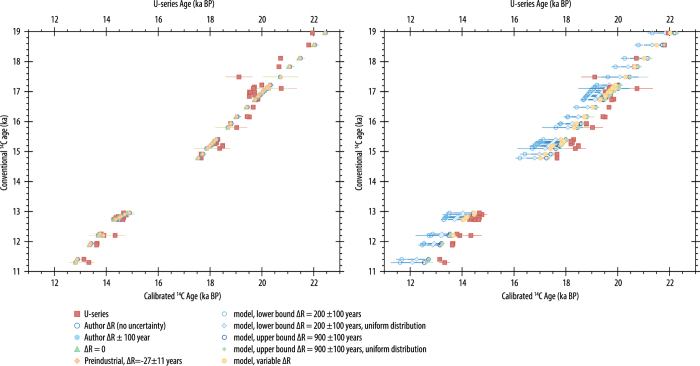
Investigation of the effect of variable ΔR on the calibrated age. This example uses corals from Barbados^82,83^ with both U-series and radiocarbon dates. U-series ages (red, filled squares) are recalculated assuming a closed system and the decay constants of Cheng *et al.*^29^. Radiocarbon data are recalibrated using ΔR values: (left panel) of the original authors (dark blue, open circles); the original authors and a±100 year uncertainty (blue, filled circles); ΔR=0 (green, filled triangles) and preindustrial value of ΔR=−27±11 years derived from Reimer and Reimer (ref. 56) (orange, filled diamonds). In the right panel, the U-series ages are compared to the recalibrated radiocarbon ages using ΔR values from the model of Butzin *et al.*^84^: ΔR=200±100 years using a Gaussian (blue, open circles) and uniform (blue, filled diamonds) distribution; ΔR=900±100 years using a Gaussian (dark blue, open circles) and uniform (green, filled diamonds) distribution; a variable ΔR (yellow, filled circles).

In order to investigate the possible magnitude of this effect—i.e., potentially disparate modern and glacial values of ΔR for the same region—we explore the effect of using different values for ΔR, different error distributions for ΔR (Gaussian and uniform distributions) for a marine dataset that possesses both radiocarbon and U-series age determinations (corals from Barbados^82,83^). The calibrated ages (calibrated using the OxCal calibration software, version 4.3 (ref. 52).) are compared to the U-series dates for the same samples (recalculated assuming a closed system and the decay constants of ^29^) (Fig. 7). Note, this exercise is an example only; for sea-level reconstructions, we would use U-series ages in preference to radiocarbon ages for these samples, in order to negate both calibration issues and the unconstrained variable ΔR. Additionally, for this example, we assume that the U-series ages for the samples are reliable, i.e., that there has been no addition or loss of isotopes from the system (i.e., no open system behaviour) and negligible diagenetic alteration.

We recalibrate the radiocarbon ages using the following ΔR values: (i) those used by the original authors (R=400 years, therefore ΔR=−5 years^82^; R=365±60 years, therefore ΔR=−40 years^83^); (ii) the values used by the original authors±100 year uncertainty (assuming a Gaussian distribution); (iii) the preindustrial ΔR estimated for the Caribbean^56^ (ΔR=−27±11 years, *n*=8; note, there are currently no observations from Barbados in the online ΔR database^56^); (iv) using model output values^84^ (using an iterative approach of transient, 3-dimensional simulations) that suggest variations in ΔR of 200 and 900 years for the Caribbean during the last deglacial. We use the upper and lower limits of their simulations with an arbitrary uncertainty of 100 years (i.e., ΔR=200±100 years and ΔR=900±100 years) using both a Gaussian and uniform distribution during calibration. Finally, we recalibrate the ages using temporally varying estimates of ΔR (derived from Butzin *et al.*^84^). Few of the of the recalibrated ages match the U-series ages for the samples, although the calibrated ages using the authors original estimates, preindustrial ΔR and those with no ΔR applied, offer a reasonable first approximation (Fig. 7). Using the modelled deglacial values for the Caribbean does not improve the match, although a variable ΔR does approximate the U-series ages slightly better than either of the model extremes (using both the Gaussian and uniform distributions). In this example, we are fortunate that the samples also possess U-series ages but it does illustrate the magnitude of the effect that choices regarding the ΔR value may have on the resulting age. This effect would be most acute during time intervals such as the last deglaciation, as major reorganisations in ocean circulation (as well as variations in ^14^C production and sequestration by the various reservoirs) are documented^85–87^. The sites in the database (i.e., primarily mid to low latitudes) should mitigate the magnitude of these effects because the scale of the oceanic changes (and hence ΔR) at those latitudes is smaller than at the higher latitudes^88^. The ‘distortion’ in age due to variations in ΔR is likely greater than the effects of uncertainties in both the tectonically corrected elevation (*Z*_*cp*_) and reconstructions of sea level probability (P_RSL_) for this interval of time, given the relatively low rates of both subsidence and uplift for most sites in the database, and the relatively young ages of the samples. The example illustrates the current difficulty in constraining ΔR through time. Therefore, we apply only the preindustrial estimates^56^ for the marine fossils when recalibrating ages in the database. Refinements in both the age determinations and reconstructed sea-level probability (P_RSL_) for radiocarbon-dated marine sea-level indicators could be achieved as more robust constraints on both the spatial and temporal variation in ΔR through time become available.

### Coral depth distributions

**Figure 8:**
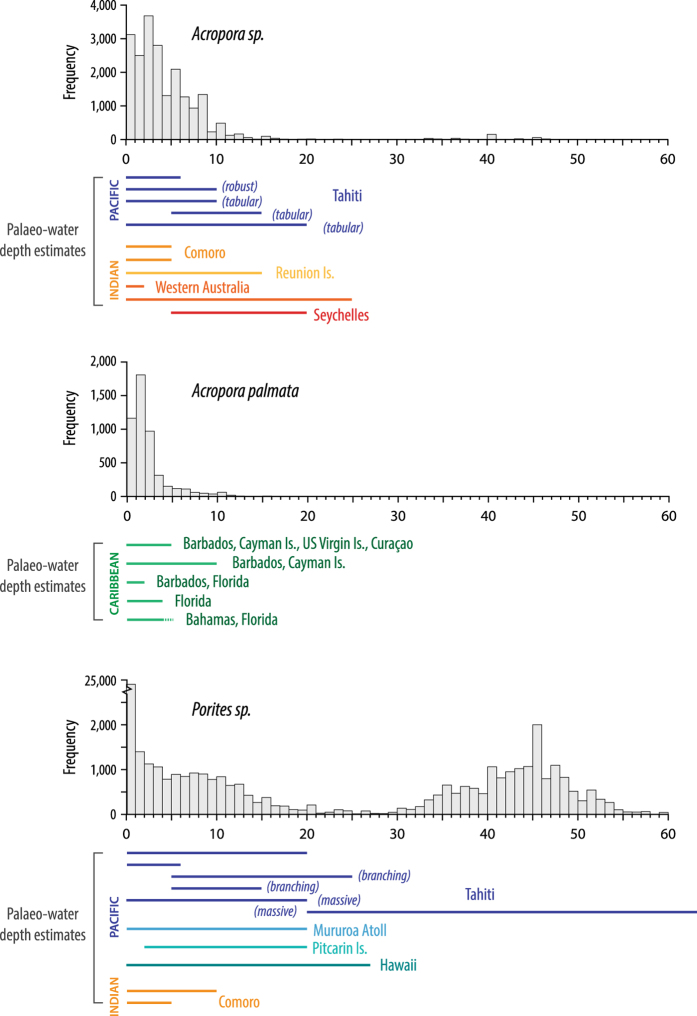
Coral depth distributions for three commonly dated species in the fossil database. The data uses observational and living data with a vertical depth precision of ≤ 0.25 m only. Coloured bars below each histogram are the palaeo-water depth estimates for various sites (grouped by ocean basin; blue=Pacific Ocean, orange=Indian Ocean, green=Caribbean) used by the original authors. Different coral growth forms are indicated by text in brackets.

We compare our ecologically derived depth distributions of modern corals to: (i) other estimates/observations of the maximum depth of coral species at both global^89,90^ and local geographic scales^75,91–97^ (Fig. 3) and; (ii) palaeo-water depth determinations of the original publications (Fig. 8). The median and 95% confidence limits derived compare favourably with both the global and regional (where available) modern observations of the maximum depth observed for most species (Fig. 3). This lends confidence that the use of our ecological depth distributions is reasonable and, that use of a modern-analogue approach provides a first-order approximation of the relationship between the elevation of the fossil coral and sea level at the time of formation.

**Figure 9:**
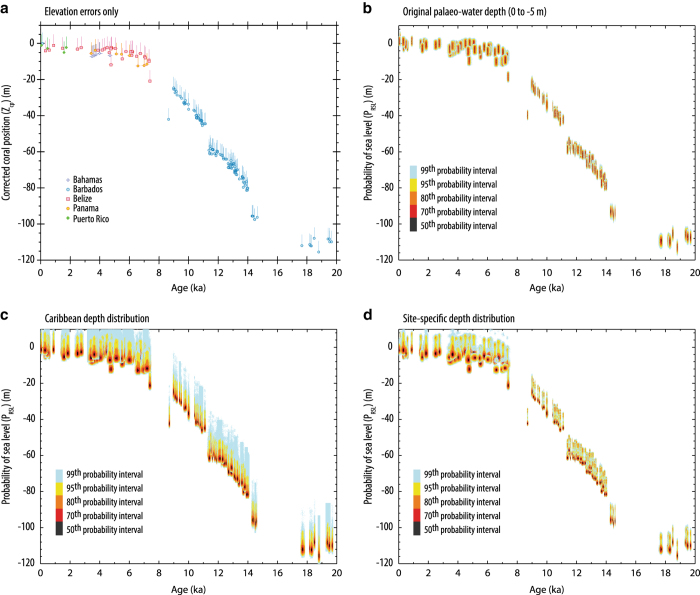
An example from the Caribbean (using the species *Acropora palmata*) of the effect of using different palaeo-water depth relationships on the resulting sea-level reconstructions. (**a**) the elevation uncertainties for the fossil *A. palmata* data; (**b**) the reconstructed sea level assuming a uniform distribution and a palaeo-water depth of 0 to 5 m; (**c**) the reconstructed sea level using our OBIS derived, ‘global’ species specific depth distribution and; (**d**) the reconstructed sea level using our regional depth distributions. P_RSL_ is reconstructed using a Monte-Carlo simulation of samples; coloured shading indicated the 99th (pale blue), 95th (yellow), 85th (orange), 70th (red) and 50th (black) percent probability intervals. This example is for illustrative purposes only and is not intended as a reinterpretation of the Caribbean *A. palmata* dataset.

The global, ecologically derived depth distributions also compare favourably with palaeo-water depth estimations, originally derived using a variety of methodologies (e.g., modern assemblage, coral diversity/distribution) and geographical scales (site-specific to ocean basin scale comparisons). Figure 8 illustrates for each of three commonly dated coral taxa our ecological depth distributions and the palaeo-water depths. The modern ‘global’ estimates broadly replicate the palaeo-water depths. However, our depth distributions are unlikely to capture the full complexity in species distribution and diversity observed in modern coral reefs, nor are they able to capture all details of the site-specific relationship between corals and sea level. Therefore, these ecological depth distributions should be considered as ‘maximum’, first-order approximations of the relationship between the elevation of the coral and sea level at the time of formation. The effect of using different depth distributions on reconstructed sea-level probability (P_RSL_) is illustrated for fossil *Acropora palmata* using data from the Caribbean (i.e., using the sub-basin and regional depth distributions) (Fig. 9). Once the elevation uncertainties are combined with either the palaeo-water depth estimates (assuming a 0 to 5 m depth preference and a uniform distribution, Fig. 9b) or the taxon-specific depth distributions (Fig. 9c), the regional depth distributions (Fig. 9d) result in ‘tighter’ P_RSL_ estimates for Barbados than either the palaeo-water depth or the Caribbean sub-basin depth distribution. Therefore, using a well-constrained, regional ecological depth distribution offers some promise of refining the vertical precision of reconstructed sea levels, and allows past sea levels to be reconstructed for samples where no information is available to define the relationship between the elevation of the fossil coral and sea level at the time of formation. Modern site-specific assemblage studies (i.e., documenting modern reef biota, facies and environmental characteristics) provide perhaps the best description of this relationship but our ecologically derived depth distributions (i.e., where only taxa and depth occurrence is given) offer a reasonable first-order approximation. Users of the database are able to use either the authors’ original palaeo-water depth determinations or our taxon depth distributions (at the ‘global’ or regional scale, Data Citation 2).

### Tectonic corrections

The only independent (i.e., not constrained using the fossil sea-level indicators themselves) tectonic corrections are those for Tahiti and Mururoa Atoll (both French Polynesia^16–18^). Hence, we are unable, so far, to validate the uplift/subsidence terms used in the database. This remains one of the main outstanding issues that hindering reconstructions of past sea level.

### Code availability

We make the code used to calculate P_RSL_ available as a separate text file (Data Citation 3). This contains significant modifications from that given as a supplement^12,98^ to incorporate a uniform facies formation depth distribution and non-Gaussian age uncertainties.

## Usage Notes

This release comprises 4 files (details of the file formats are within the square brackets):

1. Database [tab delimited file; ‘Data Citation 1’]
2. Summary of treatment of all the datasets compiled [text file; ‘SupplementaryData’]
3. Empirically derived coral depth distributions used to reconstruct sea level [tab delimited table; ‘Data Citation 2’]
4. Code: calculation of P_RSL_ for both ‘coral’ and ‘range’ type vertical uncertainties, as well as U-series and radiocarbon ages [pdf of Matlab code file; ‘Data Citation 3’]

We welcome contributions from authors of additional or clarifying information. These will be incorporated into any subsequent iteration of the database. When using data in this compilation, the original data collector(s) as well as the data compiler(s) should be credited^99^.

Users are welcome to use either the original authors’ (included in the database, Data Citation 1) or our ecologically derived depth distributions (Data Citation 2) to relate the elevation of the coral and sea level at the time of formation. Both are included in the database release, in addition to the code for reconstructing P_RSL_ (Data Citation 3).

No attempt has been made to correct for U-series open system behaviour, nor do we screen for age reliability. The inclusion of all metadata enables users to determine their own appropriate age reliability screening criteria. For simplicity, we record only the 68%, 95% confidence intervals, mean and sigma of the calibrated radiocarbon output. Again, the inclusion of all data and metadata relating to each radiocarbon determination enables users to both replicate our outputs and adapt the input into calibration software, if so desired. We do not attempt to account for temporal variations in ΔR.

The reconstructed P_RSL_ is a function of both eustatic and glacio-isostatic (GIA) processes. No correction has been made for GIA processes as this is outside the scope of this study.

## Additional information

**How to cite this article:** Hibbert F. D. *et al.* A database of biological and geomorphological sea-level markers from the Last Glacial Maximum to present. *Sci. Data* 5:180088 doi: 10.1088/sdata.2018.88 (2018).

**Publisher’s note:** Springer Nature remains neutral with regard to jurisdictional claims in published maps and institutional affiliations.

## Supplementary Material



Supplementary Information

## Figures and Tables

**Table 1:** Summary of datasets, by region, and all sources used to compile the sea-level database

**Region**	**Location(s)**	**Dating method(s)**	**Material(s) dated**	**Approx. temporal coverage (ka)**	**Number of samples**	**Source(s)**
Australia	Joseph Bonaparte Gulf	^14^C	Bivalve (marine); gastropod (marine); foraminifera; calcareous algae; wood; bulk sediment	0.5 to 45	75	DeDekker and Yokoyama, 2009^100^; Ishiwa et al., 2015^101^; Nicholas et al., 2014^102^; Yokoyama et al., 2000^103^, 2001^104^.
	Arafura Sea, Northern Territory	^14^C	Beachrock; shell (unspecified); wood	16.4	3	Jongsma, 1970^105^.
	New South Wales	^14^C, AAR, OSL	Bivalve (marine and unspecified); shells (estuarine and marine); charcoal; clay; quartz sand; peat; organic clay; wood	0.8 to 21 (and older)	18	Dury and Langford-Smith, 1968 (in Thom and Chappell, 1975^106^); Ferland et al., 1995^107^; Gill, 1967^108^; Langford Smith, 1970 (in Thom and Chappell, 1975^106^); Shepard, 1970^109^; Switzer et al., 2010^110^; Thom, 1965 (in Thom and Chappell, 1975^106^), 1969^111^, unpublished (in Thom and Chappell, 1975^106^); Thom and Chappell, 1975^106^.
	South Australia	^14^C	Bivalve (marine); gastropod (marine); mollusc (unspecified); shell hash; oyster; peat (mangrove); wood (mangrove); root fibres; seagrass; calcareous clay; fibre and organics from bulk sediment; organic mud	0.07 to 11	275	Belperio et al., 1983^112^, 1984^113^, 1993^114^, 2002^115^; Burne, 1982^116^; Harvey et al., 1999^117^; Short et al., 1986^118^.
	Victoria (Melbourne, Yarra delta)	^14^C	Shells (marine, estuarine); peat; wood	5.5 to 15	17	Bowler, 1966 (in Thom and Chappell, 1975^106^); Gill, 1967^108^, 1971 (in Thom and Chappell, 1975^106^); Gill and Hopley, 1972^119^; Thom and Chappell, 1975^106^.
	Tasmania	^14^C	wood	8.1	1	Gill, 1971 (in Thom and Chappell, 1975^106^).
	Queensland (including the Great Barrier Reef)	^14^C and U-series	Coral; coral microatoll; barnacle; beachrock; bivalve (marine); foraminifera; gastropod (marine); shell hash; peat; mangrove mud; wood (mangrove and unspecified); plant organics; organic clay; bulk sediment	0.07 to 17.5	258	Beaman et al., 1994^120^; Belperio et al.,1979^121^; Carter et al., 1993^122^, unpublished (in Woodroffe, 2009^123^); Chappell et al., 1983^124^; Grindrod and Rhodes, 1984^125^; Harvey et al.,2001^126^; Horton et al., 2007^127^; Kench et al., 2012^128^; Larcombe and Carter, 1998^129^; Larcombe et al., 1995^130^; Leonard et al., 2016^131^; Lewis et al., 2008^132^; 2012^133^, 2015^134^; Ohlenbusch, 1991^135^; Spenceley, 1980^136^; Thom, unpublished (in Thom and Chappell, 1975^106^); Tye, 1992^137^; Veeh and Veevers, 1970^138^; Woodroffe, 2009^123^; Yu and Zhao, 2010^139^.
	Torres Strait	^14^C	Coral; coral microatoll	1.3 to 8	27	Woodroffe et al., 2000^140^.
New Zealand	North Island	^14^C	Bivalve (marine); shell (unspecified); peat; wood; carbonate mud	0.15 to 11	151	Berryman, unpublished; Boag, unpublished, Brown, unpublished; Ghani, unpublished (all in Ota et al., 1988^141^); Cox, unpublished, (in Gibb, 1986^142^); Gibb, 1979^143^, 1986^142^; Leach, 1984^144^; Leach and Anderson, 1974^145^; McFadgen, 1980^146^; Mildenhall, 1979^147^; Ota et al., 1983^148^, 1988^141^, 1991^149^; Schofield, 1960^150^; Singh, 1971^151^; Woodroffe et al., 1983^152^; Yoshikawa et al., 1980^153^.
	South Island	^14^C	Peat; shell (estuarine and unspecified); wood	0.4 to 11	32	Brown, 1973^154^, unpublished (in Gibb, 1986^142^); Gibb, 1986^142^; Landis, unpublished (in Gibb, 1986^142^); Suggate, 1968^155^.
Austral Islands	Rurutu, Tubuai	^14^C	Coral microatoll; coralline algae	1.6 to 2.5	3	Pirazzoli and Montaggioni, 1988^156^.
Gambier Islands	Gambier, Temoe	^14^C	Coral; coral microatoll; coralline algae	0.8 to 4.2	5	Pirazzoli, 1987^157^; Pirazzoli and Montaggioni, 1987^158,159^; Pirazzoli and Montaggioni, 1988^156^.
Society Islands	Bora Bora; Huahine; Maupiti; Mopelia; Raiatea; Tahiti; Tupai	^14^C	Coral; reef framework; coral microatoll	0.9 to 4.7	16	Chevalier and Salvat, 1976^160^; Pirazzoli and Montaggioni, 1988^156^; Pirazzoli et al., 1985^161^; Salvat et al., 1977^162^; see also references in the compilation of Hibbert et al., 2016^12^..
Tuamotu Isalnds	Amanu; Anaa; Faaite; Hao; Hereheretue; Hikuere, Kaukura; Makatea; Mataiva; Mururoa; Nukutavake; Nukutipipi; Pukarua; Rangiroa, Reao; Tiararo; Takapoto; Tureia; Vahitahi; Vairaatea	^14^C	Bivalve; coral; reef framework; coral microatoll	0.3 to 6.1	55	Chevalier and Salvat, 1976^160^; Delibrias et al., 1974^163^; Montaggioni, 1985^164^; Pirazzoli, 1985^165^; Pirazzoli and Montaggioni, 1984^166^, 1986^167^, 1988^156^; Pirazzoli et al., 1987^158,168^; 1988^169,170^; Salvat et al., 1977^162^.
Southern Cook Islands	Aitutaki; Mangaia Island; Rarotonga	^14^C and U-series	Coral; coral microatoll	0 to 5.8	41	Allen et al., 2016^171^; Goodwin and Harvey, 2008^172^; Moriwaki et al., 2006^173^; Yonekura et al., 1988^174^.
South America	Argentine Shelf	^14^C	Shells, shell hash	8.4 to 19.6 (and 31 to 55)	46	Guilderson et al., 2000^175^.
Indian Ocean	Comoro Archipelago, Mayotte Island (multiple sites)	^14^C and U-series	Bivalve; coral; gastropod; oyster; organics	0.7 to 20 (and 27 to 33)	67	Camoin et al., 1997^176^; Colonna et al., 1996^177^; Zinke et al., 2003^178^.
	Mauritius	^14^C and U-series	coral	1.5 to 8	39	Camoin et al., 1997^176^; Montaggioni and Faure, 1997^179^.
	Maldives	^14^C	Beachrock; coral; coral sand; coral microatoll; reef rock; sand (skeletal carbonates)	0.04 to 8	99	Gischler et al., 2008^180^; Kench et al., 2005^181^, 2009^182^; Woodroffe, 1993^183^.
	Reunion Island	^14^C	coral		8	Camoin et al., 1997^176^.
	Tanzania, Zanzibar	^14^C	Organic mangrove concentrates	2 to 7.8	8	Woodroffe et al., 2015^184^.
Indian subcontinent	Bangladesh, Bay of Bengal	^14^C	Ooids; mollusc shell (unspecified)	19.8 to 24	5	Wiedicke et al., 1999^185^.
	India (southern)	^14^C	Bivalve (marine); coral; foraminifera; gastropod (marine); shells (unspecified)	0.04 to 6.8 (and 27 to 35)	20	Banerjee, 2000^186^.
	Sri Lanka	^14^C	Bivalve (marine); coral; gastropod (marine)	2.2 to 7	42	Katupotha and Fukiwara, 1988^187^.
Southern Africa	South Africa	^14^C and U-series	Aeolianite; beachrock; bivalve (unspecified); calcareous algae; coral; elephant tusk; oyster; peat; shells (unspecified); wood	1 to 11.3 (and 15 to 19, 30 and 43)	42	Grobbler et al., 1998^188^; King, 1972^189^; Maud, 1968^190^, unpublished (in Ramsay and Cooper, 2002^191^); Miller et al., 1995^192^; Ramsay, 1991^193^, 1996^194^; Ramsay and Cooper, 2002^191^; Ramsay and Mason, 1990^195^; Reddering, 1988^196^; Siesser, 1974^197^; Vogel and Marais, 1971^198^; Vogel and Visser, 1981^199^; Yates et al., 1986^200^.
	Mozambique	^14^C	Beachrock cement	Can’t recalculate	2	Siesser, 1974^197^; Ramsay, 1996^194^.
SE Asia	Sunda Shelf	^14^C	Macro-fibres; peat; root fibres; wood; bulk sediment	13 to 21	37	Hanebuth et al., 2000^201^, 2009^202^.
	Japan	^14^C	Bivalve (marine); plant fragment; wood	1 to 14 (and 41 to 45)	54	Tanabe et al., 2009^203^.
	China	^14^C	Beachrock; bivalve (marine); coral; coral sand; organic sediment (lagoon and unspecified); marsh sediments; shell-ridge conglomerate; shells (unspecified)	0.2 to 9.8	235	Chen and Lui, 1996^204^; Chen et al., 1982^205^; Dai, 1987^206^; Hong 1990^207^; Huang et al., 1986^208^; Li et al., 1991^209^; Sun and Huang, 1993^210^; Wang, 1991^211^; Yang, 1986^212^; Yim, 1986^213^; Zhang and Lui, 1987^214^; Zhao, 1996^215^; Zhu et al., 1996^216^; Zong, 1992^217^, 2004^218^.
	China (Bohai Sea)	^14^C	Bivalve (marine); echinod; gastropod (marine); shells (unspecified)	0.4 to 10 (and 14 and 39)	30	Saito et al., 2000^219^.
	China (East China Sea)	^14^C	Bivalve (marine); gastropod (marine); mollusc (unspecified); plant material	0.1 to 12	17	Liu et a., 2010^220^.
	China (South China Sea)	U-series	Coral microatoll	6.7 to 7.2	18	Yu et al., 2009^221^.
	China (Yellow Sea)	^14^C	foraminifera	5.8 to 13	2	Kim and Kennet, 1998^222^.
	Korea (Yellow Sea)	^14^C	Foraminifera; gastropod (unspecified); oyster; peat	0.04 to 8.8	5	Kim and Kennet, 1998^222^.
	Thailand	^14^C	Coral microatoll; peat; wood; organic mud	1.8 to 8.6	44	Chaimanee et al., 1985^223^; Horton et al., 2005^224^; Scoffin and le Tissier, 1998^225^; Sinsakul, 1992^226^; Somboon and Thiramongkol, 1992^227^; Thiramongkol, 1984^228^; Tiyapunte and Theerarungsikul, 1988^229^.
	Malaysia	^14^C	Oyster; peat; wood	1 to 9.5	29	Geyh et al., 1979^230^; Hassan, 2001^231^; Horton et al., 2005^224^; Tjia et al., 1983^232^.
	Singapore	^14^C	Bivalve (marine and unspecified); coral; gastropod (marine); oyster; peat (mangrove); roots; shells (unspecified); wood	0.1 to 9.4	105	Bird et al., 2007^233^, 2010^234^; Hesp et al., 1998^235^.
	Vietnam (including Vietnam Shelf)	^14^C	Bivalve; gastropod; coral	0.6 to 7.2	23	Hanebuth et al., 2000^201^; Michelli, 2008^236^.
Caribbean	Barbados	U-series	Coral	8.6 to 14 (and older)	110	Abdul et al., 2016^237^; Fairbanks et al., 2005^83^; Mortlock et al., 2005^238^; Peltier and Fairbanks, 2006^239^; see also references in the compilation of Hibbert et al., 2016^12^.
	Jamaica	^14^C	Peat (mangrove, sedge, swamp forest)	0.5 to 9	55	Digerfeldt and Hendry, 1987^240^; Toscano and Macintyre, 2003^241^.
	Belize	^14^C and U-series	Beachrock; bivalve (marine); calcareous (Halimeda) sand; coral; gastropod (marine); mangrove leaves; peat (mangrove); roots; sediment; soil; wood	0.05 to 11.5	190	Gischler, 2003^242^; Gischler and Husdon, 1998^243^, 2004^244^; Gischler and Lomando, 1997^245^, 2000^246^; Halley et al., 1977^247^; Macintyre et al., 1995^248^, 2004^249^; Monacci et al., 2009^250^; Shinn et al., 1982^251^; Toscano and Macintyre, 2003^241^; Wooller et al., 2004^252^, 2007^253^, 2009^254^.
	Florida	^14^C and U-series	Coral; peat (brackish, freshwater, mangrove and unspecified); shells (marine and freshwater); ‘calcitic mud’; bulk sediment	0.4 to 10.8	142	Banks et al., 2007^255^; Lighty et al., 1978^256^, 1982^257^; Multer et al., 2002^258^; Precht et al, unpublished (in Toscano and Macintyre, 2003^241^); Robbin, 1984^259^; Scholl and Stuiver, 1967^260^; Stathakopoulos and Riegl, 2015^261^; Toscano and Macintyre, 2003^241^.
	Bahamas	^14^C	Calcarenite; coral; gastropod (marine, vermetid); crustose coralline algae	0.2 to 5.2	18	Lighty et al., 1982^257^; Macintyre et al., 1996^262^.
	Martinique	^14^C	coral	0.6 to 2.2	5	Adey, unpublished (in Lighty et al., 1982^257^); Adey and Burke, 1976^263^; Lighty et al., 1982^257^.
	Puerto Rico	^14^C	coral	0.8 to 2.2	4	Lighty et al., 1982^257^; Macintyre et al., 1983^264^.
	Panama	^14^C	coral	4 to 7.6	8	Macintyre and Glynn, 1976^265^; Lighty et al., 1982^257^.
	Grand Cayman	^14^C	Peat (mangrove)	0.6 to 2.2	9	Woodroffe, 1981^266^.
	Antigua	^14^C	coral	0.6 to 7.6	10	Macintyre et al., 1985^267^.
	US Virgin Islands, St Croix	^14^C and U-series	Coral; shells (unspecified)	0.1 to 10.3	71	Adey, 1975^268^, unpublished (in Lighty et al., 1982^257^); Adey et al., 1977^269^; Burke et al., 1989^270^; Hubbard et al., 2005^271^; Lighty et al., 1982^257^; Macintyre and Adey, 1990^272^; Macintyre et al., 2008^273^.
	Trinidad	^14^C	Mangrove peat (assumed, not reported)	0.6 to 7	9	Ramcharan, 2004^274^; Ramcharan and McAndrews, 2006^275^.
	Bermuda	^14^C	peat	0.8 to 4.4 (and 10.3)	17	Redfield, 1967^276^.

**Table 2:** Uplift and subsidence rates used in the database and their derivation.

**Location**	**Site**	**Tectonic category**	**Rate used (m/ka) (2dp)**	**Derivation**
*Caribbean*				
Barbados	core off south coast	uplifting	0.34±0.02	Literature (Fairbanks, 1989^277^)
Trinidad	Maracas Swamp, NW coast	subsiding	−0.07±0.02	Literature/recalculation: submerging coastline with subsidence thought to equal that of the uplift in the NE coastline (subsidence rate of −0.02 to −1.5 m/ka, Weber, 2010^278^) based on an emergent Pleistocene marine terrace at +15 m (Kugler, 1961^279^) but the age is poorly constrained; assuming this is of Last Interglacial age would give uplift rate of 0.0672±0.02 m for the NE coastline (and therefore subsidence of −0.0672±0.02 m/ka).
US Virgin Islands	St Croix (various)	subsiding	−0.11±0.02 *	Max elevation of the Last Interglacial reef (Toscano et al., 2012^280^)
				
*South America*				
Argentine Shelf	Various	uplifting	−0.08±0.16	Literature (Guilderson et al., 2000^175^, supplementary materials)
				
*Indian Ocean*				
Bangladesh	Bengal Shelf	subsiding	−0.2 (no uncertainty given, used±0.2 m/ka uncertainty)	Literature: −0.2 m/ka since the Pliocene (Biswas, 1992^281^)
Comoro Archipelago	Mayotte Island (various)	subsiding	−0.21±0.02 *	Max elevation of the Last Interglacial reef (Camoin et al., 1997^176^)
Mauritius	Pointe aux Sables reef	subsiding	−0.04±0.02 *	Max elevation of the Last Interglacial reef (Montaggioni, 1978^282^, 1988^283^)
Reunion Island	La Saline reef	subsiding	−0.04 (no uncertainty given, allocated a±0.1 m uncertainty)	Literature: rate based on the occurrence of a MIS 7.5 reef deposit (coral rubble) and the assumption this deposit correlates to a similar deposit on Madagascar (tectonically stable). The difference in the elevation between the two deposits between the two sites (Reunion Island and Madagascar) is then used as the subsidence rate of Reunion Island since MIS 7.5 (assumed age=250 ka)^282^; ‘Subsidence rate very low and probably around 0.03 m/ka’ Montaggioni, *pers. comm*.).
				
*Oceania and Pacific Ocean*				
Japan	Echigo Plain	subsiding	−2.96±0.18	Literature (Tanabe et al., 2009^203^)
Austral Islands	Rurtu	uplifting	0.02±0.93	Recalculated using the elevation notch (+8 to +9 m) assumed to be Last Interglacial in age (dated at 122 ka) Note, rate given as 0.05 to 0.1 m/ka (Pirazzoli and Salvat, 1992^284^)
	Tubuai	subsiding	−0.03±0.02	No rate given in the publication; used the same rate as Makatea (Tubuai subsiding but give no rate given other than ‘slightly faster than in most Tuamotu atolls’, Pirazzoli and Salvat, 1992^284^).
Southern Cook Islands	Aitutaki (various)	subsiding	−0.03±0.02 *	Max elevation of the Last Interglacial reef (Goodwin and Harvey, 2008^172^)
	Mangaia	uplifting	0.07±0.02 *	Max elevation of the Last Interglacial reef (Woodroffe et al., 1981^285^Spencer et al., 1988^286^)
	Rarotonga	subsiding	−0.02±0.01 *	Max elevation of the Last Interglacial reef (Stoddart et al., 1985^287^; Spencer et al., 1987^288^; Dickinson, 1998^289^)
Society Islands	Bora Bora	subsiding	−0.05±0.02 *	Recalculated using the max. elevation *of in situ* corals assumed to be Last Interglacial in age (Pirazzoli et al., 1985^161^; Rashid, 2014^290^)
	Huahine	subsiding	−0.05±0.02 *	Recalculated using the max. elevation *of in situ* corals assumed to be Last Interglacial in age (Pirazzoli et al., 1985^161^; Rashid, 2014^290^)
	Mopelia	unknown; assumed to be subsiding	−0.05±0.02	Used same value as for other Society Islands
	Mururoa Atoll	subsiding	−0.08±0.01	Independent estimate (radiometrically dated volcanic lava flow) (mid-point; Trichet et al., 1984^18^)
	Maupiti	subsiding	−0.05±0.02 *	Recalculated using the max. elevation of *in situ* corals assumed to be Last Interglacial in age (+0.4 m; Pirazzoli et al., 1985^161^)
	Raiatea	subsiding	−0.05±0.02 *	Recalculated using the max elevation of in situ corals assumed to be of Last Interglacial age (+0.15 m; Pirazzoli et al., 1985^161^)
	Tahiti	subsiding	−0.30±0.10	Independent estimate (radiometrically dated volcanic lava flow) (mid-point; Deschamps et al., 2012^21^)
	Tupai	subsiding	−0.05±0.02 *	Recalculated rate using the max elevation of in situ corals assumed to be of Last Interglacial age (+0.5 m; Pirazzoli et al., 1985^161^)
Tuamotu Islands	Amanu	unknown	can’t calculate	No further information available relating to the tectonic correction required for this site
	Anaa	subsiding	−0.02±0.02 *	Recalculated the rate using the max elevation of the Last Interglacial terrace (+4m) (Veeh, 1966^291^; Pirazzoli et al., 1988^169^). Note, Pirazzoli et al., 1988^169^ state site is uplifting.
	Faaite	unknown	can’t calculate	No further information available relating to the tectonic correction required for this site
	Hao	unknown	can’t calculate	No further information available relating to the tectonic correction required for this site
	Hereheretue	unknown	can’t calculate	No further information available relating to the tectonic correction required for this site
	Hikuera	unknown	can’t calculate	No further information available relating to the tectonic correction required for this site
	Kaukura	unknown	can’t calculate	No further information available relating to the tectonic correction required for this site
	Makatea	subsiding	−0.03±0.02 *	Recalculated the rate using the max elevation of the Last Interglacial terrace (+3 m; Veeh, 1966^291^). Note, Pirazzoli et al., 1985^161^, 1988^156^ state site is uplifting.
	Mataiva	unknown	can’t calculate	No further information available relating to the tectonic correction required for this site
	Mururoa	subsiding	−0.08±0.01	Used the mid-point of the subsidence rate range (Trichet et al., 1984^18^)
	Nukutavake	unknown	can’t calculate	No further information available relating to the tectonic correction required for this site
	Nukutipipi	unknown	can’t calculate	No further information available relating to the tectonic correction required for this site
	Pukarua	unknown	can’t calculate	No further information available relating to the tectonic correction required for this site
	Rangiroa	subsiding	−0.02±0.001 *	Recalculated using reef fossils found in volcanogenic silt- stones at DSDP Site 318 (Schlanger et al.,1976^292^) and an age of 50 Ma (Talandier and Okal, 1987^293^).
	Reao	unknown	can’t calculate	No further information available relating to the tectonic correction required for this site
	Taiaro	unknown	can’t calculate	No further information available relating to the tectonic correction required for this site
	Takapoto	unknown	can’t calculate	No further information available relating to the tectonic correction required for this site
	Vahitahi	unknown	can’t calculate	No further information available relating to the tectonic correction required for this site
	Vairaatea	unknown	can’t calculate	No further information available relating to the tectonic correction required for this site
New Zealand, North Island	Akitio River†	uplifting	0.75±0.25	Literature: stable or uplifting at ≥0.5 m/ka but > 1 m/ka (Pillans, 1986^294^)
	Aramoana†	uplifting	0.75±0.25	Literature: stable or uplifting at ≥0.5 m/ka but > 1 m/ka (Pillans, 1986^294^)
	East Cape†	uplifting	1±0.25	Literature: >1 m/ka (Pillans, 1986^294^)
	Gisborne†	uplifting	0.25±0.25	Literature: uplifting at less than 0.5 mm/yr (Pillans, 1986^294^)
	Hastings†	uplifting	0.75±0.25	Literature: stable or uplifting at ≥0.5 m/ka but > 1 m/ka (Pillans, 1986^294^)
	Havelock North†	uplifting	0.75±0.25	Literature: stable or uplifting at ≥0.5 m/ka but > 1 m/ka (Pillans, 1986^294^)
	Hicks Bay†	uplifting	0.75±0.25	Literature: stable or uplifting at ≥0.5 m/ka but > 1 m/ka (Pillans, 1986^294^)
	Kaiaua†	uplifting	0.25±0.25	Literature: uplifting at less than 0.5 mm/yr (Pillans, 1986^294^)
	Kaiwhata River†	uplifting	0.75±0.25	Literature: stable or uplifting at ≥0.5 m/ka but > 1 m/ka (Pillans, 1986^294^)
	Karaka Bay†	uplifting	1±0.25	Literature: >1 m/ka (Pillans, 1986^294^)
	Kellys Beach†	uplifting	0.25±0.25	Literature: uplifting at less than 0.5 mm/yr (Pillans, 1986^294^)
	Kumenga†	subsiding	n/a	Literature: no rate given (Pillans, 1986^294^)^294^
	Mahia Peninsula†	uplifting	0.25±0.25	Literature: uplifting at less than 0.5 mm/yr (Pillans, 1986^294^)
	Mataikona River†	uplifting	0.25±0.25	Literature: uplifting at less than 0.5 mm/yr (Pillans, 1986^294^)
	Miranda†	subsiding	n/a	Literature: no rate given (Pillans, 1986^294^)
	Opouawe River†	uplifting	1±0.25	Literature: >1 m/ka (Pillans, 1986^294^)
	Oterei River†	uplifting	1±0.25	Literature: >1 m/ka (Pillans, 1986^294^)
	Owahanga River†	uplifting	0.75±0.25	Literature: stable or uplifting at ≥0.5 m/ka but > 1 m/ka (Pillans, 1986^294^)
	Pahaoa River†	uplifting	1±0.25	Literature: >1 m/ka (Pillans, 1986^294^)
	Pakarae River†	uplifting	1±0.25	Literature: >1 m/ka (Pillans, 1986^294^)
	Patanui Stream†	uplifting	0.75±0.25	Literature: stable or uplifting at ≥0.5 m/ka but > 1 m/ka (Pillans, 1986^294^)
	Pauatahanui Inlet†	uplifting	0.75±0.25	Literature: stable or uplifting at ≥0.5 m/ka but > 1 m/ka (Pillans, 1986^294^)
	Pourerere†	uplifting	1±0.25	Literature: >1 m/ka (Pillans, 1986^294^)
	Sponge Bay†	uplifting	0.25±0.25	Literature: uplifting at less than 0.5 mm/yr (Pillans, 1986^294^)
	Tolaga Bay†	uplifting	1±0.25	Literature: >1 m/ka (Pillans, 1986^294^)
	Uruti Point†	uplifting	0.75±0.25	Literature: stable or uplifting at ≥0.5 m/ka but > 1 m/ka (Pillans, 1986^294^)
	Waihau Bay†	uplifting	1±0.25	Literature: >1 m/ka (Pillans, 1986^294^)
	Waimoana†	uplifting	0.75±0.25	Literature: stable or uplifting at ≥0.5 m/ka but > 1 m/ka (Pillans, 1986^294^)
	Wainui Beach†	uplifting	0.25±0.25	Literature: uplifting at less than 0.5 mm/yr (Pillans, 1986^294^)
	Waipapa Stream†	uplifting	0.75±0.25	Literature: stable or uplifting at ≥0.5 m/ka but > 1 m/ka (Pillans, 1986^294^)
	Wairoa†	uplifting	0.25±0.25	Literature: uplifting at less than 0.5 mm/yr (Pillans, 1986^294^)
	Whakaki†	uplifting	0.25±0.25	Literature: uplifting at less than 0.5 mm/yr (Pillans, 1986^294^)
	Whakataki River†	uplifting	0.75±0.25	iterature: stable or uplifting at ≥0.5 m/ka but > 1 m/ka (Pillans, 1986^294^)
	White Rocks†	uplifting	1±0.25	Literature: >1 m/ka (Pillans, 1986^294^)
New Zealand, South Island	Christchurch†	subsiding	−0.35±0.15	Literature: Canterbury Margin subsiding at 0.2 to 0.5 m/ka (Browne and Naish, 2003^295^)

*rate recalculated using the max. elevation of the Last Interglacial reef; an assumed age of 125±5 ka and sea level of 6.6±2 m^329,330^

^†^original authors derive rate by fitting the data to the sea level curve of Gibb, 1986^142^.

**Table 3:** Allocated elevation-, sample extraction- and sampling uncertainties (where these are missing from the original publication.

**Method**	**Max. quoted uncertainty (m,±2σ)**	**Min. quoted uncertainty (m,±2σ)**	**Allocated uncertainty (m,±2σ)**	**derivation**
*1. Elevation determination:*				
Auto-level	not reported	not reported	0.03	cf. levelling uncertainty (Törnqvist *et al.*^22^; Hijma *et al.*^296^)
clinometer and analog depth recording	not reported	not reported	half modern tidal range	n/a
digital depth gauge/ dive computer	not reported	not reported	0.5	Rovere *et al.*^297^; Azzopardi and Sayer^298^
interpolation between contours on drainage plans	variable	variable	dependent on contour spacing	n/a
interpolation from topographic maps; no contour spacing given	variable	variable	0.5	n/a
levelling	1.5	0.01	0.03	Törnqvist *et al.*^22^; Hijma *et al.*^296^
levelling (laser)	0.15	0.15	0.03	Törnqvist *et al.*^22^; Hijma *et al.*^296^
ship—echosounder/not reported	not reported	not reported	half modern tidal range	n/a
‘spirit level and folding ruler’	not reported	not reported	0.5	n/a
survey, not reported	n/a	n/a	0.5	n/a
theodolite	not reported	not reported	0.03	cf. levelling uncertainty (Törnqvist *et al.*^22^; Hijma *et al.*^296^)
unknown or not reported	n/a	n/a	0.5	n/a
				
*2. Coring method:*				
Auger or hand auger	not reported	not reported	0.05	cf. hand coring (Hijma *et al.*^296^; Woodroffe^299^)
horizontal push core	not reported	not reported	0.15	cf. vibracoring and rotary drilling (Hijma *et al.*^296^; Morton and White^300^)
hydraulic drill or piston core	not reported	not reported	0.15	cf. vibracoring and rotary drilling (Hijma *et al.*^296^; Morton and White^300^)
piston corer, Livingstone, split spoon or unspecified	not reported	not reported	0.15	cf. vibracoring and rotary drilling (Hijma *et al.*^296^; Morton and White^300^)
Russian corer	not reported	not reported	0.01	Woodroffe^299^
star picket driver	not reported	not reported	0.15	cf. vibracoring and rotary drilling (Hijma *et al.*^296^; Morton and White^300^)
single tube sampler	not reported	not reported	0.15	cf. vibracoring and rotary drilling (Hijma *et al.*^296^; Morton and White^300^)
percussion drilling	not reported	not reported	0.15	cf. vibracoring and rotary drilling (Hijma *et al.*^296^; Morton and White^300^)
rotary drill	not reported	not reported	0.15	cf. vibracoring and rotary drilling (Hijma *et al.*^296^; Morton and White^300^)
SCARID drilling system	Not reported	not reported	0.1	Dennis Hubbard (pers. comm)
barge mounted drilling rig	not reported	not reported	0.15	cf. vibracoring and rotary drilling (Hijma *et al.*^296^; Morton and White^300^)
virbracore	not reported	not reported	0.15	Hijma *et al.*^296^; Morton and White^300^
gravity corer	not reported	not reported	0.15	cf. vibracoring and rotary drilling (Hijma *et al.*^296^; Morton and White^300^)
‘rigging’—unknown	not reported	not reported	0.15	cf. vibracoring and rotary drilling (Hijma *et al.*^296^; Morton and White1997^300^)
drilling, unspecified	not reported	not reported	0.15	cf. vibracoring and rotary drilling (Hijma *et al.*^296^; Morton and White^300^)
not reported	0.5	0.03	0.15	cf. vibracoring and rotary drilling (Hijma *et al.*^296^; Morton and White^300^)
not reported, assumed hand coring	not reported	not reported	0.05	Hijma *et al.*^296^; Woodroffe^299^
not reported, assumed vibracoring	not reported	not reported	0.15	Brown^154^; Robbin^259^
				
*3. Sampling:*				
author specified	0.001	2	n/a	n/a
cores	0.001	2	0.01	Shennan^14^
exposure/outcrop	0.01	0.01	0.01	n/a
unknown/not reported setting	0.25	0.25	0.01	n/a

**Table 4:** ΔR values and, their derivation, used in the recalibration of marine radiocarbon data in the database

**Location**	**Site**	**n**	**ΔR**	**±1σ**	**source(all recalculated using Reimer and Reimer, 2018**^56^)
*Caribbean*					
Antigua		45	16	40	Hughen et al., 2004^85^; Kilbourne et al., 2007^301^; Wagner et al., 2009^302^
Bahamas		3	25	91	Lighty et al., 1982^257^; Broecker et al., 1961^303^
Belize		28	−16	31	Druffel, 1980^304^
Martinique		45	16	40	Hughen et al., 2004^85^; Kilbourne et al., 2007^301^; Wagner et al., 2009^302^
Panama		75	6	40	Broecker et al., 1961^303^; Druffel, 1980^304^; Hughen et al., 2004^85^; Kilbourne et al., 2007^301^; Wagner et al., 2009^302^
Puerto Rico		41	28	36	Kilbourne et al., 2007^301^
US Virgin Is	St Croix	41	28	36	Kilbourne et al., 2007^301^
US, Florida	Florida Keys	139	−1	56	Druffel and Linick, 1978^305^; Druffel, 1997^306^; Lighty et al., 1982^257^
					
*Indian Ocean*					
South Africa	Eastern-Agulhas type	3	155	30	Southon et al., 2002^307^; Wündsch et al., 2016^308^
	Western-Benguela-type	9	157	59	Dewar et al., 2012^309^; Southon et al., 2002^307^
Maldives		17	135	76	Southon et al., 2002^307^
Mauritius		2	122	5	Southon et al., 2002^307^
Mozambique		9	170	84	Southon et al., 2002^307^
Bangladesh		7	71	64	Dutta et al., 2001^310^; Southon et al., 2002^307^
India, south		7	71	64	Dutta et al., 2001^310^; Southon et al., 2002^307^
Sri Lanka		4	133	65	Delibrias et al., 1974^163^
					
*S.E. Asia*					
Japan		12	−87	67	Hirabayashi et al., 2017^311^; Yoneda et al., 2007^312^
Korea		2	−100	20	Kong and Lee, 2005^313^; Southon et al., 2002^307^
Malaysia, Peninsula		61	17	15	Bolton et al., 2016^314^; Dang et al., 2004^315^; Southon et al., 2002^307^
China	Bohai Sea	1	−178	50	Southon et al., 2002^307^,^307^
	E. China Sea	22	31	84	Hirabayashi et al., 2017^311^; Yoneda et al., 2007^312^
	S. China Sea	71	19	18	Bolton et al., 2016^314^; Dang et al., 2004^315^; Southon et al., 2002^307^; Yoneda et al., 2007^312^
	Yellow Sea	2	−100	20	Kong and Lee, 2005^313^; Southon et al., 2002^307^
Singapore		2	−45	68	Southon et al., 2002^307^
Thailand		1	−19	70	Southon et al., 2002^307^
Vietnam		59	18	14	Bolton et al., 2016^314^; Dang et al., 2004^315^; Southon et al., 2002^307^
					
*Oceania and Pacific Ocean*					
Australia	South Australia	7	62	61	Bowman, 1985^316^; Gillespie and Polach, 1979^317^
	Victoria, Melbourne, Tasmania	1	−14	120	Gill, 1983^318^
	NSW coast	1	11	85	Gillespie and Polach, 1979^317^
	Queensland (open ocean, near shore)	12	11	14	Gillespie and Polach, 1979^317^
	NT, Kimberly, Bonaparte Gulf	14	58	22	Bowman, 1985^319^; O’Connor et al., 2010^320^; Southon et al., 2002^307^
New Zealand	east coast S. Island	2	−5	47	Higham and Hogg, 1995^321^; Rafter et al., 1972^322^
	Christchurch	1	−25	35	Higham and Hogg, 1995^321^
	east coast, N. Island	11	10	25	McSaveney et al., 2006^323^; Sikes et al., 2000^324^
	north coast, N. Island	7	12	56	Higham and Hogg, 1995^321^; Sikes et al., 2000^321,324,324^
	southern tip of N. Island	15	−7	31	Higham and Hogg, 1995^321^; McSaveney et al., 2006^323^
Austral Islands		1	−3	17	Petchey et al., 2008^325^
Comoro Archipelago	Mayotte	1	119	57	Southon et al., 2002^307^
South Cook Islands		3	−15	38	Guilderson et al., 2000^326^; Petchey et al., 2008^325^
Gambier Islands		3	−2	23	Petchey et al., 2008^325^
Society Islands		6	17	21	Broecker et al., 1961^303^; Petchey et al., 2008^325^
Tuamotu Islands		1	6	17	Petchey et al., 2008^325^
Values are calculated using the ΔR values and uncertainties in the online database^56^. Where more than one sample is used, we calculate an error weighted mean and uncertainty. Recalculated ΔR values are cross referenced with previous determinations (note, this may result in some differences between the values derived by the original authors but ensures the both the correct (and consistent) calculation of ΔR).					

**Table 5:** Summary statistics for the empirically derived, modern depth distributions of selected coral species (note, only species also within the fossil database are detailed)

**Taxa**	**Number of observations**	**Max depth (m)**	**Min depth (m)**	**Median depth (m)**	**depth range (m) (95%)**	**depth range (m) (68%)**	**Lower error (68%)**	**Upper error (68%)**	**Lower error (95%)**	**Upper error (95%)**
*Acropora* sp.	20784	1416	0	3	17.1	6	4	2	14.1	3
*A. abrontanoides* ^*(a)*^	132	49.5	0	4	49.5	38	35	3	45.5	4
*A. cervicornis*	893	48	0.2	4.6	15.6	8.1	5.4	2.7	11.5	4.1
*A. humulis*	523	46	0	3	36	6	4	2	33	3
*A. hyanthis*	653	11	0	3	10	5	4	1	7	3
*A. palmata*	4884	22	0	1.5	8.9	2.3	1.5	0.8	7.8	1.2
*A. robusta*	126	9	0	4	8	7	4	3	4	4
*Alveopora* sp. ^*(b)*^	171	50	2	5	43.7	42.5	39.5	3	40.7	3
*Astreopora* sp.	1672	66	0	13	45	41	28	13	32	13
*Montipora* sp.	28704	1013	0	0	56	45	45	0	56	0
*Agaricia* sp.	6608	349	0	8.8	18.9	11.4	4.7	6.8	11.3	7.7
*A. agaricites*	2151	86	0	11.2	18.5	8.3	3.8	4.5	9.3	9.2
*Gardineroseris* sp. ^*(d)*^	78	46	0	4	43.3	8.5	4.5	4	39.3	4
*G. planulata (e)*	52	46	0	4	46	7	3	4	42	4
*Leptoseris* sp.	357	260	0	10	65	46	36	10	55	10
*Pavona* sp.	4331	1416	0	3	52	43	40	3	49	3
*Balanophyllia elegans*	398	1050	8	237.5	511.3	291.6	113.3	178.3	284.5	226.8
*Diploastrea* sp.	326	16	0	6	14	5	2	3	8	6
*D. heliopora*	326	16	0	6	14	5	2	3	8	6
*Galaxea* sp.	1447	57	0	5	46	34	31	3	41	5
*G. fascicularis*	1001	52	0	6	46	35	31	4	40	6
*Turbinaria* sp.	1948	72	0	5	45	6	3	3	40	5
*Fungia* sp.	1863	2440	0	4	51	40	36	4	47	4
*Lobophyllia* sp.	1410	48	0	5	12	6	3	3	7	5
*L. corymbosa*	266	16	1	6	12.9	5	2	3	8.9	4
*Symphyllia* sp.	891	50	0	5	43	31	28	3	38	5
*Cyphastrea* sp.	4154	98	0	5	53	40	35	5	48	5
*C. chalcidium*	191	15	0	5	13	5	2	3	8	5
*C. serailia*	417	24.9	0	5	12	6	3	3	7	5
*Echinopora* sp.	901	52	0	5	15	6	3	3	10	5
*E. gemmacea*	193	44	2	6	12	7.6	3.6	4	8	4
*Favites* sp.	2530	69.4.0	0	4	13	6	4	2	9	4
*F. pallida*	617	67	0	5	40	7.8	4.8	3	35	5
*F. speciosa*	177	47	0	5	10.1	6	3	3	5.1	5
*F. stelligera*	822	65	0	0	46	42	42	0	46	0
*Goniastrea* sp.	3165	1013	0	5	46	36	33	3	41	5
*G. pectinata*	571	51	0	5	42	8	5	3	37	5
*G. retiformis*	796	51	0	7	46	39	34	5	39	7
*Hydnophora* sp.	1110	67	0	3	33	7	5	2	30	3
*H. exesa*	452	47	0	4	12	6	4	2	8	4
*H. micronos*	233	65	0	2	46.4	6	4	2	44.4	2
*Leptoria* sp.	404	54	0	4	45	6	4	2	41	4
*L. phrygia*	356	54	0	4	10	6	4	2	6	4
*Orbicella annularis (M. annularis)*	6991	68	0	9.7	15.9	8.7	3	5.7	7.3	8.6
*Orbicella faveolata (M. faveolata)*	5073	65	0	10	18.7	9	5	4	10	8.7
*Oulophyllia* sp.	347	57	0	5	38	5	3	2	35	3
*Oulophyllia crispa*	259	40	0	5	11	5	3	2	8	3
*Platygyra* sp.	2211	50	0	5	40	6	3	3	35	5
*P. lamellina*	358	47	0	5	10.6	6	3	3	5.6	5
*P. sinensis*	342	48	0	5	13	6	3	3	8	5
*Montastraea* sp.	19323	85	0	10	38	9.9	4.9	5	29	9
*Montastrea cavernosa*	3552	85	0	10	21.5	8.5	4.3	4.2	13	8.5
*Colpophyllia* sp.	1772	81	0	9	20	8.8	4.6	4.2	13	7
*C. natans*	1772	81	0	9	20	8.8	4.6	4.2	13	7
*Diploria* sp.	4930	81	0	6.9	22.3	11.5	6.1	5.4	16	6.3
*Diploria labyrinthiformis*	978	81	0	9.7	17.8	10.2	4	6.2	9.3	8.5
*Pseudodiploria clivosa (D. clivosa)*	922	66	0	3.5	15.2	8	5.5	2.5	12.1	3.1
*Pseudodiploria strigosa (D. strigosa)*	3013	81	0	7	25.3	11.6	6	5.6	19	6.3
*Favia* sp.	5199	1013	0	5	45	35	31	4	40	5
*F. fragum* ^*(c)*^	183	80.5	0	9	60.7	30.9	23.9	7	52.4	8.3
*Mussidae (Faviidae)*	12746	1416	0	6	42	11	7	4	36	6
*Oculina* sp. *(d)*	83	1050	1.8	70	717.5	118.4	58.9	59.5	652	65.5
*Pocillopora* sp.	16430	98	0	0	57	43	43	0	57	0
*P. eydouxi*	710	65	0	0	48	8	8	0	48	0
*P meandrina*	8513	98	0	0	55	43	43	0	55	0
*P verrucosa*	799	54	0	0	44.5	7	7	0	44.5	0
*Stylophora* sp.	891	50	0	5	43	31	28	3	38	5
*Poritidae (family)*	56039	98	0	3.5	66	44	40.5	3.5	62.5	3.5
*Goniopora* sp.	1382	69.7	0	5	41	6	3	3	37	4
*G. lobata* ^*(e)*^	29	12	2	5	10	4.7	1.7	3	7	3
*Porites* sp.	54657	98	0	3.2	66	44	40.8	3.2	62.8	3.2
*P. asteroides*	6781	84	0	6.1	19.3	10.5	6.1	4.4	13.9	5.4
*P. brighami*	427	87	0	17	87	75	58	17	70	17
*P. cylindrica*	317	17.1	0	6	14	6	3	3	9	5
*P. lobata*	27402	98	0	0	66	45	45	0	66	0
*P. lutea*	1472	52	0	0	44	0	0	0	44	0
*P. nigrescens* ^*(d)*^	140	26.5	0	5	11	5.6	3.1	2.6	7	4
*P solida* ^*(c)*^	149	87	0	0	47	39.5	39.5	0	47	0
*Leptastrea* sp.	2218	86	0	5	46	40	35	5	41	5
*Pachyseris* sp.	621	60	0	6	12	7	4	3	8	4
*Plesiastrea* sp.	164	70	1	5	41.6	6	3	3	38.6	3
*Siderastrea* sp.	4742	85	0	9.8	29.9	11.5	4.2	7.3	21.2	8.7
*S. radians*	325	79	0	9.5	61.8	26.3	19.1	7.2	52.9	8.8
*S. siderea*	4397	85	0	9.8	21.5	11.2	3.9	7.3	12.8	8.7
*Millepora* sp.	3817	92	0	3	39.8	10.3	8.3	2	37	2.8
The modern depth distributions were determined using observations and live collected samples with a vertical precision ≤ 0.25 m (unless otherwise specified) from the OBIS database (Data Citation 2). a) depth precision ≤ 5 m; (b) depth precision ≤ 0.5 m; (c) depth precision ≤ 2 m; (d) depth precision ≤ 5 m using all data in the OBIS database; (e) too few observations, use the genus relationship.										

**Table 6:** Summary statistics for the basin, sub-basin and regional empirically derived, modern depth distributions of selected coral species

**Taxa**	**Basin**	**sub-basin**	**Number of obs.**	**Max depth (m)**	**Min depth (m)**	**Median depth**	**depth range (m) (95%)**	**depth range (m) (68%)**	**Lower error (68%)**	**Upper error (68%)**	**Lower error (95%)**	**Upper error (95%)**
*Acropora cervicornis*	GLOBAL	n/a	893	48	0.2	4.6	15.6	8.1	5.4	2.7	11.5	4.1
	Caribbean	n/a	893	48	0.2	4.6	15.6	8.1	5.4	2.7	11.5	4.1
	Caribbean	Belize	91	18.2	0.4	6.7	15.9	9.9	5.7	4.2	9.7	6.2
*Acropora palmata*	GLOBAL	n/a	4884	22	0	1.5	8.9	2.3	1.5	0.8	7.8	1.2
	Caribbean	n/a	4884	22	0	1.5	8.9	2.3	1.5	0.8	7.8	1.2
	Caribbean	Belize	377	16.4	0.4	2	10.7	7.5	6.5	1	9.1	1.6
	Caribbean	Jamaica	108	9.7	0.9	1.5	8.5	6.3	6.3	0	7.9	0.6
	Caribbean	Puerto Rico & Virgin Is.	88	22	2	3.5	12.2	3.2	2.2	1	10.7	1.5
	Caribbean	Bahamas &Turks & Caicos	1927	19	0.1	1.5	5.2	2.1	1.2	0.9	4	1.2
	Caribbean	Florida	150	8.3	2.3	2.8	3.5	1.7	1.4	0.3	3	0.5
	Caribbean	Panama	248	7.3	0.7	1.5	2.1	0.9	0.5	0.4	1.5	0.6
*Diploria* sp.	GLOBAL	n/a	4930	81	0	6.9	22.3	11.5	6.1	5.4	16	6.3
	Caribbean	n/a	4930	81	0	6.9	22.3	11.5	6.1	5.4	16	6.3
	Caribbean	Puerto Rico & Virgin Is.	345	81	0	11.5	64.9	34	27.6	6.5	54.6	10.3
	Caribbean	Florida	254	17	1	4.5	8.7	5.7	3.5	2.2	5.5	3.2
*Pseudodiploria strigosa*	GLOBAL	n/a	3013	81	0	7	25.3	11.6	6	5.6	19	6.3
*(Diploria strigosa)*	Caribbean	n/a	3013	81	0	7	25.3	11.6	6	5.6	19	6.3
	Caribbean	Belize	385	18.2	0.4	8.5	14.7	7.4	3	4.4	7.4	7.3
	Caribbean	Puerto Rico & Virgin Is.	221	81	0	11.75	66	35.2	28.4	6.8	55.2	10.7
	Caribbean	Florida	85	17	1	5.7	10.6	5.6	2.4	3.2	6.1	4.5
*Millepora* sp.	GLOBAL	n/a	3817	92	0	3	39.8	10.3	8.3	2	37	2.8
	Caribbean	n/a	3414	92	0	2.7	33.8	9	7.3	1.7	31.5	2.3
	Caribbean	Bahamas &Turks & Caicos	584	23	0.25	4	18.5	11.7	9	2.7	15	3.5
*Orbicella annularis*	GLOBAL	n/a	6991	68	0	9.7	15.9	8.7	3	5.7	7.3	8.6
*(Montastraea annularis )*	Caribbean	n/a	6991	68	0	9.7	15.9	8.7	3	5.7	7.3	8.6
	Caribbean	Belize	788	18.3	0.4	9.3	14.4	9.9	2.4	7.5	6	8.4
	Caribbean	Puerto Rico & Virgin Is.	808	78.5	2	7.8	13	8	4.3	3.8	7.8	5.2
	Caribbean	Florida	204	25.5	1	1.7	9.3	2.2	1.7	0.5	8.6	0.7
*Montestraea cavernosa*	GLOBAL	n/a	3552	85	0	10	21.5	8.5	4.3	4.2	13	8.47
	Caribbean	n/a	5411	320	0	11	73.6	26.5	22.5	4	65.1	8.5
	Caribbean	Florida	491	21	0.95	3.5	12.4	6.3	4.8	1.5	10.1	2.3
*M. faveolata*	GLOBAL	n/a	5073	65	0	10	18.7	9	5	4	10	8.7
	Caribbean	n/a	6266	111	0	10	19.5	8.8	4.8	4	11	8.5
	Caribbean	Puerto Rico & Virgin Is.	933	39.5	2.5	11.6	17.2	8.5	3.6	4.9	8.8	8.4
*Pavona* sp.	GLOBAL	n/a	4331	1416	0	3	52	43	40	3	49	3
	Pacific	n/a	4330	1416	0	3	52	43	40	3	49	3
	Pacific	Great Barrier Reef	641	1416	1	5	10.5	6	3	3	7	3.5
*Porites asteroides*	GLOBAL	n/a	6781	84	0	6.1	19.3	10.5	6.1	4.35	13.9	5.4
	Caribbean	n/a	6779	84	0	6.1	19.3	10.5	6.1	4.4	13.9	5.4
	Caribbean	Belize	603	18.2	0.3	6.1	14.9	9.6	6	3.6	10	4.9
	Caribbean	Bahamas &Turks & Caicos	773	15	0.4	5	14	7.2	4.7	2.5	10	4
*Porites cylindica*	GLOBAL	n/a	317	17.1	0	6	14	6	3	3	9	5
	Pacific	n/a	317	17.1	0	6	14	6	3	3	9	5
	Pacific	Great Barrier Reef	310	17.1	1	6	13	6.9	3.9	3	9	4
*Porites* sp.	GLOBAL	n/a	54657	98	0	3.2	66	44	40.8	3.2	62.8	3.2
	Pacific	n/a	44553	98	0	0	67	45	45	0	67	0
	Pacific	Great Barrier Reef	1999	64.6	0	5	12	6	3	3	8	4
	Caribbean	n/a	10101	85	0	7	19.2	10.4	5.3	5.1	13	6.2
	Caribbean	Belize	799	18.2	0.3	7	15.4	9.7	5.1	4.6	9.4	6
*Siderastrea siderea*	GLOBAL	n/a	4397	85	0	9.8	21.5	11.2	3.9	7.3	12.8	8.7
	Caribbean	n/a	4377	85	0	9.9	21.9	11.2	3.9	7.4	13.2	8.8
	Caribbean	Belize	397	17	1	9.7	14.5	8	3.3	4.7	6.3	8.2
	Caribbean	Puerto Rico & Virgin Is.	399	85	0	10	64.7	26.3	22	4.3	57.5	7.1
Data are a subset of that detailed in Table 5 (Data Citation 2).												

**Table 7:** Descriptions of the fields used in the database

**Category**	**Column**	**Type**	**Column Name**	**Units**	**Description**
Sample ID	A	identifier	ID	n/a	Database entry number
	B	identifier	Source	n/a	Publication source (for full details see ‘Sources’)
	C	identifier	Analysis ID	n/a	Published sample identifier (note, for radiocarbon dated samples, we use the radiocarbon publication code in preference to any other sample identifier)
Location information	D	data	Location	n/a	Geographic location
	E	data	Site	n/a	Local Site Name
	F	data	Additional Locality Info	n/a	More detailed site descriptors
	G	screening	location tag	n/a	numerical location identifier
	H	data	Latitude	degrees	latitude (°N or S)
	I	data	Longitude	degrees	longitude (°E or W)
	J	data	Decimal latitude	degrees	latitude (+ve=N; -ve =S)
	K	data	Decimal longitude	degrees	longitude (+ve=E; -ve =W)
	L	comment	lat/long estimated?	n/a	whether the latitude and longitude estimated
Tectonic information	M	data	Tectonic Category	n/a	Teconic setting—is the site uplifting, subsiding, or stable
	N	interpretation	Uplift reported in original references	m/ka	Rate of uplift or subsidence given in the original publication
	O	interpretation	Uplift error reported in original references	m/ka	Uplift or subsidence rate error given in the original publication
	P	interpretation	Uplift rate used	m/ka	Uplift or subsidence rate used in the calculation of Z_cp_ and P_RSL_
	Q	interpretation	Uplift rate error used	m/ka	Uplift or subsidence rate error used in the calculation of Z_cp_ and P_RSL_
	R	comment	Comments (uplift)	n/a	Comments
Elevation information	S	data	setting: outcrop/drill core etc.?	n/a	Details of the geological setting, for example, was the sample from a drill core or outcrop/exposure
	T	data	modern setting	n/a	Details of the modern setting of the sample/core etc.
	U	data	core ID	n/a	The core identifier (where reported)
	V	data	Original elevation datum used	n/a	The elevation datum used in the original publication
	W	data	How elevation derived	n/a	The method used to obtain the sample elevation (e.g., surveying, GPS, etc.)
	X	data	reported uncert.	m	The elevation uncertainty associated with establishing the core top/outcrop elevation. Derived from the method of establishing the elevation (e.g., levelling)
	Y	interpretation	allocated uncert.	m	For samples where no uncertainty is specified, the allocated uncertainty based on the method for establishing the elevation
	Z	comment	comment (elevation measurement)	n/a	Comments on how the elevation, or associated uncertainty was established
	AA	data	If core, coring method	n/a	Method used to aquire sample
	AB	data	If core, water depth OR core surface elevation	m	The elevation or water depth of the core top
	AC	data	reported uncert.	m	The uncertainty assocaited with sample acquisition, for example approximated amount of core stretching
	AD	interpretation	allocated uncert.	m	For samples where no uncertainty is specified in column AD, the allocated uncertainty based on the method for aquiring the sample/core etc.
	AE	comment	comment (coring)	n/a	Comments on sample acquisition
	AF	data	If core, depth in core	m	Depth/elevation of the sample within the core
	AG	data	reported uncert. (sampling uncert.)	m	The uncertainty assocaited with sampling
	AH	interpretation	allocated uncert.	m	For samples where no uncertainty is specified in column AH, the allocated uncertainty
	AI	data	Elevation reported in original reference	m	The elevation of the sample as reported in the original publication
	AJ	data	Elevation uncertainty reported in original reference	m	The elevation uncertainty of the sample as reported in the original publication
	AK	data	Elevation obtained from other references	m	The elevation of the sample obtained from other sources (if not given in the original publication)—see comments column
	AL	data	Elevation uncertainty obtained from other references	m	The elevation error of the sample obtained from other sources (if not given in the original publication)
	AM	comment	Comments (elevation)	n/a	Comments on sample elevation
	AN	data	Elevation used (m) referenced to MSL	m	The modern elevation of the sample relative to mean sea level used in the calculation of P_RSL_
	AO	data	Elevation uncertainty used (m)	m	The elevation error of the sample used in the calculation of P_RSL_
	AP	data	datum elevation referenced to	n/a	The datum the elevation used (column AO) is referenced to
	AQ	comment	Comments (elevation error)	n/a	Comments on elevation uncertainty
Stratigraphic information	AR	data	Stratigraphic constraint available	yes/no	If there is some stratigraphic context for samples, for example, is the same one from a coherent stratigrphic sequence such as a core, a drill core or a vertically coherent stratigraphic section or traverse?
	AS	data	Stratigraphic group	n/a	Numerical identifier for samples from the same stratigraphic sequence. X.1 is the top to X.n
Tidal information	AT	data	Reported modern tidal range	m	The reported modern tidal range
	AU	data	tidal range (m) from other sources OR comment	m	The tidal range derived from other sources OR a comment relating to the tidal range for the site
Corrected position (Z_cp_) calculation	AV	interpretation	Zcp	m	The corrected (for uplift or subsidence since the time of formation) sample elevation referenced to MSL
	AW	interpretation	Zcp uncertainty	m	2 sigma corrected sample elevation uncertainty
	AX	comment	comment (Zcp)	n/a	Comments relating to the corrected position (Zcp)
Sample information and context	AY	data	Material dated	n/a	Material dated
	AZ	data	Species dated (if given)	n/a	The species (or genus) of material dated (where reported)
	BA	interpretation	Facies	n/a	Sedimentary facies context and outcrop information on coral sample
	BB	data	If living, year of collection	Date	The year of collection for any live collected samples
	BC	data	Explicitly state reef crest/top targeted in original sampling?	yes/no	If the original publication explicitly stated that the reef crest (or close to) was targeted during sampling
	BD	interpretation	Terrace Identification	n/a	Identified terrace the sample came from
	BE	interpretation	Additional sample information	n/a	Any additional sample information
	BF	interpretation	In growth position?	yes/no	If the sample was explicitly stated as being in growth position in the original publication
	BG	interpretation	In situ?	yes/no	If the samples was explicitly stated as being *in situ* in the original publication. Note, for drill cores we have assumed samples are **no**t *in situ* unless explicitly stated
	BH	interpretation	*In situ/in growth*? (strict)	yes/no	If the sample is in situ OR in growth position (explicitly stated in the original publication)
CORAL species information	BI	data	Species dated	n/a	Taxonomic identification of coral sample
	BJ	interpretation	growth form	n/a	Growth form of coral
	BK	comment	Comments (species)	n/a	Comments
	BL	screening	Taxa tag	n/a	Numerical taxa identifier (as per Hibbert et al., 2016)
	BM	screening	UPDATED taxa tag	n/a	Numerical taxa identifier, updated Jan 2017 (this study) using OBIS extracts
	BN	screening	REGIONAL taxa tag	n/a	Numerical taxa identifier, using updated Jan 2017 (this study) OBIS extracts
	BO	interpretation	Updated GLOBAL median depth (m)	m	The median habitat-depth (determined from our synthesis of modern ecological data) for the taxa dated (negative values are m below sea level). Note these have been updated from Hibbert et al., 2016 to include new data added by OBIS
	BP	interpretation	Updated GLOBAL lower depth (95%)	m	The lower taxon specific depth (95% limits) derived from modern synthesis. Note, this is a stochastic component of vertical uncertainty
	BQ	interpretation	Updated GLOBAL upper depth (95%)	m	The upper taxon specific depth (95% limits) derived from modern synthesis. Note, this is a stochastic component of vertical uncertainty
	BR	interpretation	Updated GLOBAL lower depth (68%)	m	The lower taxon specific depth (68% limits) derived from modern synthesis. Note, this is a stochastic component of vertical uncertainty
	BS	interpretation	Updated GLOBAL upper depth (68%)	m	The upper taxon specific depth (68% limits) derived from modern synthesis. Note, this is a stochastic component of vertical uncertainty
	BT	data	modern depth distribution (assemblage or other) study undertaken?	yes/no	Was a modern coral/coralgal etc. study undertaken?
	BU	data	type (coral, coralgal, foram, modern depth distribution etc.)	n/a	What type of assemblage study; coral, coralgal etc.
	BV	data	scale (site, local, regional)	n/a	At what scale was the assemblage study undertaken; site specific, local, regional
	BW	interpretation	given assemblage (as originally reported)	n/a	The assemblage the dated sample is from
	BX	data	Fossil assemblage (or principal faunal composition) undertaken?	yes/no	Was a study of the fossil coral assemblage undertaken (i.e., full assemblage or main species composition)?
	BY	interpretation	original palaeodepth interpretation	n/a	The environmental or palaeo water-depth interpretation from the original publication (or references therein)
	BZ	interpretation	Used in original palaeo sea level reconstruction (or error)?	yes/no/error	Was the assemblage information used in the calculation of sea level (or the error) in the original publication?
	CA	interpretation	Reported upper limit of palaeo-water depth (m)	m	Upper limit of the coral species/palaeo-water depth range given by the original authors
	CB	interpretation	Reported lower limit of palaeo-water depth (m)	m	Lower limit of the coral species/palaeo-water depth range given by the original authors
	CC	interpretation	original authors plot as	n/a	How the vertical uncertainties relating to the depth habitat of the coral speacies was plotted in the original publication
	CD	comment	comment (palaeo water depth)	n/a	Comment/clarification on how the uncertainty derived from the species depth habitat was originally plotted
Facies formation information	CE	interpretation	facies information	n/a	Sedimentary facies context and/or exposure information
	CF	interpretation	unit	n/a	Sedimentary unit the sample was obtained from
	CG	interpretation	infered depositional environment	n/a	The infered depositional environment
	CH	interpretation	other sample information	n/a	Any additional sample information
	CI	interpretation	reported formation range	m	The environmental range over which the sample type forms, i.e., the formation range from the original publication (or references therein)
	CJ	interpretation	error type	n/a	The uncertainty distribution of sample: coral-type, range, limiting etc.
	CK	interpretation	Upper limit of formation range	m	The upper limit of the formation range given by the original authors; +ve is above MSL, -ve is below MSL
	CL	interpretation	Lower limit of formation range	m	The lower limit of the formation range given by the original authors; +ve is above MSL, -ve is below MSL
	CM	data	How obtained	n/a	The derivation of the formation range given, e.g., using a modern analogue
	CN	data	Scale (site, local, regional)	n/a	The scale the formation range study undertaken; site specific, local, regional
	CO	data	type	n/a	The type of study undertaken by the original publication (or references therein) to derive the formation depth range
	CP	data	datum	n/a	The datum to which the formation depth range if referenced
	CQ	data	Reference for formation range	n/a	Publication in which study of formation depth range is detailed
	CR	comment	Comments (facies formation range)	n/a	Comment on the formation depth range
Dating information, GENERAL	CS	data	Replicate dating?	yes/no	Yes or no indicates whether measurement is a replicate
	CT	comment	Comments (replicate)	n/a	Comments on the type of replicate, e.g., are the replicates from the same aliquot, same slice, same coral head etc.
	CU	data	Replicate Group	n/a	Numerical identifier associating replicate dated samples
	CV	data	Dating Method	n/a	Method used to date the sample
	CW	data	If U/Th, is there a ‘paired’ 231Pa/235U age?	yes/no	If the dating was carried out using U-series analysis, is there also a ^231^Pa/^235^U age determination on the same sample?
	CX	data	If U/Th, is there also a 14C age?	yes/no	If the dating was carried out using U-series analysis, is there also a ^14^C age determination on the same sample?
	CY	interpretation	Recalculated Age (U-series and 14C) (ka BP)	ka BP	Recalculated age, in ka BP
	CZ	interpretation	uncert. (ka BP ±2σ)	ka BP	Recalculated age uncertainty, in ka BP
U-series dating information	DA	data	Instrument	n/a	Type of mass spectrometer: TIMS or MC-ICP-MS
	DB	interpretation	year measured	n/a	The year the sample was U-series dated
	DC	interpretation	year estimated	yes/no	Was the year of measurement estimated
	DD	data	Decay constants	D1/D2/D3	D1=^234^U decay constant from Holden (1989^327^) and ^230^Th decay cnst. of Meadows et al (1980^328^); D2=^234^U and ^230^Th decay cnst. from Cheng et al. (2000^31^); D3=^234^U and ^230^Th decay cnst. from Cheng et al. (2013^29^); If no information available, assumed SE and indicated in column DL
	DE	comment	comments (decay constant)	n/a	Comments relating to the decay constants used in the original publication
	DF	data	Spike calibration	G/SE/SE?/(G/SE)	Type of standard used for spike calibration: G=gravimetric or SE=secular equilibrium, SE?=assumed secular equilibrium but unable to confirm G/SE=Uranium isotopes gravimetrically calibrated, Thorium isotopes calibrated to secular equilibrium standard ; If no information available, assumed SE and indicated in column DN; here no information was available have assumed SE
	DG	comment	Comments (spike calibration)	n/a	Comments relating to the spike used in the original analysis
	DH	data	% calcite	%	% calcite content of the sample
	DI	data	determined by	n/a	How the % calcite was determined e.g., petrographic methods, XRD etc.
	DJ	screening	% calcite ≤ 1 %	yes/no	% calcite ≤ 1 %
	DK	screening	% calcite ≤ 2 %	yes/no	% calcite ≤ 2 %
	DL	screening	% calcite ≤ 5 %	yes/no	% calcite ≤ 5 %
	DM	data	[^232^Th ]	ppb	The concentration of ^232^Th
	DN	data	uncert. (±2σ)	ppb	The uncertainty in the ^232^Th concentration (2 σ)
	DO	screening	^[232^Th ] ≤ 1 ppb	yes/no	^[232^Th ] ≤ 1 ppb
	DP	screening	^[232^Th ] ≤ 2 ppb	yes/no	^[232^Th ] ≤ 2 ppb
	DQ	data	[^230^Th/ ^232^Th]ACT	n/a	^230^Th/^232^Th activity ratio, if included in source data table
	DR	data	[^230^Th/^232^Th]ACT uncert. (±2σ)	n/a	Uncertainty for ^230^Th/^233^Th activity ratio
	DS	comment	[^230^Th/ ^232^Th]ACT back-calculated?	yes/no	Was the ^230^Th/^232^Th activity ratio back-calculated?
	DT	screening	[^230^Th/ ^232^Th]ACT >20?	yes/no	Is the [^230^Th/ ^232^Th]ACT >20?
	DU	data	(^232^Th /^238^U)*10^5	n/a	
	DV	data	[^238^U]	ppm	^238^U concentration
	DW	data	uncert. (±2σ)	ppm	The uncertainty associated with the ^238^U concentration (2 σ)
	DX	data	Reported [^230^Th/^234^U]ACT	n/a	The ^230^Th/^234^U activity ratio, if included in source data table
	DY	data	uncert. (±2σ)	n/a	Uncertainty for ^230^Th/^234^U activity ratio
	DZ	data	Reported [^230^Th/^238^U]ACT	n/a	The measured activity ratio of ^230^Th/^238^U as originally reported; back-calculated values are indicated in column EB
	EA	data	uncert. (±2σ)	n/a	The 2 sigma error on measured activity ratio of ^230^Th/^238^U as originally reported; back-calculated values are indicated in column EB
	EB	data	[^230^Th/^238^U]ACT back-calculated?	yes/no	Was the ^230^Th/^238^U activity ratio back-calculated?
	EC	data	Reported [^234^U/^238^U]ACT	n/a	The measured activity ratio of ^234^U/^238^U as originally reported; back-calculated values indicated in column EE
	ED	data	uncert. (±2σ)	n/a	The 2 sigma error on measured activity ratio of ^234^U/^238^U as originally reported; back-calculated values indicated in column EE
	EE	comment	[^234^U/^238^U]ACT back-calculated?	yes/no	Was the ^234^U/^238^U activity ratio back-calculated?
	EF	interpretation	Reported age (ka)	ka	Calculated age, as originally reported
	EG	interpretation	uncert. (±2σ)	ka	The uncertainty associated with the reported age (±2 σ)
	EH	interpretation	Reported age (ka) Th corrected	ka	The calculated age, including detrial thorium correction, as originally reported
	EI	interpretation	uncert. (±2σ)	ka	The uncertainty (±2 σ) associated with the age, corrected for detrital thorium, as originally reported
	EJ	data	detrital Th correction applied	n/a	Further details of any detrital thorium correction applied by the original authors
	EK	comment	comment (Reported age)	n/a	Comment on the reported U-series age
	EL	interpretation	Reported d^234^U _initial_ (‰)	per mille (‰)	Calculated δ^234^U_initial_ as originally reported
	EM	interpretation	uncert. (±2σ)	per mille (‰)	The uncertainty (±2 σ) associated with the δ^234^U initial, as originally reported
	EN	data	Recalculated [^230^Th/^238^U]ACT	n/a	The measured activity ratio of ^230^Th/^238^U re-calculated for data using a gravimetrically-calibrated spike using the Cheng et al (2013) half-lives for ^230^Th
	EO	data	uncert. (±2σ)	n/a	The 2 sigma error on measured activity ratio of ^230^Th/^238^U as originally reported
	EP	data	Recalculated [^234^U/^238^U]ACT	n/a	The measured activity ratio of ^234^U/^238^U re-calculated for data derived with a gravimetrically-calibrated spike using the Cheng et al (2013) half-life for ^234^U
	EQ	data	uncert. (±2σ)	n/a	The 2 sigma error on measured activity ratio of ^234^U/^238^U as originally reported
	ER	interpretation	Recalculated Conventional Age (ka)	ka	Age calculated iteratively using Eqn. 1
	ES	interpretation	Recalculated Conventional Age referenced to ka BP	ka BP	Recalculated age (ka) referenced to AD 1950 (i.e., reported as ka BP)
	ET	comment	Comment (Age and δ^234^U_initial_)	n/a	Comments on age and δ^234^U_initial_ recalculation
	EU	interpretation	Recalculated Conventional Age uncert. (±2σ)	ka	2 sigma error on age; does not include uncertainty in decay constants
	EV	interpretation	Recalculated Conventional Age uncert. (±1σ)	ka	1 sigma error on age; does not include uncertainty in decay constants
	EW	interpretation	Recalculated Conventional Age uncert. (±2σ) includes decay cnst. uncert.	ka	2 sigma error on age; decay constant uncertainties included
	EX	interpretation	Recalculated Conventional Age uncert. (±1σ) includes decay cnst. uncert.	ka	1 sigma error on age; decay constant uncertainties included;
	EY	interpretation	Recalculated δ^234^U_initial_ (‰)	per mille (‰)	Initial δ^234^U value calculated using Eqn. 2
	EZ	interpretation	Recalculated ẟ234U initial uncert. (±2σ)	per mille (‰)	2 sigma error of the calculated δ^234^U_initial_ value; does not include decay constant uncertainty
	FA	interpretation	Recalculated ẟ234U initial uncert. (±2σ) (inc. decay cnst. uncert.)	per mille (‰)	2 sigma error of the calculated δ^234^U_initial_ value; decay constant uncertainties included
	FB	screening	δ^234^Ui screening criteria	interglacial, glacial, >130 ka	screening
	FC	screening	δ^234^Ui screening criteria (interglacial 147±5 per mille)	yes/no	screening
	FD	screening	δ^234^Ui screening criteria (interglacial 147±7 per mille)	yes/no	screening
Radiocarbon dating information	FE	data	^14^C publication code	n/a	Publication code of sample (as given by the lab)
	FF	data	Lab	n/a	Laboratory where analysis was undertaken
	FG	data	instrument	n/a	Instrument
	FH	data	material dated	n/a	Detailed information on the material submitted for radiocardon dating
	FI	data	Pre-treatment	n/a	Details of any pre-treatment carried out prior to radiocarbon anlysis of the sample
	FJ	data	sample carbon content (yield) (% by weight)	%	Sample carbon content (yield) (% by weight)
	FK	data	δ^13^C (‰ VPDB)	per mille (‰)	The δ^13^C value of the sample
	FL	data	uncert. (±1σ)	per mille (‰)	Uncertainty associated with the sample δ^13^C measurement
	FM	comment	comment (δ^13^C)	n/a	Comment regarding the δ^13^C meaurement of the sample, e.g., if the analysis was carried out offline etc.
	FN	data	δ^13^C normalisation undertaken (yes/no/assumed yes (even if no d13C given)	yes/no/assumed yes	Was the sample δ^13^C corrected by the lab?
	FO	data	^14^C enrichment (% modern carbon)	% modern carbon	The reported ^14^C enrichment (% modern carbon)
	FP	data	uncert. (±1σ)	% modern carbon	The uncertainty (±1σ) associated with the measured ^14^C enrichment
	FQ	data	F^14^C	dimensionless	Measured fraction modern carbon of sample
	FR	data	uncert. (±2σ)	dimensionless	The uncertainty (±2σ) associated with the measured fraction modern of the sample
	FS	data	background correction applied?	yes/no/not reported	Was the sample background corrected by the lab?
	FT	data	How background correction determined	n/a	Further details on how the background correction was derived
	FU	data	value (%MC)	% modern carbon	The value of the background correction applied (% modern carbon)
	FV	data	A0 (%MC)	% modern carbon	Background enrichment (% modern carbon)
	FW	comment	comment (background)	n/a	Comment on the background correction applied by the lab
	FX	data	Reported ^14^C age (yr BP) (if not conventional)	year	Reported ^14^C date where the determination is non-conventional
	FY	data	uncert. (±1σ)	year	The uncertainty (±1σ) associated with the non-conventional ^14^C date
	FZ	data	^14^C/^12^C or ^14^C/^13^C ratio used for retrospective δ^13^C correction?	n/a	Was the measurement for the retrospective δ^13^C correction a ^14^C/^12^C or ^14^C/^13^C measurement? Where this is not given in the original publication, an assumed measurement type used based on the instrument and lab
	GA	data	δ^13^C correction applied (‰)	per mille (‰)	The value of δ^13^C correction retrospectively applied to recalculate a conventional ^14^C date
	GB	data	uncert. (±1σ)	per mille (‰)	The uncertainty (±1σ) associated with the estimated δ^13^C correction
	GC	comment	Comments (δ^13^C correction)	n/a	Comments on the δ^13^C correction applied to non-conventional radiocarbon dates
	GD	interpretation	Conventional ^14^C age (yr BP)	year	Reported (or recalculated) conventional ^14^C date of sample
	GE	interpretation	uncert. (±1σ)	year	Uncertainty associated with the reported (or recalculated ) conventional ^14^C date
	GF	data	calibration curve used in original publication	n/a	The calibration dataset used in the original publication
	GG	data	calibration programme used in original publication	n/a	The calibration programme (and version) used in the original publication
	GH	interpretation	ΔR (years) applied	years	For marine samples, the regional marine reservoir correction (ΔR) applied in the original publication
	GI	interpretation	uncert. (±1σ)	years	Uncertainty associated with the regional marine reservoir correction (ΔR) applied in the original publication
	GJ	data	reference for ΔR used	n/a	Reference for the regional marine reservoir correction (ΔR) applied in the original publication
	GK	interpretation	Reported calibrated age range (yrs; 95% confidence interval) originally reported (yrs BP)	years (BP)	The reported calibrated age range at the 95 % confidence interval, as reported in the original publication
	GL	interpretation	Reported calibrated age range (yrs; 68% confidence interval) originally reported (yrs BP)	years (BP)	The reported calibrated age range at the 68 % confidence interval, as reported in the original publication
	GM	interpretation	Reported age (yr)	years (BP)	The reported, calibrated age (years BP)
	GN	interpretation	uncert. (±1σ)	years (BP)	The reported, calibrated age uncertainty (years)
	GO	data	calibration curve used in recalibration	n/a	The calibration dataset used in the recalibration of the age (this study)
	GP	data	calibration programme used in recalibration	n/a	The calibration programme (and version) used in recalibration of the sample ^14^C date (this study)
	GQ	interpretation	ΔR (years) applied in recalibration	years	The regional marine reservoir correction (ΔR) applied in the recalibration of the sample ^14^C date (this study)
	GR	interpretation	uncert. (±1σ)	years	The uncertainty associated with the regional marine reservoir correction (ΔR) applied in the recalibration of the sample ^14^C date (this study)
	GS	data	reference for ΔR used in recalibration	n/a	Reference for the regional marine reservoir correction (ΔR) applied in the recalibration of the sample ^14^C date (this study)
	GT	interpretation	Recalculated unmodelled calibrated upper age limit (yrs; 68% confidence interval)	years (BP)	The upper calibrated age limit at the 68% confidence interval of the recalibrated date (this study)
	GU	interpretation	Recalculated unmodelled calibrated lower age limit (yrs; 68% confidence interval)	years (BP)	The lower calibrated age limit at the 68% confidence interval of the recalibrated date (this study)
	GV	interpretation	Recalculated unmodelled calibrated upper age limit (yrs; 95% confidence interval)	years (BP)	The upper calibrated age limit at the 95% confidence interval of the recalibrated date (this study)
	GW	interpretation	Recalculated unmodelled calibrated lower age limit (yrs; 95% confidence interval)	years (BP)	The lower calibrated age limit at the 95% confidence interval of the recalibrated date (this study)
	GX	interpretation	Recalculated (un-modelled) μ (ka BP)	ka (BP)	The recalibrated mean age, in kilo-years BP (this study) referenced to AD 1950
	GY	interpretation	Recalculated (un-modelled) uncertainty (±1σ)	ka (BP)	The recalibrated age uncertainty (ka) (this study)
AAR dating information	GZ	data	AAR Publication Code	n/a	Publication code of sample (as given by the lab)
	HA	data	Lab	n/a	Laboratory where analysis was undertaken
	HB	data	Material dated	n/a	Detailed information on the material submitted for dating
	HC	data	Racemisation ratio based on?	n/a	Analytical details
	HD	data	Ratio		Further analytical details
	HE	interpretation	Reported Age (ka)	ka	The reported age
	HF	interpretation	reported uncertainty (±2 σ)	ka	The reported age uncertainty
OSL dating information	HG	data	OSL Publication code	n/a	Publication code of sample (as given by the lab)
	HH	data	Lab	n/a	Laboratory where analysis was undertaken
	HI	data	Material dated	n/a	Detailed information on the material submitted for dating
	HJ	data	protocol	n/a	Analytical details
	HK	interpretation	Reported Age (ka)	ka	The reported age
	HL	interpretation	reported uncertainty (±2σ)	ka	The reported age uncertainty
P_RSL_ calculation (using probability depth distributions)	HM	interpretation	Median sampled age (ka BP)	ka BP	The calculated median age (given the age probability distribution) for the calculated P_RSL_
	HN	interpretation	median P_RSL_	m	Median probability distribution of relative sea level (note, that for coral samples, P_RSL_=Zcp as the corrected position is assumed to be at the median depth based on modern taxonomic depth distributions)
	HO	interpretation	3σ-like boundaries of P_RSL_	m	Lower 3 sigma-like boundary of P_RSL_
	HP	interpretation	3σ-like boundaries of P_RSL_	m	Upper 3 sigma-like boundary of P_RSL_
	HQ	interpretation	2σ-like boundaries of P_RSL_	m	Lower 2 sigma-like boundary of P_RSL_
	HR	interpretation	2σ-like boundaries of P_RSL_	m	Upper 2 sigma-like boundary of P_RSL_
	HS	interpretation	1σ-like boundaries of P_RSL_	m	Lower 1 sigma-like boundary of P_RSL_
	HT	interpretation	1σ-like boundaries of P_RSL_	m	Upper 1 sigma-like boundary of P_RSL_
	HU	interpretation	−3σ-like uncertainty	m	approximation of the negative 3σ P_RSL_ error bar
	HV	interpretation	+3σ-like uncertainty	m	approximation of the positive 3σ P_RSL_ error bar
	HW	interpretation	−2σ-like uncertainty	m	approximation of the negative 2σ P_RSL_ error bar
	HX	interpretation	+2σ-like uncertainty	m	approximation of the positive 2σ P_RSL_ error bar
	HY	interpretation	−1σ-like uncertainty	m	approximation of the negative 1σ P_RSL_ error bar
	HZ	interpretation	+1σ-like uncertainty	m	approximation of the positive 1σ P_RSL_ error bar
Limiting data: limit of RSL	IA	interpretation	LIMITING data: UPPER limit RSL (m)	m	The upper limit of former sea levels for the given limiting data (i.e., sea level is somewhere BELOW this elevation)
	IB	interpretation	LIMITING data: LOWER limit RSL (m)	m	The lower limit of former sea levels for the given limiting data (i.e., sea level is somewhere ABOVE this elevation)
DATA QUALITY CONTROL	IC	screening	Reject (age)?	n/a	Reject the age of the sample?
	ID	screening	reason for rejection	n/a	Reason for the rejection of the age (and if rejected by author)
	IE	screening	Reject PRSL	n/a	Reject the reconstructed PRSL of the sample?
	IF	screening	Reason for rejection	n/a	Reason for the rejection of the age (and if rejected by author)
