# Supplementary material for: A database of biological and geomorphological sea-level markers from the Last Glacial Maximum to present
Source: Sci Data. 2018 May 29;5:180088. doi: 10.1038/sdata.2018.88 (PMC5972710; doi:10.1038/sdata.2018.88)
Supplement: Supplementary Information [file sdata201888-s2.pdf]

Supplementary Information:

Hibbert et al., *A database of biological and geomorphological sea-level markers from the Last Glacial Maximum to the present*

Table of Contents:

Australia and New Zealand
Joseph Bonaparte Gulf and Northern Territory (NT)[1–6]
New South Wales (NSW)[7–12]
South Australia (SA)[13–18]
Victoria (VIC)[7,9,19]
Tasmania[7]
Queensland (QLD)[20–38] and Torres Strait[24]
New Zealand[39–53]

Pacific Ocean
Society, Tuamotu, Gambier, and Austral Islands[54–67]
Southern Cook Islands[68–71]

South America
Argentine Shelf[72]

Indian Ocean
Mayotte (Comoro Archipelago)[73–75]
Mauritius[73,76]
Maldives[77–80]
Reunion Island[73]
Zanzibar, Tanzania[81]

Indian Subcontinent
Bay of Bengal[82]
Bangladesh[83]
Sri Lanka[84]

Southern Africa
South Africa[85–95]
Mozambique[96,97]

S.E. Asia
Sunda Shelf [98,99]
Japan[100]
China[101–119]
Thailand[120–126]
Malaysia[121,127–129]
Singapore[130–132]
Vietnam[133] (and Vietnam Shelf[98])
Korea/Yellow Sea[107]

Caribbean
Barbados[134–137]
Jamaica[138,139]
Belize[140–151]
Florida[139,152–158]
Bahamas[154,159]
Martinique[154,160]
Panama[154,161]
Puerto Rico[154,162]
Antigua[163]
Grand Cayman[164]
U.S. Virgin Islands: St Croix[154,165–169]
Trinidad[170,171]
Bermuda[172]

Abbreviations used:

***Tidal parameters and datums*:**	
MSL	Mean sea level: mean value of sea level (from a suitably long time series)
MTL	Mean tide level: arithmetic mean of mean high water and mean low water (over a suitably long period)
HAT	Highest astronomical tide: highest tide level that can be predicted under average meteorological conditions
LAT	Lowest astronomical tide: lowest tide level that can be predicted under average meteorological conditions
MHW	Mean high water: the average of all high water heights observed over a period
MHWS	Mean high water springs: average of high water heights occurring at the time of the spring tides
MHWN	Mean high water neaps: average of the high water heights occurring at the time of neap tides
MLW	Mean low water: average of all the low water heights observed over a period
MLWS	Mean low water springs: average of low water heights occurring at the time of the spring tides
MLWN	Mean low water neaps: average of the low water heights occurring at the time of neap tides
MTR	Mean tidal range: the difference between MHW and MLW
AHD	Australian height datum (http://www.ga.gov.au/scientific-topics/positioning-navigation/geodesy/geodetic-datums/australian-height-datum-ahd)
HLC	Highest living coral (microatoll)
Sea level attributes	
RSL	Relative sea level
IR	Indicative range: the elevation range over which an indicator forms, relative to some datum
RWL	Reference water level: mid-point of the indicative range
Radiocarbon	
ΔR	regional marine reservoir age correction

AUSTRALIA AND NEW ZEALAND

For the recalibration of marine radiocarbon dates we use the appropriate regional ΔR corrections detailed below.

South Australia: 7 samples listed in the online database[173].

Location	ΔR (years)	± 1σ	source
Upper Spencer Gulf, SA	60	143	174
Upper Spencer Gulf, SA	109	124	174
Upper Spencer Gulf, SA	-12	183	174
Gulf St Vincent, SA	-12	84	174
Pondalowie Bay, SA	101	81	174
Pondalowie Bay, SA	21	81	174
Gulf St Vincent, Adelaide	137	86	175
**Weighted mean**	**62**		
**Standard deviation**	**61**		
**n**	**7**		

Victoria, Melbourne, Tasmania: influenced by surface flow through/within the Bass Strait. Only one sample in the online database[173].

Location	ΔR (years)	± 1σ	source
Key Island, Furneaux Group	-14	120	176
**Weighted mean**	**n/a**		
**Standard deviation**	**n/a**		
**n**	**1**		

New South Wales: one sample from Narooma[175]; the next closet samples are from Moreton Bay (nr. Brisbane) which are under the influence of the same surface currents, but potentially more variable ΔR to the south. Used the single Narooma determination[175] as recalculated in the online database[173] (ΔR = 11 ± 85 years).

Location	ΔR (years)	± 1σ	source
Narooma	11	85	175
**Weighted mean**	**n/a**		
**Standard deviation**	**n/a**		
**n**	**1**		

Queensland (open ocean, near shore settings): open ocean circulation dominated by the East Australian Current with samples from near Brisbane (Stradbroke Island conforming to other ΔR determinations from further north and the Torres Strait[177]) (note, that some sheltered bays and estuaries have very different residence times, hydrological inputs and geological settings, that may significantly alter the ΔR value from the open ocean[178]). Used all the available open ocean ΔR determinations from the Queensland coastline and Torres Strait[178]; ΔR = 11 ± 14 years (n=12).

Location	ΔR (years)	± 1σ	source
Elliott Heads	-51	60	178
Gladstone	30	50	178
Gladstone	-90	60	178
Port Curtis	7	60	178
Port Curtis	117	60	178
Stradbroke Island	26	23	177
Stradbroke Island	-9	23	177
Heron Island	8	6	179
Abraham Reef	15	6	179
Torres Strait	78	68	175
Torres Strait	61	85	175
Torres Strait	-5	84	175
**Weighted mean**	**11**		
**Standard deviation**	**14**		
**n**	**12**		

Joseph Bonaparte Gulf: wider regional surface circulation dominated by the Holloway Current and the South Equatorial Current. No determinations from the area but some analyses to both the north[180] (n=1) and to the south[174,181]. We calculate a weighted mean for these sites using the vales in the online database[173]; ΔR = 58 ± 22 (n=14).

Location	ΔR (years)	± 1σ	source
Raffles Bay, N. Australia	59	40	180
Roebuck Bay	67	35	181
Broome, WA	109	78	174
Broome, WA	15	78	174
Broome, WA	11	109	174
Broome, WA	7	119	174
Broome, WA	-41	119	174
Broome, WA	112	78	174
Broome, WA	67	30	181
Broome, WA	32	35	181
Gantheaume Point	62	30	181
Skeleton Point King Sound	50	30	181
Cape Leveque, NE side	82	30	181
Cape Leveque, NE side	42	30	181
**Weighted mean**	**58**		
**Standard deviation**	**22**		
**n**	**14**		

1.1. JOSEPH BONAPARTE GULF and NORTHERN TERRITORY (NT), AUSTRALIA

Yokoyama et al., 2000, 2001; DeDekker and Yokoyama, 2009; Nicholas et al., 2014; Ishiwa et al., 2015:

Material dated:
(1) mix of undifferentiated foraminifera and bivalve molluscs[1–3];
(2) species/genus specific [1,4,5] and;
(3) undifferentiated wood, plant matter[4,5], silty clay[1,2].
Unfortunately, for the dataset of [5], the $\delta^{13}C$ reported in was not measured offline and therefore cannot be used to infer the environment from which the sample comes (Yusuke Yokoyama, *pers. comm.*). The bulk sediment ^{14}C dates for the piston core LSDH-57 (Lab ID LJ-998 and LJ-999) were not calibrated in the original publications[1–3].

Elevation uncertainty: uncertainty not reported for water depth nor sampling uncertainty. Assigned a ± 1.5 measurement uncertainty (i.e., half the modern tidal amplitude as core recovered from a ship); Blacktip wellhead platform location measured tidal range of 5.8 m and neap tide is typically 2 to 3 m[182]. The present tidal range for the site is 3 m[1]. The sampling uncertainty is likely very small, allocated a sampling uncertainty of ± 0.01 m (cf.[183]).

Used all available data and the facies formation ranges quoted in original publications (see list below). Note, authors are confident about formation depth range for brackish facies but suggested range may be under-represented by current formation range for other facies (Yusuke Yokoyama, *pers. comm.*).
We use the following facies formation information in the database:

- Brackish, estuarine, intertidal: 0 ± 2 m (cf. [5])
- Marginal marine -4 +2/-4 m (cf. [184])
- Shallow marine -10 ± 5 m (cf. [185])
- Open marine -20 ± 5 m (cf. [185])

Jongsma, 1970 (Arafura Sea – off the Northern Territory, also in [186]):
Elevation: for samples from cores, assumed core was taken from a ship and that the elevation uncertainty is half the tidal range (tidal range quoted as in excess of 5 m[187]; for the submersible sample, we also assigned an elevation uncertainty of half the tidal range.

Facies formation: not reported for the samples from the cores (shell and wood material). For the beachrock sample (obtained by submersible), we use the generic beachrock relationship[188] (i.e., sample formed at MTL), therefore used the tidal range as the formation range, i.e., 0 ± 2.5 m.

1.2 NEW SOUTH WALES (NSW), AUSTRALIA

Gill, 1967 (various sites):
Elevations were converted to metres and we assume that "low water mark" is equivalent to LAT. The tidal range is 1.78 m (1.78 m = tidal range for Port Macquarie[189] (note, added 0.89 m to the elevation to give the elevation with respect to MSL).

Facies formation: no information on the modern formation depth range for the samples, therefore all limiting data. Age: assumed that the ages reported in the paper have not been $\delta^{13}C$ corrected but they have been background corrected. Applied appropriate $\delta^{13}C$ correction[190] prior to recalibration.

Switzer et al., 2010 (Batemans Bay, NSW):
Facies formation: Unit 1 (shelly sand) – "deposited within a few metres of contemporary sea level, suggesting that sea level at the time must have been at least 1 m higher". No further facies formation range given; used a range of 0 to + 2m referenced to MSL.
Unit 2 – unclear precise relationship to sea level at time of formation – may be a tsunami deposit.

Age: calibrated ages in paper reported in paper; obtained lab report (Adam Switzer, *pers comm.*) and dates reported as calibrated in the paper are actually the conventional radiocarbon dates. These have now been calibrated.

Ferland et al., 1995 (NSW coast):
Elevation uncertainty: assumed that the samples were obtained from a ship – allocated half the modern spring tidal range (± 0.55 m) as the measurement uncertainty (tidal range for Norah Head = 1.1m spring; 0.7 m neap[191]). No water depth given for the core but the core is plotted between the 120 and 140 m isobaths; have assumed an elevation of -130 ± 10 m.

Age: ages not calibrated in the paper; they have been corrected for isotopic fractionation (i.e. $\delta^{13}C$ corrected). No mention of background correction – assumed has been done. Authors also correct for the marine "reservoir effect for ocean surface waters adjacent to eastern Australia (Gillespie and Polach, 1979)"; the "apparent age" given by is 450 ± 35 years[175] which was added this back to the reported ages in the paper prior to recalibration.

Thom, unpublished; Shepard, 1970; Thom and Chappell, 1975 (NSW, Moruya):
Facies formation: no information available in the paper, therefore consider this limiting data. The "organic clay with shell" samples and "charcoal with estuarine shell" samples do not have a clear relationship to former sea levels and are considered unreliable sea level indicators. Ages cannot be recalibrated for these samples as uncertain what exactly was dated.

Age: these have not been $\delta^{13}C$ corrected; assumed they have been background corrected. Applied a correction of $\delta^{13}C = 0 \pm 2$ ‰ for marine carbonates using the spreadsheet for radiometric analyses[190] prior to recalibration.

1.3 SOUTH AUSTRALIA (SA), AUSTRALIA

Burne, 1982 (South Australia, Spencer Gulf):
Elevation given in relation of AHD and assumed this is equivalent to MSL. (Note, some samples have no reported elevation). Used the elevation of the base of the shell ridges (which are assumed to be a more reliable indicator of former sea levels[16]). Note, beach ridges may undergo modification (ridge crest elevation reduced by deflation, erosion by surface waters etc.; bases obscured by accretion of younger ridges, modified by flood waters etc., see discussion of [16]). In addition, the ages obtained from the shells sampled from the ridges date the death of the animal rather than the date of formation of the feature. Age of shells most likely reflects age of death rather than the age of the formation of the feature.

Belperio, 1979, 1993; Belperio et al., 1983, 1984, 1993, 2002; Short et al., 1986; Harvey et al., 1999 (various):
Used the data in the compilation of [15]
Facies formation depth: derived from local modern analogues[15].
- Ceduna facies information used for: Ceduna; Port Lincoln; Franklin Harbour
- Port Pirie facies information used for: Port Pirie
- Port Augusta facies information used for: Redcliff; Port Wakefield; Gulf St. Vincent
- Port Adelaide facies information used for: Port Gowler; Port Adelaide and Gillman

Age: are conventional radiocarbon dates (confirmed by author, Tony Belperio, *pers. comm*). Added 450 years to the marine samples and any seagrass sample with $\delta^{13}C > -11$ ‰ as the authors make a 450 ± 35 year correction for marine carbonates and organic remains (e.g., seagrass) with $\delta^{13}C < -11$ ‰. Calibrated the terrestrial samples using the Southern Hemisphere curve[192].

1.4 VICTORIA

Gill, 1967 Victoria (Melbourne):
Included all the tree samples; converted elevation to metres. Where reported referenced to LWM, assumed that LWM is equivalent to LAT and used the tidal range of 0.81 m for Willliamstown[193] and added half of this to the elevation to get elevation referenced to MSL.

Facies formation: no further information on the formation depth range for these samples therefore all the samples are limiting data. Samples are all tree stumps therefore sea level must be below the elevation of the sample at the time of growth.

Age: these have not been $\delta^{13}C$ corrected; assumed they have been background corrected. Applied a correction of $\delta^{13}C = 0 \pm 2$ ‰ for marine carbonates using spreadsheet for radiometric analyses[190] prior to recalibration.

Gill, 1968, 1971a,b,c; Gill and Hopley, 1972; Bowler et al 1966 (in [7]):
Facies formation: no information available in the paper, therefore this is considered limiting data. The tree samples give a maximum upper bound for sea level (i.e., sea level must have been below this point at the time of growth); for the shell data, sea level must have been above the elevation of the samples.

Age: ages have not been $\delta^{13}C$ corrected; assumed they have been background corrected. Applied a correction of $\delta^{13}C = -25 \pm 2$ ‰ for terrestrial organic material and $\delta^{13}C = 0 \pm 2$ ‰ for marine carbonates using[190] prior to recalibration. Note, we cannot correct the estuarine shell samples as there is the potential for mixing of fresh- and seawater.

1.5 TASMANIA

Gill, 1971b (in [7]):
Facies formation: no information available; sample is a tree stump therefore sea level must be below the elevation of the sample at the time of growth.

Age: ages have not been $\delta^{13}C$ corrected; assumed they have been background corrected. Applied a correction of $\delta^{13}C = -25 \pm 2$ ‰ for terrestrial organic material using[190] prior to recalibration.

1.6 QUEENSLAND (QLD), AUSTRALIA

Veeh and Veevers, 1970 (Great Barrier Reef, One Tree Island):
Elevation: not reported how elevation determined; assumed echo sounding from the ship and therefore used half the tidal range as the uncertainty. MTL = 1.7 m; MHW = 2.39 m and MLW is 0.69 m references to LAT at Heron Island[194]

No depth distribution given for the corals, inferred as shallow water (although the authors recognise that this species, *Galexea clavus*, has been found at -25 m in the Maldives and -75 m in Bikini Atoll and Jamaica (J.W. Wells, *pers. comm.* in[37]).

Ages: very little information in the original publication; assumed the radiocarbon dates have been background corrected but not $\delta^{13}C$ corrected. Applied a correction of $\delta^{13}C = 0 \pm 2$ ‰ using[190] prior to recalibration. U-series dates: insufficient information to recalculate the dates.

Chappell et al., 1983 (Great Barrier Reef):
Elevations were originally reported in relation to “low-tidal datum” (and we have assumed this is MLW), except for King Island, Flinders Island, Fantome Island and Great Palm Island where elevations are reported with respect to MLWS. Where the sample elevations are referenced to “low tide datum”, we allocate an additional uncertainty of 0.5 m (MSL-LAT) to account for the fact that we are uncertain exactly what tidal datum was used to reference the elevations of the samples originally.

For King Island, used the Grassy Bay, King Island tidal information[195] (note this location is not in [194]) to convert to elevations to relation with MSL (MSL is +0.9 m referenced to LAT; MLWS is +0.2 m referenced to LAT, therefore minus 0.7 m from the elevations to get elevation referenced to MSL).

For Fantome Island and Great Palm Island: used Lucinda (offshore) tidal information[194], MSL is at +1.89 m referenced to LAT; MLWS is +0.8 m referenced to LAT, and so minus 1 m from elevations to reference to MSL. “Low-tide datum”: assume this is MLW and added extra vertical uncertainty (i.e., half the difference between LAT and MSL) where MLW is assumed to be the mid-point between MLWN and MLWS.

Flinders Island tidal information[194]: MSL is at +1.52 m referenced to LAT; MLW (mid-point between MLWN and MLWS) is at +1 m referenced to LAT; therefore, to get elevation referenced to MSL minus 0.52 m. (uncert = ± 0.76)

For Yule Point: used the Port Douglas semidiurnal tidal information (2016)[194]; MSL is +1.60 m above LAT; MLW is +1.035 m above LAT; MLW (mid-point between MLWS and MLWN) is +1.035 m above LAT. MLW is therefore -0.565 m referenced to MSL, so to recalculate elevations referenced to MSL, subtract 0.565 m from the reported elevations (uncert = ± 0.8 m).

For Dunk Island: used the Dunk Island tidal information[194], MSL is at +1.79 m referenced to LAT; MLW (mid-point between MLWN and MLWS) is at +01.15 m referenced to LAT; therefore, to get elevation referenced to MSL minus 0.64 m. (uncert = ± 0.895)

For Goold Island: used the Goold Island tidal information[194], MSL is at +1.88 m referenced to LAT; MLW (mid-point between MLWN and MLWS) is at +1.2 m referenced to LAT; therefore, to get elevation referenced to MSL minus 0.68 m. (uncert = ± 0.94)

For Orpheus Island: used the Lucinda (offshore) tidal information[194], MSL is at +1.89 m referenced to LAT; MLW (mid-point between MLWN and MLWS) is at +1.16 m referenced to LAT; therefore, to get elevation referenced to MSL minus 0.69 m. (uncert = ± 0.945)

For Magnetic Island: used the Magnetic Island tidal information[194], MSL is at +1.91 m referenced to LAT; MLW (mid-point between MLWN and MLWS) is at +0.99 m referenced to LAT; therefore, to get elevation referenced to MSL minus 0.75 m. (uncert = ± 0.955)

For Camp Island and Stone Island: used the Bowen tidal information[194], MSL is at +1.76 m referenced to LAT; MLW (mid-point between MLWN and MLWS) is at +1.16 m referenced to LAT; therefore, to get elevation referenced to MSL minus 0.77 m. (uncert = ± 0.88)

Facies formation depth: used the formation depths given in the publication (range) i.e., the elevation at which modern microatolls at each reef form (note, the elevations are referenced to MSL using the same tidal parameters as converting the sample elevations for each site). Note, the moated microatolls are unreliable sea level indicators.

Age: assumed that the samples have been background corrected (authors state that they have been $\delta^{13}C$ corrected) and then recalibrated using the Marine13 calibration curve[196].

Kench et al., 2012 (Bewick Cay, northern Great Barrier Reef):
Only included the *in situ* fossil coral microatoll data, as well as the beachrock and mangrove peat samples. Other samples are not *in situ* or do not have an unambiguous relationship to sea level. No facies formation depth reported; used the highest living coral as the upper limit to growth (i.e., upper formation depth is -1.45 m referenced to MSL).

Ages are conventional radiocarbon dates which were recalibrated using the appropriate calibration curve and ΔR for marine samples (note, assumed the beachrock is marine).

Leonard et al., 2016. (Great Barrier Reef, Keppel Islands):
Elevations referenced (by the authors[31]) to MLWS (using tide gauge data from Rosslyn Bay). Using tidal planes from Great Keppel Island[194], MLWS is 0.76 m (as reported in [31]) and MSL is 2.43 m above LAT respectively. As such we convert the samples elevations so they are referenced to MSL (by subtracting 1.67 m from the elevation originally reported).

Ages have been recalculated. From email correspondence with the authors “We use a gravitationally made pure U metal standard to calibrate ^{234}U concentrations, and then the secular equilibrium HU-1 standard (the aliquot provided by Ken Ludwig) for spike ratio calibration” (N. Leonard, *pers. comm*. Calibrations by Jian-xin Zhao). Therefore, the activity ratios remain the same as those reported.

No % calcite reported; email correspondence with the authors “We did not conduct SEM (or EDS) analysis on the samples for this study. Selected samples were checked under SEM for a co-study (not yet published) and were found to be acceptable for dating. Our stringent cleaning and hand picking of aragonite chips allows for avoidance of sections of coral with calcite cements and detritus” (N. Leonard, *pers. comm*.). Reasonable to include this data in the analysis as the authors made stringent efforts to avoid calcite cements and detritus.

No modern depth distributions for the corals (i.e., the non-microatoll data); only the upper limit to growth given for the microatolls (i.e., MLWS). This limit (MLWS) is the generic limit for microatolls (cf.[197–199])

Yu and Zhao, 2010 (Great Barrier Reef, Magnetic Island):
Elevations reported in relation to the highest living coral (HLC) but there is no elevation reported for the HLC at each of the two sites. Included for reference.

Lewis et al., 2015 (Great Barrier Reef, Cleveland and Halifax Bays):
Elevations are referenced to LAT (using the Townsville tide gauge); MSL is +1.91 m[33] above LAT (figure 5[33]) but given as +1.94 m above LAT[194]. Converted the elevations so they are referenced to MSL using the information given in [33](i.e., subtract 1.91 m from the elevations).

Used the modern formation depths of the facies formation depth range (i.e., the observed modern elevation range of the oyster visors). For the barnacle data point, the modern ranges for the area is given as ~ 0.1 m below the modern oyster zone to ~ 0.4 m above the upper most oyster zone[33]. There is no explicit modern depth range given for the "oyster zone" but instead authors give a range from MHWN to MLWS[33]. As such, using the Townsville tide gauge data (as reported in [33] rather than [194]), the modern oyster zone is +0.2 to -1.3 m referenced to MSL. Therefore, the barnacle formation range would be +0.6 m to -1.4 m referenced to MSL.

Ages: assumed that these are conventional. ΔR: authors use 12 ± 7 years (marine carbonates central Great Barrier Reef[178]).

Lewis et al., 2012. (Nelly Bay, Magnetic Island):
No elevations reported for these samples; no elevations given for the coral bommie samples; no core top elevations given for the cores.
Ages: assumed radiocarbon ages are conventional. U-series – insufficient information to recalculate the age.

Lewis et al., 2008 (new data from Huntingfield Bay, compilation from E. Australia):
Elevations referenced to the elevation of the modern oyster bed. Recalculated the elevation referenced to MSL using the elevations given in figure 2 of the paper. No facies formation depth for the solitary oyster bed sample.
Ages assumed to be conventional radiocarbon dates. ΔR: authors use 12 ± 7 years (marine carbonates central Great Barrier Reef[178]).

Grindrod and Rhodes, 1984. (Missionary Bay, Queensland):
Elevation: cores taken from a barge; assigned half the reported tidal range as vertical elevation uncertainty (i.e., ± 2 m); Assumed the uncertainties associated with the coring method are similar to rotary and vibrocoring and allocated a ± 0.15 m uncertainty (cf. [200]). Elevation reported referenced to a tidal predication that is -1.55 m below the AHD. Converted all elevation so they relate to AHD (i.e., by subtracting 1.55 m from the reported elevation). Assumed that AHD is equivalent to MSL.

Used the facies formation depths given in the paper: note elevations recalculated referenced to AHD by subtracting 1.55 m. Assumed AHD is equivalent to MSL.

Age: Ages reported[27] have not been $\delta^{13}C$ corrected but have been background corrected. Shell samples have of 450 (± 35) years subtracted (cf.[175,201]), therefore, added 450 years to the reported radiocarbon ages of the shell samples. Applied a correction of $\delta^{13}C$ = -25 ± 2 ‰ for terrestrial organic samples and $\delta^{13}C$ = 0 ± 2 ‰ for marine carbonates (using[190]) prior to recalibration.

Thom, unpublished (in [7]) (Gold Coast):
Facies formation: no further information in the publication, therefore treat the data as limiting data (i.e., sea level below the elevation of the sample).
Age: radiocarbon dates were not $\delta^{13}C$ corrected in the original publication; we have assumed they have been background corrected. Applied a correction of $\delta^{13}C$ = -25 ± 2 ‰ for terrestrial organic samples (using [190]) prior to recalibration.

Woodroffe, 2009 (Cleveland Bay):
New data and compilation of other data for the area ([24] reassessed the relationship to sea level for older publications – see below for details)

For the new data from Cocoa and Alligator Creek[24]; no details on how sea level quoted in the paper was calculated i.e., there is insufficient information to recalculate the indicative range and reference water level that would allow for the recalculation of RSL. Also, the elevation uncertainties are not detailed and so cannot back calculate the indicative range.

Woodroffe, 2009; Carter et al, 1993; Larcombe et al., 1995; Larcombe and Carter, 1998; Ohlenbusch, 1991, Harvey et al., 2001; Horton et al., 2007; Spenceley, 1980; Beaman et al., 1994; Tye, 1992; Belperio, 1979 (Cleveland Bay, Townsville, Halifax Bay):

Beaman et al., 1994: in the database we used the elevations in [24]. Note, the elevations in [20] are referenced to AHD (which is assumed to be equivalent to MSL in the Townsville area[20]) but there is a 0.1 m difference between AHD and MTL for the area based in the difference between the two quoted elevations for the same sample.

Elevations are reported referenced to MTL by [24]; using the Townsville tidal plane data[194], MSL = 0.084 m above AHD; MTL (i.e., the arithmetic mean of MLW and MHW) is 0.0025 m above MSL, therefore for Townsville, we have assumed that MTL is equivalent to MSL (Note, there is a slight discrepancy in the elevation of MSL above LAT at Townsville between Permanent Service for Mean Sea Level (PSMSL)[202] and the Queensland Government[194] information; we have used the latter as this also lists the tidal planes needed to work out the RWL and IR for the samples in Table 1(but the elevation of AHD referenced to LAT is the same for both sources). Townsville tidal planes referenced to MSL[194] and assuming MTL~MSL: HAT = +2.17 m; MHWN = +0.32 m; MTL = 0; MLWN = -0.31 m; MLWS = -1.17 m.

Also, a greater facies formation depth is given for the species of oyster[24] due to the confined location of the samples i.e., within a cave) where they would experience wave splash. Have used this wider formation depth range[24].

1.7 TORRES STRAIT, AUSTRALIA

Woodroffe et al., 2000:
Samples are corals and microatolls. The elevations of both the corals and the microatolls are reported referenced to the highest living coral (HLC). Unable to convert these to a tidal datum. Included for reference.
Age: conventional ^{14}C dates. Calibrated using the appropriate calibration dataset and ΔR.

2. NEW ZEALAND

Used the uplift rates given in [203]but note, the rates of subsidence have not been constrained in [203].

ΔR: used different regional ΔR determinations as outlined below.

East coast of the South Island: bathed in waters of the Southland Current[204]; we use a weighted mean of the two samples in online database[173,205,206]. ΔR = -5 ± 47 (n=2). Note for samples near Christchurch, have used the Kairaki data point[205] (ΔR = 25 ± 35).

Location	ΔR (years)	± 1σ	source
Kairaki	25	35	[205]
Pounawea	-42	39	[206]
**Weighted mean**	**-5**		
**Standard deviation**	**47**		
**n**	**2**		

Northern coastline of the North Island: two sites in the online database[173] (East Coast[205] and Awani Bay, East Cape[207], both of which are influenced by the same surface water masses (East Cape Current, e.g., [204]). Used a weighted mean from the samples at these two sites; ΔR = 12 ± 56 (n=7)

Location	ΔR (years)	± 1σ	source
East coast	-20	57	[205]
East coast	-108	61	[205]
East coast	46	58	[205]
East coast	-35	59	[205]
East coast	-1	65	[205]
East coast	77	57	[205]
Awani Bay, East Cape	39	31	[207]
**Weighted mean**	**12**		
**Standard deviation**	**56**		
**n**	**7**		

North Island, east coast: no determinations from the east coast of the North Island in online database[173] (i.e., poorly constrained for this region). Shelf current flows from the south, whilst the East Cape Current flows in the opposite direction further offshore[204]. Used samples from Turakirae Head (southern tip of the North Island[208]) and the single determinations from Awani Bay, East Cape on the NE coast of the North Island[207]: ΔR = 10 ± 25 (n=11).

Location	ΔR (years)	± 1σ	source
Turakirae Head	-10	47	[208]
Turakirae Head	-17	41	[208]
Turakirae Head	35	43	[208]
Turakirae Head	6	35	[208]
Turakirae Head	7	36	[208]
Turakirae Head	-9	48	[208]
Turakirae Head	36	44	[208]
Turakirae Head	26	29	[208]
Turakirae Head	-31	45	[208]
Turakirae Head	-29	50	[208]
Awani Bay, East Cape	39	31	[207]
**Weighted mean**	**10**		
**Standard deviation**	**25**		
**n**	**11**		

Southern tip of the North Island: four sites with determinations in the online database[173,205,208]; used a weighted mean of these; ΔR = -7 ±31 years (n=15).

Location	ΔR (years)	± 1σ	source
Paekakariki	-40	46	205
Makara Beach	-47	62	205
Makara Beach	-5	62	205
Makara Beach	-79	44	205
Pauatahanui Inlet	-18	32	205
Turakirae Head	-10	47	208
Turakirae Head	-17	41	208
Turakirae Head	35	43	208
Turakirae Head	6	35	208
Turakirae Head	7	36	208
Turakirae Head	-9	48	208
Turakirae Head	36	44	208
Turakirae Head	26	29	208
Turakirae Head	-31	45	208
Turakirae Head	-29	50	208
**Weighted mean**	**-7**		
**Standard deviation**	**31**		
**n**	**15**		

Gibb, 1986 (and references therein[42–47,49,51,52]) (North Island, various sites):
Elevations are reported referenced to their modern analogues, some of which occur at MSL. The remainder (i.e., those referenced MHWS, HAT etc.) were corrected to a MSL datum using tidal parameters from the most proximal tide gauge stations[209]: Blueskin Bay (used Port Chalmers); Weiti River, Kaiaua, Kellys Beach and Miranda (used Auckland); Christchurch (used Lyttleton); Pauatahanui and Kumenga (used Wellington).

Authors state that sampling uncertainty ranges from ± 0.03 m for Weiti River to ± 2.15 m for the Christchurch samples. This upper value seems very high and rather than allocate ± 2.15 m for the sampling error for the other sites, have used ± 0.25 m (rather than the usual ± 0.01 m when no sampling uncertainty is given in the original papers).

Formation depth range: the authors give the modern reference water level for the indicator (i.e., the elevation referenced to some tidal water level at which the indicator is currently forming e.g., MSL, MHWS). The authors then use the spring tidal range as the uncertainty (i.e. reference water level (RW) ± half the reported spring tidal range) with an additional ± 0.5 m (except for those from the upper tidal flats where the additional uncertainty is ± 0.25 m). The uncertainty is therefore the same for all the different indicators from the same site (rather than being specific to the indicator type). Followed this methodology but ensured that the RW was referenced to MSL (using the tide gauge information listed above).

Uplift/subsidence rate: Authors calculate the uplift rates by assuming that both the Blueskin Bay and the Weiti River sites are tectonically stable. For Blueskin Bay the presence of inferred Last Interglacial age terraces at ~ 6 to 8 m above present MSL along the Otago Peninsula (Benson, 1968) and the sheltered nature of the site lead Gibb (1988) to assume this site was stable for the last 125 ka. For the Weiti River estuary site the presence of raised shoreline (chenier) at ~ 4.6 m (of assumed Last Interglacial age; now obliterated by development) (Turner and Bartrum, 1929; Ferrar, 1934) and an equivalent Holocene analogue at 0.5 m is thought to indicate stability at the site since the last interglacial. The uplift/subsidence rates for the other sites used were derived by fitting the data to the curve created by the Blueskin Bay and Weiti River data (i.e., no independent estimate of the rates of uplift and no uplift uncertainty).

Radiocarbon ages: all reservoir corrected but to a value of -41 %; “All ages are “reservoir corrected” in terms of $\Delta^{14}C$ with respect to the New Zealand shell standard of -41 % (Jansen, 1984)”. Note, -41 % $\Delta^{14}C$ is equivalent to ~336 years. After cross checking the WK samples listed in Table 1 of Gibb 1986 against the original Woodroffe et al., 1983 paper, the marine ages reported in Gibb 1986 have been reservoir corrected using and age of 330 years. We have added this back to the WK samples ages reported in Gibb 1986. We assume that the samples have been both background and $\delta^{13}C$ corrected.

Ota et al., 1983, 1988; **Singh, 1971; Yoshikawa et al., 1980; Berryman, Boag, Brown, Ghani, Gibb, Landias, all unpublished** (in [41]) (North Island, various sites):
Assumed that all samples were taken from cores obtained by hand auger except for the *in situ* tree samples. In the latter case, assigned a ± 0.5 m uncertainty to account for errors in excavating/extraction of the sample (note, this is in addition to the ± 0.3 m levelling uncertainty). Where the elevation is labelled as c. X m, have added an additional ± 0.5 m vertical uncertainty. For sample NZ-1149, the elevation is given as HWM (high water mark) but

there is no tidal information for this site (Kaiwhata River). Used the information for Castlepoint (to the north of the site but closest in the New Zealand Nautical Almanac 2017-2018, secondary ports[210]). Assumed the HWM is equivalent to MHWS. At Castlepoint, MHWS is at +1.7 m referenced to datum; MSL is +0.8 m and so referenced to MSL, MHWS is at +0.9 m. Used +0.9 m as the elevation for this sample.

Facies formation depth: only generic depth range given for all wood and shell samples (i.e., that they represent MSL ± 2 m at the time of formation). Not clear how this was determined; assemblage only of the fossil shells and no mention of any modern analogue work (assumed none). Used MSL ± 2 m as the formation depth range.

Similarly, the *in situ* tree samples are thought to represent the maximum of sea level at the time of tree growth (i.e., that sea level must have been below the elevation of the trees (not clear by how much). Therefore, assigned an upper limit of formation to 0 m (referenced to MSL).

Uplift/subsidence rate: The authors derived uplift and subsidence rates by fitting the data to the sea level curve of Gibb (1986) i.e., the uplift/subsidence rates quoted are calculated from the elevations of the samples themselves. Authors state “considerable differential tectonism in the coastal area”; subsidence of coastal plains inferred. Potentially complex tectonic activity and coseismic uplift; used the uplift rates in [203].

Ages: discussion in [48]suggests that only the NZ- samples have been $\delta^{13}C$ corrected. GAK- samples have 600 years subtracted to account for the $\delta^{13}C$ correction to 25 ‰ and “Δ14C to -41 ‰ (Jansen, 1984)”. Note, the ages were not calibrated in the original publications.

PACIFIC OCEAN

1. SOCIETY, TUAMOTU, GAMBIER, and AUSTRAL Islands.

ΔR: one value in the online database[173] from the Austral Islands (ΔR = -3 ± 17 years[211])

Location	ΔR (years)	± 1σ	source
Austral Islands	-3	17	211
**Weighted mean**	**n/a**		
**Standard deviation**	**n/a**		

ΔR: three values from the Gambier Islands (ΔR = -3 ± 17 years[211]) which when recalculated using the online database[173] give a ΔR = -2 ± 23 (n=3).

Location	ΔR (years)	± 1σ	source
Mangareva, Vaiatekeue Island	22	19	211
Tearie Bank (18m)	-24	19	211
Mangareva Atoll	-3	20	211
**Weighted mean**	**-2**		
**Standard deviation**	**23**		
**n**	**3**		

ΔR: six samples from the Society Islands[211,212] in the online database[173]; we used a weighted mean of these values. ΔR = 17 ± 21 years (n=6).

Location	ΔR (years)	± 1σ	source
Outumaoro, Tahiti	23	19	211
Papeete, Tahiti	4	19	211
Taravao (under stones), Tahiti	-3	20	211
Taravao, Tahiti	22	17	211
Tahiti	46	42	212
Moorea	82	42	212
**Weighted mean**	**17**		
**Standard deviation**	**21**		
**n**	**6**		

ΔR: one value from the Tuamotu Archipelago (ΔR = 6 ± 17 years[211]) in the online database[173]; note the authors exclude the other Tuamotu sample (Marutea Sud Atoll) as thought to not be in equilibrium with the open ocean (i.e., in a lagoonal setting and the potential for shellfish to incorporate both fresh and sea water)

Location	ΔR (years)	± 1σ	source
Austral Islands	6	17	211
**Weighted mean**	**n/a**		
**Standard deviation**	**n/a**		

Chevalier and Salvat, 1975; Delibrias et al., 1974; Montaggioni, 1985; Pirazzoli, 1985, 1987; Pirazzoli et al., 1985a, b, 1987a, b, 1988a, b; Pirazzoli and Montaggioni, 1984, 1987, 1988 (various):
(Most from a compilation[213] of data from the Society, Tuamotu, Gambier and Austral Islands, double checked in the original publications[54–56,58,60–65,67,214,215])

Most samples are microatolls (included for reference); unable to calculate facies formation depth range for algal ridges or bivalve data. No species given for the coral framework samples. These can be used as limiting data.

Hv- and P- samples have been $\delta^{13}C$ corrected[213]. Authors subtract 400 years from the marine samples to account for the marine reservoir effect, therefore have added 400 years on to the ages quoted in the table. Assumed that for the other samples, these have not been $\delta^{13}C$ corrected, nor has there been a 400-year subtraction made to

marine samples. Assumed all samples have been background corrected. Where appropriate, we apply a correction of $\delta^{13}C = 0 \pm 2$ ‰ for marine carbonates (using the appropriate correction spreadsheet[190]) prior to recalibration.

2. SOUTHERN COOK ISLANDS

Goodwin and Harvey, 2008 (Aitutaki, Southern Cook Islands):
Elevations of the fossil corals were surveyed referenced to height of living corals (HLC); authors surveyed elevations of many living corals (Rarotonga n=400 observations) to give a mean HLC of -0.36 ± 0.008 m referenced to MSL (authors give the LAT, MLWN and MLWS in text referenced to MSL)[69]. To convert the elevations referenced to HLC to MSL add -0.36 m to the elevations of the fossil corals.

Uplift/subsidence rate: authors -0.01 m/ka stating no coseismic uplift for either island[69]. Authors resurvey the elevation of the limestone reef at Ngatangaia (pre-Holocene and presumed Last Interglacial age[216–218]) as 2.5 ± 1 m (elevation given as 3.5 m in [218]). Recalculated the subsidence rate using the 2.5 ± 1 m elevation of the Last Interglacial terrace; assumed Last Interglacial sea level (6.6 ± 2 m[219,220]) and Last Interglacial age (125 ± 5 ka) to give a rate of +-0.03 ± 0.02 m/ka (cf. [221]).

Age: assumed ages are conventional radiocarbon dates. For ΔR, the authors use ΔR = 57 ± 23 years. The online database[173] contains three values from Rarotonga and Mangaian Island in the Southern Cook Islands[211,222]. We exclude the Mangaia Island reef data point due to regional hardwater effect[211]. We combine a previous ΔR regional average for the Southern Cook Islands (ΔR = 15 ± 31 years[211]), with the Rarotonga data[222], recalculated in the online database[173] as a weighted mean of ΔR = -15 ± 38 (n=3). Note, we use this recalculated value of ΔR for all of the Southern Cook Islands.

**Location**	**ΔR (years)**	**± 1σ**	**source**
Rarotonga	11	17	211
Rarotonga	-52	27	222
Mangaia	-51	30	211
**Weighted mean**	**-15**		
**Standard deviation**	**38**		
**n**	**3**		

Moriwaki et al., 2006 (Rarotonga, Southern Cook Islands):
Elevations are reported referenced to MSL (see text rather than the table in [70] for details). Only included the coral microatolls (we have assumed that they are microatolls as the authors use the tidal range as the formation depth range). Rejected all other data as: no elevations reported or the material dated is not *in situ* or there is no unambiguous relationship to sea level.

Used the same subsidence rate as the other Southern Cook Island data[69] using the elevation of the assumed Last Interglacial terrace (see discussion in [69] and section above).

Facies formation: not reported by the authors (they use half the modern tidal range). As we have assumed these are microatolls, we use the facies formation information of [69].

Age: used the regional ΔR = -15 ± 38 years[173,211,222] (see section above for further details).

Uplift rate: recalculated using the maximum elevation +3.5 m[218,223,224] of presumed Last Interglacial age corals and an allocated uncertainty of ± 1m, an assumed Last Interglacial age (125 ± 5 ka) and sea level (6.6 ± 2 m[219,220]) to give a rate of -0.02 ± 0.01 m (cf. [221]).

Allen et al., 2016 (Rarotonga, Southern Cook Islands):
Elevation reported referenced to HLC. Recalculated elevations so they are referenced to MSL using the mean elevation of living corals from [69] (note, [69] use the HLC surveyed in Rarotonga to correct their Aitutaki samples and we follow this methodology here); we use the authors quoted elevation uncertainty[68].

Facies formation depth: not reported in the paper; used the facies formation information in [69].

Age: assumed that the ratios quoted are the activity ratios. Recalculated ages assuming that no conversion of the ratios is required (i.e. as authors used both gravimetric and SE standards to calibrate their). Recalculated the ages using the decay constants of [225] assuming a closed system (using Isoplot[226]).

Uplift rate: recalculated using the maximum elevation +3.5 m[218,223,224] of presumed Last Interglacial age corals and an allocated uncertainty of ± 1m, an assumed Last Interglacial age (125 ± 5 ka) and sea level (6.6 ± 2 m[219,220]) to give a rate of -0.02 ± 0.01 m (cf. [221]).

Yonekura et al., 1988 (Mangaia Island, Southern Cook Islands):
Facies formation depth for the modern microatolls given as mean low water, which the authors state of equivalent to -0.2 m (referenced to MSL)[71]. Used this as the upper limiting formation depth for the fossil microatoll samples (authors don't use any formation depth or depth habitat information, latter not considered in this study, i.e., no modern analogue). Rejected unidentified corals. Spring tidal ranges estimated as 0.8 m[71].

Radiocarbon determinations carried out by the Geological Survey of Japan (but can't find this lab in the Radiocarbon list). Measurements by benzene liquid scintillation. Assumed there has been no $\delta^{13}C$ correction applied; assumed background corrected. Applied a correction of $\delta^{13}C$ = 0 ± 2 ‰ (using the correction spreadsheet[190]) prior to recalibration. Ages not calibrated in the original publication.

Uplift rate: two major raised shorelines at +26 to 27.5 m and +18 to 20 m cut into elevated limestone terrace (makatea, itself thought to be mid Tertiary in age[223]). U-series ages for fossil corals at + 2m dated at 90 to 100 ka[227]; unpublished U-series ages of 101 to 135 ka from reef deposit +20 m above present sea level[224]. A maximum elevation of +15 m[218](table 1[218], sample age 118 ± 2 ka) is given for samples dated in [224] but [218]use an elevation of +14.5 m (their table 2) as the max. elevation of the MIS 5e feature. Derivation of the elevations given in [71] uncertain (i.e., the shorelines at +18 to +20 m of presumed Last Interglacial age). Note, [217] uses a mean of +12.4m (referenced to low tide level = +11.6 m when referenced to MSL). For Mangaia island, we have used the maximum elevation (+15 m) of the samples dated by [224]and reported in the table 1 of [218]to recalculate the uplift rate using the max elevation of the Last Interglacial terrace, an assumed Last Interglacial age (125 ± 5 ka) and sea level (6.6 ± 2 m[219,220]) (cf. [221]).

SOUTH AMERICA

1. ARGENTINE SHELF

Guilderson et al., 2000:
Used only the "clean" data as determined by the authors (criteria… "remove the clearly reworked samples". In essence we have kept the "youngest" and stratigraphically highest samples unless the results are indistinguishable given realistic errors"[72]).

Elevation uncertainty**:** Broadness of the shelf produces some of the largest tidal ranges in the world; for elevation uncertainty used half the tidal range and allocated ± 0.01 m for sampling uncertainty.

Age: used only the "clean" data. Material dated, either shell hash OR an individual shell. Note, no details on the species dated is given in the text, and no further information is available as to the species/genus dated (T. Guilderson, *pers. comm*.). Recalibrated the dates using appropriate calibration curve[196] and ΔR (see discussion below).

ΔR: Note the data of [228] is compromised by the possibility of mislabelling (and incorrect collection date); the effect of incorporated dissolved carbonates (even for the samples from the south-eastern coast of Argentina) leading to "serious and even unbridgeable limitation"[228] for radiocarbon chronologies for the region. These authors[228] recommend using ΔR = 0, and so we use this value to recalibrate the Argentine Shelf data. Note, [72]apply a ΔR = 600 years[212], but this derives from a sample in the Falkland current (i.e., a different water mass to locations of the samples in [72]). Note, that upwelling of old waters on to the continental shelf is thought to be minimal due to the dominant westerly winds[228].

No facies formation depth given for the samples dated. Generic statement that the deposits are "littoral to shallow neritic". The species composition of many of the cores dated[72] was investigated[229] , some of which we can assign modern depth distributions[230–232], however we cannot ascertain the exact species (or genus) dated and so we are unable to assign a depth habitat range (or hence calculate P_{RSL}). Based on examples from New Zealand[233], fossil shell beds are thought to form from high tide to depths of around 10 m. Unclear whether the shell hash dated is formed as a result of sorting and if so, this may mean that the dated material may not be contemporaneous with the depth interval (i.e., a time-averaged sample). Alternatively, the shell fragmentation could be a result of wave action (i.e. very shallow water/beach environments).

As such, we have not attempted to assign a formation depth range for these samples. Instead, we treat these as limiting sea level indicators, with sea-level likely above the elevation of the dated shells/shell hash.

INDIAN OCEAN

1. MAYOTTE

ΔR: used value for Mayotte ΔR = 119 ± 57 years, as the other value in the online database[173] was thought to be an outlier by the original authors[180].

Location	ΔR (years)	± 1σ	source
Mayotte, Comoros	119	57	180
**Weighted mean**	**n/a**		
**Standard deviation**	**n/a**		
**n**	**1**		

Zinke et al., 2003:
Limited information on what was dated and the formation depth range for each facies[75]. Note, currently unable assign depth distribution to any of the coral samples as no species given. Similarly, for the facies formation depth, only mangroves have a stated formation depth range (although somewhat unclear "mangroves are excellent sea-level indicators, since they mark the uppermost level of the intertidal flat at about ±0.5 m"[75]). Used a generic relationship for the formation depth (cf. [234–236]), with the facies forming between mean sea level and mean high water datum, i.e., between 0 to +2 m referenced to MSL (i.e., RWL of 1 ± 1 m). Tidal range: Mayotte macro-tidal (spring tidal range > 3m[237]; with the maximum tidal range known at Dzaoudzi, Ile de Mayotte, of 4.05 m[238]).

Subsidence rate: rate given in [75] is based on the max elevation of a coral "reef top" at a modern depth of -20 below present sea level and an assumed MIS 5a age[73] to give a subsidence rate of -0.2 to -0.25 m/ka. We have used a subsidence rate of -0.21±0.02 m/ka [221].

Unsure what exactly has been dated – e.g., "mangrove mud" - the analytical uncertainty may not encompass the entirety of the age uncertainty, as we cannot establish exactly what has been dated. Assumed that the dates have been background and $\delta^{13}C$ corrected (not explicitly stated but we have assumed that the "14C-age" is the conventional radiocarbon date).

Colonna et al., 1996:
Coral U/Th but these are α analyses. Very high [^{232}Th]. Where no ratio uncertainty is given, we have used 1×10^{-10} to recalculate the age.

Camoin et al., 1997:
Coral U/Th but these are α analyses. Very high [^{232}Th].

2. MAURITIUS*:*

ΔR: three values from samples from Mauritius[180], one of which they designate as an outlier; [180] acknowledge the lack of coherence between their dataset and that of [239] (Maldives ΔR = -40 ± 35). Used a weighted average of the two Maldives samples[180] (i.e., excluded the outlier identified by these authors); ΔR = 122 ± 5 years (n=2).

Location	ΔR (years)	± 1σ	source
Ile de France, Mauritius	125	40	180
Mauritius	118	42	180
**Weighted mean**	**122**		
**Standard deviation**	**5**		
**n**	**2**		

Camoin et al., 1997:
Coral U/Th but these are α analyses. Very high [^{232}Th].

Montaggioni and Faure, 1997:
Elevations are referenced to "average low tide level" but unclear whether the authors mean MLW, MLWS, MLWN, LAT, (Admiralty) Chart Datum etc. As such, we have estimated the elevation of the core top depth by estimating the max and min based on the datum being MLWS and MLWN respectively to give core top elevation and uncertainty. This uncertainty is then incorporated into the final elevation and uncertainty (reassuringly, this overlaps with the originally reported depth).
Ages are conventional radiocarbon dates (i.e., background and $\delta^{13}C$ corrected).

3. MALDIVES

Maldives ΔR: no determinations in the online database[173]; potentially complex modern oceanography for the region with a reversal in currents during the monsoon. Previous estimates of ΔR: [78,79] use ΔR = 132 ± 25, quoting[180] but no such value in this paper, instead [180] use an average value for the western Indian Ocean of ΔR = 158 ± 68 years[180] (which becomes 167 ± 99 years using the calculation and values in the online database[173], using the same values[180]). Paula Reimer (*pers. comm.*) suggests using values for the eastern Arabian Sea and the tropical southwestern Indian Ocean (Seychelles etc.) as this mostly encloses the surface waters surrounding the Maldives. Used ΔR = 135 ± 76 years for all locations.

Location	ΔR (years)	± 1σ	source
Sri Lanka (601)	101	47	180
Sri Lanka (602)	187	53	180
Sri Lanka (603)	198	53	180
Sri Lanka (604)	63	50	180
Malabar (600)	138	64	180
Goa (599)	252	51	180
Bombay (597)	165	57	180
Bombay (598)	113	50	180
Diego-Suarez (Madagascar) (481)	177	60	180
Seychelles (482)	310	59	180
Seychelles (483)	147	57	180
Seychelles (484)	87	57	180
Seychelles (485)	50	57	180
Mahe (Seychelles) (486)	150	62	180
Ile de France (Mauritius) (478)	125	40	180
Mauritius (479)	118	42	180
Mauritius (480)	-50	57	180
**Weighted mean**	**135**		
**Standard deviation**	**76**		
**n**	**17**		

Kench et al., 2005:
Elevation referenced to MSL but no uncertainties given; allocated a ± 0.5 m elevation uncertainty (no method reported); a ± 0.15 m coring uncertainty (method not specified); and a ± 0.01 sampling uncertainty. Material dated: mostly skeletal carbonates. The authors suggest that these are *in situ* deposited in a lagoon or backreef setting (velu and finolhu facies; dominated by un-fragmented *Halimeda* grains)[78]. No facies formation given: velu and finolhu facies could be limiting (sea level above) data given their deposition in lagoon or backreef setting. ΔR: [78] use 132 ± 25 (quoting [180] but we have not found this value in the paper) – see discussion above for ΔR used.

Kench et al., 2009:
Cored corals. Elevation referenced to MSL but no uncertainties given[79]; allocated a ± 0.03 m uncertainty associated with auto-level determination of elevation; ± 0.15 m uncertainty associated with rotary drilling and; ± 0.01 m associated with sampling uncertainty.
Ages are conventional radiocarbon dates; see discussion above for ΔR used.

Gischler et al., 2008:
Corals and one microbiolite sample. Elevation referenced to MSL but no uncertainties given; allocated a ± 0.5 m elevation uncertainty (no method reported); a ± 0.15 m coring uncertainty (method not specified); and a ± 0.01 sampling uncertainty. Assumed the “measured age” is a conventional radiocarbon date. ΔR: authors[77] use 25 ± 50 years (no reference given) – see discussion above for ΔR used.

Woodroffe, 1993:
Mainly corals with two coral sand samples. Assumed the elevation uncertainty relates only to how the elevation was derived (i.e., not the method of coring nor the sample uncertainty, allocated these where appropriate). No modern survey of coral assemblage undertaken and no palaeo-water depth assignment in the original publication[80]. We exclude the coral sand samples (unknown provenance and formation). The samples were not calibrated in the original publication. No ΔR quoted by the authors (not calibrated) – see discussion above for the value of ΔR used in the recalibration.

4. ZANZIBAR

Woodroffe et al., 2015:
Ages: Mangroves potentially problematic for dating due to the penetration of roots and the incorporation of anomalously young carbon into the system (see comparison and discussion of the bulk sediment radiocarbon and concentrated organic samples[81]). In our database, we include only the mangrove organic concentrate (10 to 63 µm) size fraction radiocarbon dates. The authors[81] use this small fraction for dating to avoid large organic fragment (potential source of young carbon) and this is the fraction they used for pollen analysis. The mangrove concentration provided ^{14}C dates that were in stratigraphic order (unlike the previous bulk sediment samples).

INDIAN SUBCOTINENT

1. BAY OF BENGAL

Wiedicke et al., 1999 (outer Bengal Shelf):
Difficulty in demonstrating the relationship between the oolitic beach barriers to past sea levels[240]. In addition, there is the potential for transport of material e.g., "…has to keep in mind that our cores sample allochthonous material…and that these samples cannot be linked beyond doubt to a particular ridge"[82] (i.e., the material originated at some distance from their present position). No facies formation depth given for any of the samples (molluscs and ooids). Authors[82] assume that the ooids formed in very shallow, high-energy nearshore environment[241,242]. However, the assumption of ooid formation in very shallow water (i.e. < 2 m water depth) has been questioned[240] (and we suspect compilation of [243] may have used this (i.e., 2 m) as the vertical uncertainty). No species given for the molluscs dated (and therefore we cannot assign a depth habitat range).

Elevation: Bay of Bengal tidal range: Tide gauges M2 tidal constituent: 92, 90, 73 cm at Short Island (20° 47'N; 87° 04' E), India, Dhamra, India (20° 48'N; 86° 54' E) Chandbali, India (20° 47'N; 86° 44'E) respectively; "maximum tidal range is only about 2 m"[244]. Bangladesh tide gauges 72 to 136 cm M2 tidal range[244]. M2 semidiurnal highest amplitude of 1.8 m in the Gulf of Martaban[245]. We have assumed that the tidal range is ~ 1 m in the region (given the various M2 tidal constituents[244]) and as such, we have assigned a ± 0.5 m elevation uncertainty (none reported in the paper, water depths obtained by echo sounding[82]).

Subsidence rate: Authors suggest little significant subsidence of the outer Bay of Bengal; subsidence south of Calcutta 0.6 m/ka since the Miocene; 0.2 m/ka since the Pliocene[246]. The average Bangladesh subsidence rates "might not apply far out on to the shelf" given that the edge of the continent shelf "lies at about 150 m, not much lower than the world-wide average of about 120-130 m"[247]. The preservation of the ridges is thought to be due to sediment bypassing[82,248]. As such we have assumed that the site is stable (as per the original publication[82])

Age: dates are conventional ^{14}C dates but the they had 400-years subtracted (for the global reservoir) - statement under table 1 that a "reservoir correction of 400 yrs was applied to all data"[82]; no mention of calibration in the original publication.

ΔR: no determinations for the northern Bay of Bengal in the online database[173]. Indian Ocean average = 158 ± 68 years[180] (n=31). An average of three locations in the northern Indian Ocean (included in [180]) suggests average of ΔR = 11 ± 35 for the eastern Bay of Bengal (Andaman Sea) and 32 ± 20 years for the southern Bay of Bengal[249]. The Bay of Bengal receives a large volume of freshwater from the north which reduces vertical mixing rate preventing advection of deeper ^{14}C depleted water resulting in younger reservoir ages[249]. However, riverine dissolved inorganic carbon (DIC) depleted in ^{14}C will tend to counteract this effect[250]. We have used the determinations fringing the Bay of Bengal in the online database[173] (which are likely to encompass all of the variability expected in the basin) to give a weighted mean of ΔR = 55 ± 139. We have excluded the Pondicherry sample[180] as it is thought to be anomalous and may be a result of freshwater influence (input of geologically, radiocarbon "dead" carbonate material)[180].

Location	ΔR (years)	± 1σ	source
Chilika Lake, Orissa (lagoon)	-60	51	[249]
Stewart Island, N. Andaman	12	34	[249]
Nicobar Islands	32	70	[180]
Rameswaram, Tamilnadu	29	34	[249]
Rameswaram, Tamilnadu	30	34	[249]
Mandapam, Tamilnadu	42	34	[249]
**Weighted mean**	**20**		
**Standard deviation**	**30**		

2. SOUTHERN INDIA AND SRI LANKA

Banerjee, 2000 (India):
Excluded:
- any data that is older the deglacial period i.e., [251] data in Table 1 and all data in Table 2[83]
- all data in Table 4 – the ages determined for these samples are thought anomalous (i.e., rejected by the authors[83])
- all data in Table 5[83] as there are no elevations listed for the samples.

Elevation: Measured referenced to "low tide level (LTL)" but this is not defined[83]. Assumed this LTL equates to chart datum and used the tide information from Permanent Service for Mean Sea Level for the closest tide gauge station (see below) (i.e., subtracted the difference of the chart datum and MSL from the elevation given in Banerjee, 2000).

- Rameswaram Island sites – used the Tangachchimadam tide gauge[252] (PSMSL station ID 1258; 9.28 N; 79.25 E) (subtracted 0.412 m from the elevations):
- Cap Corin and Gulf of Mannar – used the Tuticorin tide gauge[253] (PSMSL station ID 1072; 8.75 N; 78.2 E) (subtracted 0.633 m from the elevations):
- Godabari inter-deltaic sites – used the Vishakhapatnam tide gauge[254] (PSMSL station ID 414; 17.68 N; 83.28 E) (subtracted 0.752 m from the elevations)

Authors assume that the region is tectonically stable (southern Indian, Precambrian shield)[83].

Facies formation: no detailed information given in the publication; bivalve and gastropods assumed to be upper intertidal (and within a microtidal range, "the biota since this tectonically stable coast of microtidal and the uncertainty on the estimated sea level elevation is at most ± 0.9 m"[83]).

- Intertidal: used 0 ± 0.9 m as the facies formation range.
- Intertidal to supratidal: used -0.9 to 1.4 (i.e., added and additional 0.5 m to the tidal range)
- Upper intertidal: used 0 to +1.9 m

Age: assumed the radiocarbon dates are conventional. ΔR: seasonally reversing monsoon winds and surface circulation in southern Indian and Sri Lanka with the Northeast and the Southwest monsoon (e.g., [255]); coastal upwelling in the waters surrounding Sri Lanka, major region during both monsoon periods is along the southern coast. The values for Sri Lanka (in the online database[173] derived from [180]) are very different from the southern Indian values[249]. Used a weighted average of the Sri Lankan[180] and southern Indian data (Tamilnadu only[249]); ΔR = 71 ± 64 (n=7)

**Location**	**ΔR (years)**	**± 1σ**	**source**
Rameswaram, Tamilnadu	29	34	[249]
Rameswaram, Tamilnadu	30	34	[249]
Mandapam, Tamilnadu	42	34	[249]
Sri Lanka	101	47	[180]
Sri Lanka	187	53	[180]
Sri Lanka	198	53	[180]
Sri Lanka	63	50	[180]
**Weighted mean**	**71**		
**Standard deviation**	**64**		
**n**	**7**		

Katupotha and Fujiwara 1988. (Sri Lanka):
Elevation: all reported referenced to MSL. Outcrop sample; allocated a ± 0.5 m elevation.

No facies formation range for bivalves or gastropods. Corals on SW coast; modern analogues MLWS (i.e., -0.37 m) to -4 in lagoon settings (fossil only differentiated in setting for corals at the Akurala site); MLWS to -8 m. No modern, local analogue for the south coast – used the SW coast coral depth distribution (i.e., -0.37 to -8 m).

Ages have been background corrected but not $\delta^{13}C$ corrected. As these are radiometric measurements (liquid scintillation), used the $^{14}C/^{12}C$ correction worksheet[190] using $\delta^{13}C = 0 \pm 2$ ‰ as these are coral/marine bivalve/gastropod samples. ΔR: see discussion above for the surface circulation. Upwelling in predominately along the southern coast of Sri Lanka (e.g., [255]), which is where the samples are from. Used the weighted mean of 4 samples from Sri Lanka[180] to give a weighted mean of ΔR =133 ± 65 years (n=4). Original authors did not include a ΔR correction (as the ages were not calibrated)

**Location**	**ΔR (years)**	**± 1σ**	**source**
Sri Lanka	101	47	[180]
Sri Lanka	187	53	[180]
Sri Lanka	198	53	[180]
Sri Lanka	63	50	[180]
**Weighted mean**	**133**		
**Standard deviation**	**65**		
**N**	**4**		

SOUTHERN AFRICA

1. SOUTH AFRICA

Ramsey and Cooper, 2002, Grobbler et al., 1988, Maud, unpublished, 1968; Vogel and Visser, 1981; King 1972; Vogel and Marais, 1971; Ramsay, 1991; Reddering, 1988; Yates et al., 1986; Miller et al 1995; Ramsay and Mason, 1990 (various sites)**:**
(Compilation of sea level indicators[85] double checked using the original references[85–95], where available).
Facies formation depth: unclear how these relationships are derived, used the original ones quoted in tables 1 and 2[85]. No elevation uncertainty reported, therefore assigned an arbitrary ± 0.5 m uncertainty.

Age: no ratios reported for the U/Th ages; ^{14}C dates were not calibrated in the original paper[85]; unclear if these have been background or δ^{13}C corrected and some marine samples in the table are reported with a 400-year subtraction already applied. Contacted the authors and the lab for clarification; Pta samples have been both background and δ^{13}C corrected, the δ^{13}C values for the Pta samples were kindly provided by the lab (Stephan Woodborne, *pers. comm.*). For terrestrial samples, we recalibrated the dates using the SHCal13 curve[192]; for marine samples, we applied a post-industrial ΔR value appropriate to location (see discussion below) prior to calibration[196]. Note, all shells and bivalves are assumed to be entirely marine in origin and estuarine oysters which may have a very different ΔR correction (cf. [256]).

Eastern and western coastlines different surface circulation source waters (e.g., [257]), therefore used a 'eastern/Agulhas-type'[180,258] and 'western-Benguela-type'[180,257] ΔR correction prior to calibration of the marine samples.

'Eastern/Agulhas-type'

Location	ΔR (years)	± 1σ	source
Mossel Bay, Western Cape	134	30	[257]
Jeffreys Bay, Eastern Cape	161	30	[257]
Natal	213	57	[180]
**Weighted mean**	**170**		
**Standard deviation**	**84**		
**n**	**9**		

'Western-Benguela-type'

Location	ΔR (years)	± 1σ	source
Lamberts Bay	93	28	[258]
Paternoster	128	19	[258]
Hondeklip	109	28	[258]
Dassen Island	129	20	[258]
Dassan Island	252	21	[258]
Table Bay	106	22	[258]
Table Bay	194	26	[258]
False Bay	219	24	[258]
Cape of Good Hope	224	51	[180]
**Weighted mean**	**157**		
**Standard deviation**	**59**		
**n**	**9**		

2. MOZAMBIQUE

Siesser 1974 (in [97]):
Ages are conventional but the authors have made a correction for "apparent age of seawater" but they do not state what value used. Therefore, unable to convert these to conventional ages, nor to recalibrate the dates (included for reference).

ΔR (included for reference only): no samples in the online database[173] from Mozambique. The surface circulation for region is from the north (e.g., [259]). Used a weighted average of samples in online database[173] from north of the site (Comoros, Madagascar, Seychelles; [180]) and immediately to the south (Natal, [180]) ΔR = 170 ±84 years (n=9).

Location	ΔR (years)	± 1σ	source
Natal	213	57	[180]
Comoros	263	51	[180]
Mayotte, Comoros	119	57	[180]
Diego-Suarez, Madagascar	177	60	[180]
Seychelles	310	59	[180]
Seychelles	147	57	[180]
Seychelles	87	57	[180]
Seychelles	50	57	[180]
Mahe, Seychelles	150	52	[180]
**Weighted mean**	**170**		
**Standard deviation**	**84**		
**n**	**9**		

S.E. Asia

1. SUNDA SHELF

Hanebuth et al., 2000, 2009:
Elevation uncertainty: no uncertainty reported for the core water depth or sampling uncertainty, therefore assigned ± 1 m uncertainty based on the tidal amplitude (tidal amplitude of <2 m[98]) for the former and a ±0.01 cm uncertainty for the latter. The tidal range South China Sea varies from 2.5 to 3.8 m at springs[260]. The sampling uncertainty is likely small, allocated a sampling uncertainty of ± 0.01 m (cf. [183]).

Formation range**:** Used facies formation information of the original authors[98,99]; all samples assigned the same reference water level (RWL) as the mangrove samples, except for designated as bay/lagoon (i.e. marine)[98]. Indicative range of mangroves generally between mean sea level and mean high water (MHW), therefore, RWL = +0.5 ± 0.5 m. In [99], the depth range for the facies is given but not the mid-point, nor the datum to which this refers. Assumed that the datum is mean sea level (MSL), therefore the 2 m range for shore and tidal flat facies become 0 ± 1 m. For the subtidal facies, the depth range is given as 7 m but the formation depth is -2 to -10 m (i.e. range of 8 m). In this instance, used the mid-point -6 ± 4 m.
Summary: we have used

- subtidal facies -6 ± 4 m[99].
- All others used 0 ±1 m

Age: all material dated was obtained from the terrestrial carbon reservoir[98].

2. JAPAN

Tanabe et al, 2009 (Echigo Plain, central Japan, Sea of Japan coast):
We make no correction to the elevation of the samples for the effects of any compaction of the sediments (to ensure database consistency). No facies formation information is provided for any of the samples. All marine bivalve data rejected by the authors (despite two samples of articulated bivalves and therefore likely *in situ*). As such, the data provides a limiting sea level record (e.g., sea level must have been below the terrestrial samples).

The subsidence rate used was calculated from the core itself based on the elevation of marsh sediments deposited prior to land reclamation (i.e. depth ~-16.3 m and an age of ~5.5 ka = -2.96 m/ka)[100]; interseismic subsidence rates (~-3 m/ka) observed between 1987 to 1930[261] and those calculated for the Holocene Shinano River incised valley fills[262] (~-3 m/ka). The original authors assume that "interseismic" subsidence constant throughout the Holocene[100]. The subsidence rate is poorly constrained with no associated uncertainties. We use the rate of -2.7 m/ka given by the authors but allocate a ± 0.5 m vertical uncertainty and a ± 1 ka age uncertainty, which gives a recalculated rate of -2.7 ± 0.18 m/ka.

ΔR: site located on the Sea of Japan side of the island but there are no ΔR determinations for any portion of the Japanese coastline of the Sea of Japan within the online database[173]. Two analyses from the Russian coast of the Sea of Japan (ΔR = 50 ± 30, ΔR = 35 ± 40; both[263]) however, both sites are influenced by different water masses (North current, Russian coast and the Tsushima warm current, Japanese current; [263]). Other analyses in the online database[173]: Southern Korea ΔR = -154 ± 35 years[264]; further north (near Sapporo) ΔR = 94 ± 36 years[265]. Water bathing the site is sourced from the East China Sea[265], therefore used a weighted average for the SE East China Sea (Kikai Island, Kagoshima and Ryukyu Islands); ΔR = -87 ± 67 (n= 12).

Location	ΔR (years)	± 1σ	source
SE coast Kikai Island	Multiple analyses		266
Amani Oshima, Kagoshima	94	30	265
Amani Oshima, Kagoshima	18	30	265
Amani Oshima, Kagoshima	-1	32	265
Okinawa	-126	7	265
Okinawa, Ryukyus	40	31	265
Okinawa, Ryukyus	-6	30	265
**Weighted mean**	**-87**		
**Standard deviation**	**67**		
**n**	**12**		

3. CHINA

Zong, 2004 (and references therein[102–106,108,111–114,116–119]):
Assumed the elevation was determined by levelling, that the samples derive from sediment cores (obtained by something like a hand corer), that the elevations have not been corrected for compaction and that all radiocarbon dates have been background and $\delta^{13}C$ corrected.

For the elevation uncertainties: allocated a ± 0.03 m uncertainty associated with the elevation (i.e., assuming that this was determined by levelling[200,267]); allocated a coring uncertainty of ± 0.05 m (i.e., similar to that for hand coring cf. [200,268]), allocated a ± 0.01 m measurement uncertainty[183,200] and then summed these in quadrature to give the total elevation uncertainty. Note Yellow Sea Datum (YSD) approximates to MSL[269].

Facies formation depth range: Where upper/lower depth of the formation range is not listed (i.e., "local MHW", then used the given "indicative range" to work out the upper and lower bounds i.e., add/subtract half the "indicative range" to the appropriate MHW elevation as listed in Table 2 of tidal parameters). Note, some of the formation ranges are different than the listed indicative range (i.e., in the Appendix of the paper, some of the indicative ranges are narrower than the range using the tidal information listed): we have recalculated all of these using the information regarding the elevations the facies forms (Table 3) and the tidal information listed in Table 2 in the original publication. Contacted the author: "*discrepancies arise due to less stringent levelling etc. and variations in the tidal parameters based on the distance away from the main tide gauge."* (Zong*, pers. comm.*). **Note, the author does not recommend using this dataset as "it needs revision"**.

Where the calculated indicative range derived from the tidal parameters is greater than the published indicative range, we assumed that any discrepancy is due to additional levelling uncertainties (as in the examples) and therefore added the 'extra' uncertainty to the elevation uncertainties.

Where the calculated reference water level (RWL) (i.e., derived from the tidal parameters) is lower in elevation than the published RWL, we have assumed that the published RWL was adjusted for variations in the tidal conditions at that site (i.e. the RWL was increased in elevation). As such we have used the RWL given in the compilation of [101]. Conversely, where the calculated RWL (from tidal parameters) is higher in elevation than the published RWL, we have assumed that this could be due to either differing tidal parameters and/or compaction. However, in the paper, there is no mention of any compaction correction[101]. Again, we have used the published RWL in preference to the recalculated RWL. We were then able to calculate the formation depth range (upper and lower bounds) ensuring that any "correction" to the RWL was incorporated.

Age: Assumed all shells are marine, beach rock, coral and coral sands are all also marine; assumed all dates are conventional radiocarbon dates. ΔR: East China Sea ΔR = 73 ± 17 years[265] (n = 14) using data from Taiwan and Ishigaki Islands; additional data for the Ishigaki Islands[266,270]. The high-resolution data documents fluctuations from positive to negative ΔR from 1900 to 1950[266] suggesting that there are large-scale, short-term fluctuations in ΔR through time[266]. We follow the example of [265] and use the data from Taiwan and Ishigaki Islands for the Yangtze Delta and Fujian and Taiwan marine samples in the compilation of [101].

**Location**	**ΔR (years)**	**± 1σ**	**source**
Danshui, Taiwan	113	37	265
Keelung, Taiwan	54	41	57
Keelung, Taiwan	101	53	57
Keelung, Taiwan	135	42	57
Keelung, Taiwan	71	35	57
Keelung, Taiwan	90	39	57
Suao, Taiwan	-15	59	57
Suao, Taiwan	101	43	57
NE coast Ishigaki, Japan	6	40	266
NE coast Ishigaki, Japan	-136	42	266
NE coast Ishigaki, Japan	-81	61	266
NE coast Ishigaki, Japan	-93	43	266
NE coast Ishigaki, Japan	-103	50	266
NE coast Ishigaki, Japan	52	52	266
NE coast Ishigaki, Japan	62	50	266
NE coast Ishigaki, Japan	-42	47	266
NE coast Ishigaki, Japan	11	46	266
Ishigaki Island, Japan	7	40	270
Ishigaki Island, Japan	-57	40	270
Ishigaki Island, Japan	15	40	270
Ishigaki Island, Japan	104	45	270
Ishigaki Island, Japan	178	45	270
**Weighted mean**	**31**		
**Standard deviation**	**84**		
**n**	**22**		

For the South China Sea there is a seasonal change from anti- to clockwise surface circulation between summer and winter (e.g., [271]). Previous estimate of ΔR = -25 ± 20 years (n=10)[180]; we use an average of all the samples on the perimeter of the South China Sea (including the Danshui and Keelung sites in Taiwan[265]) in the online database[173] (ΔR = 19 ± 18 years, n= 71).

Location	ΔR (years)	± 1σ	source
Xisha (Paracels) Islands	11	40	[180]
Xisha (Paracels) Islands	-10	50	[180]
Xisha (Paracels) Islands	73	60	[180]
Danshui, Taiwan	113	37	[265]
Keelung, Taiwan	54	41	[265]
Keelung, Taiwan	101	53	[265]
Keelung, Taiwan	135	42	[265]
Keelung, Taiwan	71	35	[265]
Keelung, Taiwan	90	39	[265]
Hon Tre Island	Multiple analyses		[272]
Janao Bay, Luzon, Philippines	-57	50	[180]
Mindoro Strait, Philippines	92	70	[180]
Mona Island, Philippines	-37	70	[180]
Ho Chi Minh City	-23	56	[180]
Con Dao Island, Vietnam	-70	30	[273]
**Weighted mean**	**19**		
**Standard deviation**	**18**		
**n**	**71**		

Note: [274] use the dataset of [101] but exclude the Yangtze Delta and Han River Delta datasets on the basis that these may be adversely affected by long-term subsidence associated with sediment loading[275–277] and sediment consolidation[278–280] issues. The Fujian Province dataset is also excluded on the basis that this location is proximal to plate boundaries and may be subject to local tectonic movements.

Lui et al., 2010 (Yangtze River estuary):
Elevation: no coring elevation uncertainty reported. Assume the depth was obtained from ship echo sounding and so used half the average tidal range quoted in [109] (2.7 m average tidal range; 4.7 m maximum tidal range[281], i.e., elevation uncertainty associated with method is ± 1.35 m)

Facies formation: no detailed relationship to sea level at the time of formation is given by the authors.
- DU4: authors use the maximum tidal range in the region (~5m) to suggest RSL between -53 and -62 m. Infer that these sediments were deposited in littoral to tidal-flat environment; used the upper portion of the maximum tidal range (4.7 m;[281]) as the formation depth range (i.e., +2.35 to 0 m referenced to MSL)
- DU3: fining upwards (deepening water depth) and a "nearshore subtidal to nearshore shelf environment inferred by the authors. Unable to determine a facies formation depth for this sample.
- DU2 and DU1: DU2 is "tide affected, nearshore shallow-sea environment", "similar to the present environment with strong tidal influence"; DU1 "deposited in an environment similar to that of DU2" – used the maximum tidal range as the facies formation depth (i.e. 2.35 to -2.35 m referenced to MSL).

Note, samples Beta-240659 and Beta-240660 are storm deposits and therefore they have an unreliable relationship to sea level.

Ages are conventional ^{14}C. ΔR: authors use the value of ΔR = -96 ± 60 from Qingdao (Yellow Sea)[180]. Have assumed all materials are of marine origin except the plant materials, (samples Beta-251941 and Beta-251942) which were calibrated using the IntCal13 curve[196].

Saito et al., 2000 (Yellow River delta)
Chenier (shelly) ridges on the coastal lowland of China (Yellow River delta): formation of these ridges is debated with different mechanisms for different locations globally (differing models invoke sea level, storm surges etc.). Authors[110] explicitly state "the formation of these cheniers is thought to be due to changes in riverine sediment supply by river migration than changes in sea level" (e.g., [282,283]).

Lui and Walker (1989): modern chenier ridges in the same region (Yellow River delta) form close to mean high tide, with shells from the intertidal zone moved landwards to form cheniers[282]. Cheniers built at the front of the supratidal zone (i.e., at the top) by swash due to reduction in sediment supply and a relative increase in wave energy. The formation of a chenier plain is related to variable sediment supply. Storms can cause further landward retreat of the chenier (although they may have been built in the intertidal zone or foreshore mudflats). Potential that

cheniers could be limiting data but the shells are reworked at some time after deposition and therefore the ages may not be the age of formation, rather they are the age of death of the shell (unreliable ages for the feature).

Similarly, the chenier ridges of the region (Yellow River, Bohai Sea) are formed by storm waves[283]. In the modern environment, the base of the chenier is "a little higher than the mean high tide level"[283] but the authors do not define by how much (i.e., imprecise). In addition, the authors state (in relation to the fossil shelly ridges) the "most of these shelly ridges are the product of reworking after abandonment of the Yellow River delta super-lobe[284,285]. All of the shelly ridges in the coastal lowland of eastern China are controlled by sedimentation and reworking, not fluctuations in sea level" [283].

As such, we cannot calculate a formation depth range (due to the unreliable relationship to sea level) but we have included these in the database for reference.

Ages are conventional radiocarbon dates. Only one sample from the area in the online database[173]; ΔR = -178 ± 50 years (Bohai Gulf[180])

Location	ΔR (years)	± 1σ	source
Bohai Gulf	-178	50	[180]
**Weighted mean**	**n/a**		
**Standard deviation**	**n/a**		
**n**	**1**		

Kim and Kennet, 1998 (Yellow Sea and **KOREA**):
Uncertain how the elevation of the sample was determined, assumed the cores were taken from a ship therefore the elevation uncertainty would be half the tidal range. Tidal range Yellow Sea: Korean coast 4 to 8 m; China coast 1 to 2 m[286]; tidal range in the middle of the Yellow Sea is < 2 m[287]. For the elevation uncertainty associated with sediment core taken from a ship, allocated half the tidal range; for central core (CC-02) used ± 1 m; for the core located close to Korea (DH4-1), used ± 3 m.

No facies formation depths for the peat samples dated; can't allocate as there is no modern analogue information.

Age: assumed ages are conventional. Also, assumed the peats are terrestrial and all carbon is from the terrestrial reservoir (although the authors state that the upper peat is a "marine peat").

ΔR: ΔR = -96 ± 60 for the inner Yellow Sea (i.e. Qingdao)[180]; we use the weighted average of the two samples bordering the Yellow Sea[180,264] in the online database[173] as the samples lie within the centre of the basin (anticlockwise surface circulation; see figure 1 in [107]).

Location	ΔR (years)	± 1σ	source
SW coast of Korea	-111	45	[264]
Tsingtao, China	-81	60	[180]
**Weighted mean**	**-100**		
**Standard deviation**	**20**		
**n**	**2**		

Yu et al., 2009 (Leizhou Peninsula):
Elevation of the microatolls are referenced to the highest living coral rather than a tidal datum and therefore we are unable to assign a facies formation range (nor recalculate a relative sea level references to MSL). We include the data here for comparison purposes only.

Ages were determined by TIMS; recalculated ages using decay constants of [225] and assuming a closed system. Note, there may be a typo in the original publication (analysis ID: microatoll 1_FPO-23) - if age is ~6ka (as given in the original publication) then use 0.069 rather than 0.00069 for the $^{230}Th/^{238}U$ ratio.

4. THAILAND

ΔR: seasonally changing surface circulation with the m monsoon within the Gulf of Thailand and the South China Sea surface circulation is also driven by monsoon winds (e.g., [288]). We use the sample from the Gulf of Thailand[180]; ΔR = -19 ± 70 (n=1) as this is the only sample in the online database[173] for the region).

Location	ΔR (years)	± 1σ	source
Ko Ang Trang, Thailand	-19	70	[180]
**Weighted mean**	**n/a**		
**Standard deviation**	**n/a**		

Horton et al., 2005 (compilation[120,122,124,126–128,132,289] and new data):
Note, not all data from the original references are in the database of [121] as these authors reject samples with "missing information" or "uncertainty over the reliability of their relationship to a past sea level". Note we do not include the limiting data in this compilation. Assumed the radiocarbon dates listed are conventional.

Somboon and Thiramongkol, 1992:
Note, some of the sea-level indicators listed in database of [121] have a different sample type in the original publication (listed in shells in [124]); we have used the original sample designation of [124] and assumed that any shells are of marine origin.

Sinsakul, 1992 (review of Thailand relative sea level):
Elevation determined from interpolation of topographic maps but no contour spacing is given, therefore, allocated a ± 0.5 m measurement uncertainty. Again, there is a difference in the sample type for some samples compared to that listed in the database of [121] and we have used the original publication sample designation. Where listed as shell in [123], we have assumed that these are marine in origin. Only included the "verified" data points of [121].

Scoffin and le Tissier, 1998:
The elevations of the fossil corals are given referenced to the height above the highest living reef-front corals, which is limited to low water spring tides[122] (i.e., MLWS, which is given as +1 m[290] referenced to some unspecified datum). The datum is not MSL, as this would, by definition, be above MLWS but the reconstructed elevations in [121] seem to suggest datum is MSL.

Spring Tidal range: 3 m[122]; 3.1 m[290]; we use a 3 m spring tidal range for the location. If MLWS at +1 m referenced to some datum, and the spring tidal range is 3 m, then MSL would be at +2.5 m referenced to the same datum. Therefore, assumed the elevation of the highest living corals is -1.5 referenced to MSL. The lower limit to formation is not given in the publication; assumed to be at ± 0.5 m by [121]. From the zonation of the modern reef close to the site, in the microatoll zone, the corals grow upwards from the reef floor, giving a depth of 15 to 20 cm[290]. As there is no lower limit, we treat these as limiting data (i.e., we have an upper constrain on growth but not a lower constraint)

Assumed the listed radiocarbon dates are the conventional ages, i.e., background and $\delta^{13}C$ corrected.

Chaimanee et al., 1985:
Unable to obtain a copy of the original reference; used the elevation information given in [289] and back calculated the formation depths from the calculated RSL in [121] (as there is no tectonic correction). Note, that the reported conventional age for one of the data points from Satting Pra differs from that reported in [123]; listed as 6300± 140 years[121] but as 6380 ± 140 years[123]). Used the conventional ages listed in [121].

Tiyapunte and Theerarungsikul, 1988:
Unable to obtain a copy of the original reference; used the elevation information given in [289] and back calculated the formation depths from the calculated RSL in [121] (as there is no tectonic correction). Used the conventional ages listed in [121].

5. PENINSULA MALAYSIA

Seasonally reversing surface circulation due to monsoon influences (e.g., [291,292]). For the ΔR, we use a weighted average of the samples from the Vietnam coastline[180,272,273] and Peninsular Malaysia (i.e., Singapore[180]); ΔR = 17 ± 15 years (n=61, note the data of [272] is a time series). Note, previous estimate gives a South China Sea regional average ΔR = -25 ± 20 years[180].

Location	ΔR (years)	± 1σ	source
Singapore	-15	38	180
Singapore	-121	60	180
Con Dao Island, Vietnam	-70	30	273
Ho Chi Minh City	-23	56	180
Hon Te Island	Multiple analyses		272
**Weighted mean**	**17**		
**Standard deviation**	**15**		
**n**	**61**		

Hasan, 2001:
(note this is cited as Kamaludin, 2001 in in Horton et al., 2005 - obtained thesis from Durham University online repository). Used the information from the thesis regarding elevation and age. Assumed ages are conventional.

Geyh et al., 1979:
Used only the 'verified' data points in [121]. Assumed the "relation to mean sea level" is the elevation of the sample dated (as there is no tectonic uplift). These are reported as a depth range; assumed that the mid-point was the mid-point of the sample dated and that the uncertainties stem from the sampling uncertainty only. As such, assigned a ± 0.05 m uncertainty associated with core retrieval (hand auger). No method of determining elevation (e.g., levelling etc.) was stated; assigned a ± 0.5 m uncertainty.

For the formation depth, authors state "mangroves are direct indicators of the tidal zone between mean neaps high and mean springs high"[127], assumed this is MHWN and MHWS but these tidal parameters are not given. Instead, we use the information from the Port Kelang tidal station as given in [128]. MHWN is at +0.81 m referenced to MSL and MHWS is at +2.11 m referenced to MSL. This gives a 1.3 m range, as suggested by the authors[127]. Note, MSL at Port Kelang is 3.627 m above the zero tidal gauge and the MSL value is derived from 13 years of observations (1984 to 1996).

Assumed the ages are conventional. Not calibrated in the original publication; in the database (Data Citation 1), have quoted the calibrated age range of [121] as the reported calibrated age.

Tjia et al., 1983:
only used the oyster data (as the corals are eroded by an unknown amount); assumed these are marine (and that there is no estuarine, i.e., mixing of fresh and marine waters, influence on the ^{14}C ages).

Assumed the ages have been background and $\delta^{13}C$ corrected. Assumed all shells are of marine origin. Not calibrated in the original publication, we include the quoted calibrated age range of [121] as the reported calibrated age.

6. SINGAPORE

ΔR: The sea level studies[130,131] use the study of [180] but quote ΔR = -25 ± 63 years but this is given as -25 ± 20 years in the original radiocarbon paper[180]. The sea level studies[130,131] use this value except for the GEY core where there was a paired bivalve and wood that enabled the authors to determine ΔR = -96 ± 72 years[130,131] but no further information was given. We therefore used a weighted mean of the two Singapore samples[180] in the online database[173]; ΔR = -45 ± 68 years (n=2).

Location	ΔR (years)	± 1σ	source
Singapore	-15	38	180
Singapore	-121	60	180
**Weighted mean**	**-45**		
**Standard deviation**	**68**		
**n**	**2**		

Bird et al., 2007; 2010:
Used the uncorrected elevation[130,131] in order to ensure consistency within the database. Authors correct these elevation for compaction using the method described in [293]; for the GEY core, the authors suggest an 0.5 m lowering due to compaction.

Age: assumed that these are conventional radiocarbon dates. All shells, bivalves and molluscs are assumed to be marine.

Hesp et al., 1998:
Excluded the coral bommies (data from Pulau Semakau) as it is unclear what their relationship to sea level was at the time of formation and they have been eroded (by an unknown amount). As such the elevation given is unreliable. Differences in the sample type between the original publication and that given in [121]; we use the original designations.

Assumed the ages are conventional. Assumed all shells are of marine origin. Not calibrated in the original publication, in the database, we have quoted the calibrated age range of [121].

7. VIETNAM
ΔR: seasonally reversing surface circulation along the Vietnamese coastline (and wider South China Sea) due to the monsoon. We have used a weighted average of the three sites in the online database[173]; ΔR = 18 ±14 years (n=59; note, the data in [272] is a time series).

Location	ΔR (years)	± 1σ	source
Hon Tre Island	Multiple analyses		[272]
Hi Chi Minh City	-23	56	[180]
Con Dao Island, Vietnam	-70	30	[273]
**Weighted mean**	**18**		
**Standard deviation**	**14**		
**n**	**59**		

Michelli, 2008:
Lithification of beachrock occurs within the marine environment, in particular within the phreatic zone with meteoric recrystallization of most elevated beachrocks[133]. The beachrock samples are formed in the intertidal zone whilst the beachridge samples are deposited supratidal zone[133]. However, the beachridge and washover deposits are designated as storm surge deposits and therefore there is no reliable relationship t sea level at the time of formation. Note, the authors use these as sea level maxima as they are deposited above the normal tidal range[133]. However, we cannot determine how far above and so the relationship of these samples to sea level is poorly constrained. Similarly, backshore deposits are deposited above present wave and tides but this relationship is poorly defined.

Ages are conventional. ΔR: authors use a value of ΔR = 190 ± 35 (citing [273] but in the original publication, this is given as an average ΔR of -74 ± 39 years[273]).

Hanebuth et al., 2000, 2009 (Vietnam Shelf): see notes for the Sunda Shelf.

CARIBBEAN

Toscano and Macintyre, 2003 (and references therein[138,144,145,148,156])
Age: none of the radiocarbon dates listed in [139] are $\delta^{13}C$ corrected (and the values quoted in the compilation are the uncorrected values); authors use a correction of $\delta^{13}C$ = 0.0 ‰ for corals samples and -27 ± 0.2 ‰ for peat samples[139] (note, the latter is the value for C3 plants[294]). We have used the ^{14}C ages given in the original publications (note, there are some slight differences to those quoted in [139]), and assumed these have been background but not $\delta^{13}C$ corrected. We apply a $\delta^{13}C$ = -25 ± 2 ‰ correction for terrestrial material and $\delta^{13}C$ = 0 ± 2 ‰ for marine carbonates prior to recalibration.

1. BARBADOS

Abdul et al 2016:
Age: Spike used not stated in the publication, authors confirm that a gravimetric standard was used to calibrate the spike (Nichole Abdul, *pers. comm.*). Ratios have therefore been recalculated to account for gravimetric spike and the decay constants of [225], assuming a closed system. Data referenced to years BP.

Note, there is a typo in the supplementary table of data: sample RGF 8-20-9 the 230/238 ratio is reported as 0.0004 ± 0.0005 but using this value results in an age of ~0.038 ka not the ~ 10.8 ka reported. Nichole Abdul (*pers. comm.*) confirmed value should read 0.1004 ± 0.0005.

Previously published data also reported in the publication (see supplementary table S2).

1. Duplicates of the samples (see e.g., database of [221]):
   RGF 12-15-4 and 4.2; RGF 12-21-7; RGF 12-9-6; RGF 16-12-6; RGF 16-12-7; RGF 7-16-6
   (Note, the age uncertainties are different between [134] and [221]).
2. Samples previously measured by TIMS and reanalysed using ICP-MS[134]
   RGF 12-21-10; RGF 12-21-6; RGF 12-5-2 A and B; RGF 12-9-5; RGF 7-12-2
   These samples are considered as new analyses (note the ratios and the [^{238}U] are very different to those listed in database of [221]).

The authors give a total elevation uncertainty of ± 1 m which includes tidal, measurement and water depth uncertainties[134]. Following other methods in the database, we assign a water depth uncertainty (i.e., half the tidal range = 0.35 m); a coring uncertainty (e.g., ±0.15 m for vibrocoring etc.) and a measurement uncertainty (± 0.01 m). This gives a total elevation uncertainty in the order of ± 0.38 m.

2. JAMAICA

Digerfeldt and Hendry, 1987:
All samples are basal peats. (note that all samples in [138] are included here, not just those in the compilation of [139])
Age: date have not been $\delta^{13}C$ corrected[139] and no details in the original paper[138]. Assumed that these have been background corrected. Recalculated the conventional age using the $^{14}C/^{12}C$ spreasheet[190] using $\delta^{13}C$ = -25 ± 2 ‰, prior to recalibration.

3. BELIZE

Note, potential for differential subsidence and neotectonism given the proximity to plate boundary.
Tidal range: Twin Cays: micro-tidal, range 0.13 m[295]; Carrie Bay: micro-tidal, range 0.15 m[296]

ΔR: we use the time series of ΔR determinations of [297] in the online database[173]; ΔR = -16 ± 31 years

Location	ΔR (years)	± 1σ	source
Glover Reef	Multiple analyses		[297]
**Weighted mean**	**-16**		
**Standard deviation**	**31**		
**n**	**28**		

Macintyre et al., 1995 (Belize, Tabacco Range):
Elevation: not all the samples are basal peat, no compaction correction applied but the authors apply a correction to the elevation for the effects of vibrocoring (but do not provide further details)[144]. We have used the elevation listed in the table of radiocarbon dates.

Age: these have not been $\delta^{13}C$ corrected[139] (no mention in the original paper[144]). Assumed that they have been background corrected and applied a $\delta^{13}C$ = -25 ± ‰ correction prior to recalibration.

No facies formation range quoted in the original publication. Used the following the tidal range as the facies formation range (cf. [139]) i.e., the modern tidal range of 0.66 m, which equates to 0 ± 0.33 m.

Macintyre et al., 2004 (Belize, Twin Cays):
Elevations corrected by the authors for the effects of vibrocoring (using the core recovery and penetration depth) but they do not list the magnitude of the correction[145]. We cannot back-calculate the uncorrected elevation and so we have used the elevations as listed[145].

Age: the radiocarbon dates have not been $\delta^{13}C$ corrected. The ages were calibrated by the authors[145] using the CALIB software and non-standard radiocarbon ages using a $\delta^{13}C$ correction of -27 ± 0.2 ‰ (cf. [294]). We apply a correction of $\delta^{13}C$ = -25 ± 2 ‰ for terrestrial material and of $\delta^{13}C$ = 0 ± 2 ‰ for marine carbonates prior to recalibration. All samples were originally calibrated using the atmospheric curve however, the samples include *Halimeda* sp. sands (*Halimeda* sp. is a calcareous microalgae that obtains carbon from the marine sources). We have assumed that it was the *Halimeda* sp. carbonate flakes that were dated and used the Marine13 curve[196] (with a ΔR = -16 ± 31 years[297]).

No facies formation range quoted in the original publication. Used the facies formation ranges listed in [139] (i.e., the modern tidal range of 0.66 m, which equates to 0 ± 0.33 m).

Gischler and Husdon, 1998 (Glovers Reef, Lighthouse Reef, Turneffe Islands):
Age: not $\delta^{13}C$ nor ΔR corrected[142]; assumed background corrected. Applied a correction of $\delta^{13}C$ = 0 ± 2 ‰ for marine carbonates and $\delta^{13}C$ = -25 ± 2 ‰ for terrestrial organic material prior to recalibration.

Gischler and Husdon, 2004 (Belize Barrier Reef):
Age: samples have been $\delta^{13}C$ corrected[298]; assumed also background corrected. Authors[298] also add 410 years for marine reservoir; we have used the "measured age" as the conventional ^{14}C age and adjust for the 410 year correction applied by the authors.

Gischler and Lomando, 2000 (Glovers Reef, Lighthouse Reef, Turneffe Islands):
Datum not reported[299], assumed this is MSL. Some samples are surface sediment but no further information on the sample type and therefore we cannot determine the relationship to sea level at the time of formation (nor the facies formation depth). No species is given for the corals samples. For the beachrock samples the authors do not list a modern analogue formation depth. We have used the facies formation depth for the samples listed in [188]; indicative meaning (MTL to MLW)/2 and uncertainty is MHW to MTL (i.e. tidal amplitude). Used the tidal information of [296]: M2 tidal component = 5.7 cm amplitude (note, quoted tidal range for Belize of 0.3 m[140]); tidal datum (MLW) = 0 cm; MTL = = +7.5 cm; mean range = 15 cm[296]. Therefore, indicative range referenced to MSL (assuming MTL = MSL) using the formation depth relationship of [188] is 0 to -0.075m and the uncertainty is the MHW to MTL, i.e., ± 0.075 m. As such the final facies formation range is from 0.075 m to -0.15m referenced to MTL (where MTL is assumed to be equivalent to MSL).

Ages are conventional[299]. Authors correct the radiocarbon ages for marine reservoir effects but they do not state what value they use. Recalibrated the ages using the Marine13 calibration dataset[196] and appropriate ΔR.

Shinn et al., 1982 (Carrie Bow Cay):
No depth distributions given for the corals and no formation depth range for the peat samples dated[148]. Used the generic Caribbean mangrove relationship (i.e., formation between HAT and MTL; see [300] and references therein). Unable to determine HAT for the site; used the Twin Cays relationship for the mangrove peats due to the close proximity of the two sites. Note, Carrie Bow Cay is micro-tidal, range 0.15 m[296].

Age: we assume that these have not been $\delta^{13}C$ corrected and use the $^{14}C/^{12}C$ correction spreadsheet[190] and values of $\delta^{13}C$ = -25 ± 2 ‰ and $\delta^{13}C$ = 0 ± 2 ‰ for peat and coral samples respectively prior to recalibration.

Gischler, 2003 (Belize: Glovers Reef and Turneffe Islands):
Age: ages are conventional[140]; authors have used the IntCal98 calibration curve and subtracted a 400 year marine reservoir correction from all samples (including the peat and sediment) except the soil samples. We have used the conventional age and assumed that the peat samples are terrestrial. Unclear what exactly was dated for the carbonate sediment samples; as such we cannot determine a clear relationship between this facies and sea levels. Assumed the shell is marine in origin.

For the peat and shell samples, we have back-calculated reference water level from [300] for Turneffe Islands; for Lighthouse Cay, back-calculated this relationship from the RSL given in [300] and the elevations from [140]. Unable to calculate the relationship for Glovers Reef – discrepancy between the sample types between [140] and [300]; and even assuming all peats (as per [300]) there are differences in the calculated RWL from the same site between these studies. Therefore, we cannot reliably assign a facies formation depth for these samples.

Halley et al., 1977 (Boo Bee patch reef):
Very little information given in the publication, only 1 radiocarbon date on a peat at -18 m (referenced to MSL)[143]. Assumed that this has not been $\delta^{13}C$ corrected but has been background corrected. We apply a correction of $\delta^{13}C$ = -25 ± 2 ‰ prior to recalibration. No facies formation depth is given in the original publication, used the range given in [139].

Wooller et al., 2004, 2007, 2009 (Twin Cays, Turneffe Island):
Elevations given in terms of depth in core but information on the elevation of the dated samples (i.e., no datum given, nor elevation of the core tops)[149–151]. Emailed author for further clarification: "All the cores were taken from sites that were intertidal. i.e. they were within a few cm of the ocean water surface. We were typically stood in some ocean water at the sites. So, these sites were pretty right on the sea level surface" (Matthew Wooller, *pers. comm.*). Used an elevation of 0 ± 0.05 m referenced to MSL for the elevations of the core tops.

No facies formation range quoted in the publications[149–151]. Used the generic mangrove relationship for the Caribbean (i.e., reference water level (RWL) = (MTL+HAT)/2 and Indicative range = HAT to MTL) – see [300] and references therein for the derivation of the modern analogue and facies formation depth. Have back-calculated the RWL and hence able to calculate the upper and lower limits of the formation range using the calculated RSL from [300] and the elevations quoted in the Wooller papers (note, the [300] compilation only give the final recalculated RSL and age rather than the elevation etc.) and the equation RSL = elevation – ref water level to determine the RWL for both sites (Twin Cays an Turneffe Islands).

Age: assumed that the ages are conventional.

Monacci et al., 2009 (Spanish Lookout Cay):
No facies formation range quoted in the publications[147]. Used the generic mangrove relationship for the Caribbean (i.e., reference water level (RWL) = (MTL+HAT)/2 and Indicative range = HAT to MTL) – see [300] and references therein for the derivation of the modern analogue and facies formation depth. We have back-calculated the RWL and hence able to calculate the upper and lower limits of the formation range using the calculated RSL from [300] and the elevations quoted in [147].

Age: assumed that these are conventional.

4. FLORIDA

ΔR: surface waters of the Florida Keys and SE Florida continental reef tract originate in the Gulf of Mexico (see schematic of [301]). Therefore, used the six sites in the online database[173] from the Tortugas[154], Pickles Reef and The Rocks in Florida[302,303]; ΔR = -1 ± 56 years (n=139).

**Location**	**ΔR (years)**	**± 1σ**	**source**
Pickles Reef, Florida	Multiple analyses		303
The Rocks, Florida	Multiple analyses		302
The Rocks, Florida	-3	23	303
The Rocks, Florida	11	24	303
The Rocks, Florida	14	21	303
Tortugas, Florida	114	51	154
**Weighted mean**	**-1**		
**Standard deviation**	**56**		
**n**	**139**		

Robbin, 1984 (Florida Keys):
Unable to obtain the original paper therefore used the information in the compilation of [139]. The facies formation depth range is from [139]which derives from the tidal range. For the Florida sites a tidal range of 0.66 m, i.e., 0 ± 0.33 m[139].

Ages: the radiocarbon ages were not originally $\delta^{13}C$ corrected[139]. We assume that these have been background corrected and apply a correction of $\delta^{13}C$ = -25 ± 2 ‰ for terrestrial organic material prior to recalibration. Note, [139] use a value of -27 ± 0.2 ‰[294] to convert these to conventional ^{14}C ages.

Lighty et al., 1978. (SE Florida shelf):
Assumed samples were obtained from an exposure. The radiocarbon ages were not originally $\delta^{13}C$ corrected[153]. We assume that these have been background corrected and apply a correction of $\delta^{13}C$ = 0 ± 2 ‰ for marine carbonates prior to recalibration. Note, [139]also use a value of 0 ‰ (but no uncertainty quoted) to convert these to conventional ^{14}C ages.

Precht et al., unpublished (in [139]) (upper Florida Keys):
Used the information in compilation of [139]. Ages, used the "14C date" i.e. the measured and uncorrected age. We assume that these samples have been background corrected and we apply a $\delta^{13}C$ correction of $\delta^{13}C$ = 0 ± 2 ‰ for marine carbonates prior to recalibration.

Scholl and Stuiver, 1967 (Everglades):
Elevation uncertainty: no details on how elevation was derived[157], therefore, allocated a ± 0.5 m uncertainty; used the sampling and coring uncertainties as given in the publication plus an additional term (where the authors correct the water depth to depth below MSL at the site). Used the facies formation range in the original publication. Some peat samples do not have a formation depth range, rather the authors give these as limiting indicators (i.e., sea level was below this point). Unable to use the Caribbean generic peat/mangrove relationship (i.e., formation between MTL and HAT e.g., [300]and references therein) as we are unable to obtain tidal HAT tidal datum from NOAA, nor able to back-calculate the reference water level from [300]

Age: not $\delta^{13}C$ corrected[157]. Additional hard-water correction made to the freshwater carbonates (no values given in the publication but in the order of 700 years from a modern sample). Excluded these samples as they do not have a clear relationship to former sea levels, are bulk sediment radiocarbon samples and have an unspecified hard water correction applied to them.

Multer et al., 2002. (Florida Keys):
Recalculated the ages using the decay constants of [225] and assuming a closed system.
Elevations reported in feet[155]; we convert these to metres and the datum is assumed to be MSL. No information on how the elevation was obtained and therefore assigned a ± 0.5 m uncertainty. No facies information or modern assemblage information in the original publication.

Stathakopoulos and Riegl, 2015 (SE Florida continental reef tract):
Insufficient information associated with the U-series analyses to recalculate the age. Note, most but not all the radiocarbon analyses were $\delta^{13}C$ corrected[158]. Assumed all have been background corrected and applied a correction of $\delta^{13}C$ = 0 ± 2 ‰ for marine carbonates, where appropriate, prior to recalibration.

Banks et al., 2007 (SE Florida continental reef tract):
No information on how the elevation was obtained other than via hydraulic drilling[152]. No detailed information on the facies, used the very general descriptions in the text.
Insufficient information associated with the U-series analyses to recalculate the age. Radiocarbon ages are conventional radiocarbon dates.

5. BAHAMAS

ΔR: used a weighted mean of the three ΔR determinations in the online database[173]. ΔR = 25 ± 91 years (n=3).

Location	ΔR (years)	± 1σ	source
Golding Cay, Bahamas	146	66	[154]
Bahama Island	-40	42	[212]
Bahama Island	56	59	[212]
**Weighted mean**	**25**		
**Standard deviation**	**91**		
**n**	**3**		

Lighty et al., 1982 (Abaco barrier reef, Fish and Umbrella Cay):
Assumed exposure/outcrop. Ages in the original publication have not been $\delta^{13}C$ corrected[154]. Assumed they have background corrected. Applied a $\delta^{13}C$ = 0 ± 2 ‰ correction prior to recalibration.

Macintyre et al., 1996. (Stocking Island):
No facies formation range apart from vermetid gastropods on Stocking Island are intertidal[159] (but no tidal range is given). Therefore, we cannot determine the facies formation depth for these samples.

Ages in the original publication have not been $\delta^{13}C$ corrected[159]. Assumed they have background corrected. Applied a $\delta^{13}C$ = 0 ± 2 ‰ correction prior to recalibration.

6. MARTINIQUE

ΔR: no samples in the online database[173] from Martinique. Used a weighted average of samples from Puerto Rico[301,304] and wider Cariaco Basin[305] as the surface circulation for the western Caribbean is dominated by inflow of water masses from the North Equatorial Current and North Brazil Current (e.g., [301]). Note, the Puerto Rico data[304] is a time series. We do not include the Cariaco coral data[306] as there is no uncertainty associated with the ΔR in the online database[173].

Location	ΔR (years)	± 1σ	source
La Parguera, Puerto Rico	Multiple analyses		[304]
Boca de Medio	-31	9	[301]
Isla Tortugas	-22	14	[301]
Cariaco Basin	33	60	[305]
Cariaco Basin	12	50	[305]
**Weighted mean**	**16**		
**Standard deviation**	**40**		
**n**	**45**		

Adey and Burke, 1976; Adey unpublished (in Lighty et al., 1982):
We have used the information within the compilation of [154]. The radiocarbon ages have not been $\delta^{13}C$ corrected[154] but we assume that they have been background corrected. We apply a $\delta^{13}C$ = 0 ± 2 ‰ correction prior to recalibration.

7. PUERTO RICO

ΔR: samples from Kilbourne et al., (2007); weighted mean using all the time series data in the online database[173] gives ΔR = 28 ± 36 years (n=41).

Location	ΔR (years)	± 1σ	source
La Parguera, Puerto Rico	Multiple analyses		[304]
**Weighted mean**	**28**		
**Standard deviation**	**36**		
**n**	**41**		

Macintyre et al., 1982 (in Lighty et al., 1982):
Used the information for the Lighty et al., 1982 compilation. The radiocarbon ages have not been $\delta^{13}C$ corrected but we assume that they have been background corrected. We apply a $\delta^{13}C$ = 0 ± 2 ‰ correction prior to recalibration.

8. PANAMA

ΔR: sea level data[161] is located on the Atlantic side of Panama but there are no ΔR samples for this coastline of Panama in the online ΔR database[173]. We use a weighted average of the data available for the Caribbean Sea; ΔR = 6 ± 20 (n=75). Note, we have excluded the data of [306] as there is no uncertainty associated with the ΔR estimates and; the data of [297,304] are both time series.

Location	ΔR (years)	± 1σ	source
Jamaica	-30	42	212
Jamaica	-44	41	212
Glover Reef, Belize	Multiple analyses		297
Boca de Medio	-31	9	301
Isla Tortgas	-22	14	301
Cariaco Basin	33	60	305
Cariaco Basin	12	50	305
La Parguera, Puerto Rico	Multiple analyses		304
**Weighted mean**	**6**		
**Standard deviation**	**40**		
**n**	**75**		

Macintyre and Glynn, 1976 (in [154]):
Used the information in the compilation of [154]. The radiocarbon ages have not been $\delta^{13}C$ corrected but we assume that they have been background corrected. We apply a $\delta^{13}C$ = 0 ± 2 ‰ correction prior to recalibration.

9. GRAND CAYMAN

Woodroffe, 1981. (Grand Cayman):
Age: assumed that the ages have been both background and $\delta^{13}C$ corrected. The dates were not calibrated in the original publication[164]. Recalibrated using the IntCal13 calibration dataset[196]. Used the facies formation depths in the original publication[164].

10. ANTIGUA

ΔR: no estimates from Antigua in the online database[173]; used the estimates from Puerto Rico and wider Cariaco Basin given the inflow of water masses into the Caribbean Basin (North Equatorial Current and North Brazil Current; see [301])

Location	ΔR (years)	± 1σ	source
La Parguera, Puerto Rico	Multiple analyses		304
Boca de Medio	-31	9	301
Isla Tortugas	-22	14	301
Cariaco Basin	33	60	305
Cariaco Basin	12	50	305
**Weighted mean**	**16**		
**Standard deviation**	**40**		
**n**	**45**		

Macintyre et al., 1985 (included two samples from the compilation of [139]):
Very difficult to read the information relating to elevation and age in the original publication[163]; elevations are listed referenced to the reef crest rather than MSL and so we use the elevations listed in [139]where available. Age: difficult to read the values from the table. No species identification for the corals dated.

Ages in the original publication have not been $\delta^{13}C$ corrected, assumed these have been background corrected. Applied a $\delta^{13}C$ = 0 ± 2 ‰ correction and used the $^{14}C/^{12}C$ correction spreadsheet prior to recalibration.

11. US VIRGIN ISLANDS: ST CROIX

Tectonic setting: the authors have made no correction for tectonic setting as this is currently poorly constrained. However, in the coral database[221] the location is given as subsiding based on the elevation of Last Interglacial corals. We use the rate recalculated in [221].

ΔR: Surface waters of the US Virgin Islands portion of the Caribbean is bathed by the North Equatorial Current (with some influence of the North Brazil Current)[301]. No values in the online database[173] for St Croix. Instead we have assumed that the surface water ΔR would be similar to that off for the surface mixed layer Puerto Rico (La Parguera, Puerto Rico[304]); ΔR = 28 ± 36 years (n=41)

Location	ΔR (years)	± 1σ	source
La Parguera, Puerto Rico	Multiple analyses		[304]
**Weighted mean**	**n/a**		
**Standard deviation**	**n/a**		

Toscano et al., 2011:
Compilation of various sources concentrating on *A. palmata* corals. Authors[307] apply a $\delta^{13}C$ correction to previously published radiocarbon determinations where this was not previously done. ΔR: authors use a value of -5 ± 20 years[308] which derives from a regional (Caribbean) average[309].

Hubbard et al., 2005:
We have assumed that the elevations are referenced to MSL (as reported in [307]) but this is not stated in the original publication[168]. No elevation uncertainty quoted in the original publication; a maximum uncertainty of ± 5 m assigned for some samples (based on the palaeo-water depth of *A. palmata*)[307]. We have assigned a ±0.25 m uncertainty associated with a digital depth gauge[310,311]; a ± 0.15 m uncertainty associated with drilling[200,312] and a ± 0.01 m sampling uncertainty[183,200].

Detailed facies analysis of the fossil coral samples was undertaken by the authors but we cannot link this to specific samples. Instead, used the generic (massive, mixed, branching etc.) facies types given in the core logs[168].

Burke et al 1989:
Very little "raw" information contained in the original reference[167]: elevations, species and radiocarbon dating from [166,307].

Radiocarbon dating undertaken (n = 12) but these are not reported in the original references, rather a subset is given in [166,307]. No $\delta^{13}C$ correction originally applied; (note, [166,307] correct the values given using the CALIB correction sheet assuming that the original measurement would have been $^{14}C/^{12}C$ ratio; in the database we have used the calibrated ages of [166,307] as the reported, calibrated ages). We apply a correction of $\delta^{13}C$ = 0 ± 2 ‰ for marine carbonates prior to recalibration. We are unable to recalculate the U-series dates as there is insufficient information given in the publications (i.e., no raw ratios reported).

Macintyre and Adey, 1990:
No $\delta^{13}C$ correction originally applied to the radiocarbon dates[169]. We assume these have been background corrected and we apply a correction of $\delta^{13}C$ = 0 ± 2 ‰ for marine carbonates prior to recalibration.

Adey, 1975; Adey et al., 1977 and Adey (unpublished):
Supplemented the information in the original publications[165,313] with the information given in [154]. No $\delta^{13}C$ correction applied to the radiocarbon dates in any of the original publications. We assume these have been background corrected and apply a correction of $\delta^{13}C$ = 0 ± 2 ‰ for marine carbonates prior to recalibration.

Macintyre et al., 2008:
Compilation of other data[167–169] (see above) and 4 new data points[166]. No $\delta^{13}C$ correction originally applied. Authors apply a ΔR of -5 ± 20 years derived from a regional (Caribbean) average[309]. We assume these have been background corrected and apply a correction of $\delta^{13}C$ = 0 ± 2 ‰ for marine carbonates prior to recalibration.

12. TRINIDAD

Ramcharan, 2004, Ramcharan and McAndrews, 2006:
Very little detail on the material dated (assumed mangrove peat) or corrections applied to the radiocarbon dates[170,171]. Assumed these have been background and $\delta^{13}C$ corrected. No details on how the elevation of the samples was obtained (we have assumed sample elevations are reported referenced to MSL and allocated a ± 0.5 m elevation uncertainty). No facies formation depth given.

Subsidence rate for Nariva Swamp: submergent coastline with subsidence thought to equal that of the uplift in the NE coastline[314] with a rate of -0.02 to -1.5 m/ka based on an emergent Pleistocene marine terrace at +15 m[315] but the age is poorly constrained; assuming this is Last Interglacial age would give uplift rate of 0.76 ± 0.74 m for the NE coastline (and therefore subsidence of -0.76 ± 0.74 m/ka)

13. BERMUDA

Redfield, 1967 (Long Bay, Somerset; Shelly Bay; Harrington Sound)
Converted elevations in feet to metres. No facies formation depth range given in the original publication[172]; used the generic Caribbean modern analogue (i.e., formation between MTL and HAT; [300] and references therein) using the tidal datum for St George's Island, Bermuda[316].

Age: assumed these have not been $\delta^{13}C$corrected; applied a correction of $\delta^{13}C$ = -25 ± 2 ‰ using the spreadsheet for radiometric analysis[190] (given the age of the samples) prior to recalibration.

REFERENCES:

1. Yokoyama, Y., Lambeck, K., De Deckker, P., Johnston, P. & Fifield, L. K. Timing of the Last Glacial Maximum from observed sea-level minima. *Nature* **406,** 713–6 (2000).
2. Yokoyama, Y., De Deckker, P., Lambeck, K., Johnston, P. & Fifield, L. K. Sea-level at the Last Glacial Maximum: evidence from northwestern Australia to constrain ice volumes for Oxygen Isotope Stage 2. *Palaeogeogr. Palaeoclimatol. Palaeoecol.* **165,** 281–297 (2001).
3. De Deckker, P. & Yokoyama, Y. Micropalaeontological evidence for Late Quaternary sea-level changes in Bonaparte Gulf, Australia. *Glob. Planet. Change* **66,** 85–92 (2009).
4. Nicholas, W. A. *et al.* Pockmark development in the Petrel Sub-basin, Timor Sea, Northern Australia: seabed habitat mapping in support of CO_2 storage assessments. *Cont. Shelf Res.* **83,** 129–142 (2014).
5. Ishiwa, T. *et al.* Reappraisal of sea-level lowstand during the Last Glacial Maximum observed in the Bonaparte Gulf sediments, northwestern Australia. *Quat. Int.* **397,** 373–379 (2016).
6. Jongsma, D. Eustatic sea level changes in the Arafura Sea. *Nature* **228,** 150–151 (1970).
7. Thom, B. G. & Chappell, J. Holocene sea levels relative to Australia. *Search* **6,** 90–93 (1975).
8. Ferland, M. A., Roy, P. S. & Murray-Wallace, C. V. Glacial lowstand deposits on the outer continental shelf of southeastern Australia. *Quat. Res.* **44,** 294–299 (1995).
9. Gill, E. D. Quaternary shorelines research in Australia and New Zealand. *Aust. J. Earth Sci.* **31,** 106–111 (1967).
10. Shepard, M. J. Coastal geomorphology of the Myall Lakes Area, NSW. (University of Sydney, 1970).
11. Switzer, A. D., Sloss, C. R., Jones, B. G. & Bristow, C. S. Geomorphic evidence for mid-late Holocene higher sea level from southeastern Australia. *Quat. Int.* **221,** 13–22 (2010).
12. Thom, B. G., Hails, J. R. & Martin, A. R. H. Radiocarbon evidence against higher postglacial sea levels in eastern Australia. *Mar. Geol.* **7,** 161–168 (1969).
13. Belperio, A. P., Hails, J. R., Gostin, V. A. & Polach, H. A. The stratigraphy of coastal carbonate banks and Holocene sea levels of northern Spencer Gulf, South Australia. *Mar. Geol.* **61,** 297–313 (1984).
14. Belperio, A. P. Land subsidence and sea level rise in the Port Adelaide estuary: Implications for monitoring the greenhouse effect. *Aust. J. Earth Sci.* **40,** 359–368 (1993).
15. Belperio, A. P., Harvey, N. & Bourman, R. P. Spatial and temporal variability in the Holocene sea-level record of the South Australian coastline. *Sediment. Geol.* **150,** 153–169 (2002).
16. Burne, R. V. Relative fall of Holocene sea level and coastal progradation, northeastern Spencer Gulf, South Australia. *BMR J. Aust. Geol. Geophys.* **7,** 35–45 (1982).
17. Harvey, N., Barnett, E. J., Bourman, R. P. & Belperio, A. P. Holocene sea-level change at Port Pirie, South Australia: a contribution to global sea-level rise estimates from tide gauges. *J. Coast. Res.* **1,** 607–615 (1999).
18. Short, A. D., Fotheringham, D. G. & Buckley, R. C. *Coastal morphodynamics and Holocene evolution of the Eyre Peninsula coast, South Australia. Coastal Studies Unit Technical Report 86/2.* (University of Sydney, 1986).
19. Gill, E. D. & Hopley, D. Holocene sea levels in eastern Australia— a discussion. *Mar. Geol.* **12,** 223–233 (1972).
20. Beaman, R., Larcombe, P. & Carter, R. M. New evidence for the Holocene sea-level high from the Inner Shelf, central Great Barrier Reef, Australia. *SEPM J. Sediment. Res.* **Vol. 64A,** 881–885 (1994).
21. Belperio, A. P. Negative evidence for a mid-Holocene high sea level along the coastal plain of the Great Barrier Reef Province. *Mar. Geol.* **32,** 1–9 (1979).
22. Larcombe, P. & Carter, R. M. Sequence architecture during the Holocene transgression: an example from the Great Barrier Reef shelf, Australia. *Sediment. Geol.* **117,** 97–121 (1998).
23. Horton, B. P. *et al.* Reconstructing Holocene sea-level change for the Central Great Barrier Reef (Australia) using subtidal foraminifera. *J. Foraminifer. Res.* **37,** 47–63 (2007).
24. Woodroffe, S. A. Testing models of mid to late Holocene sea-level change, North Queensland, Australia. *Quat. Sci. Rev.* **28,** 2474–2488 (2009).
25. Carter, R. M., Johnson, D. P. & Hooper, K. G. Episodic post-glacial sea-level rise and the sedimentary evolution of a tropical continental embayment (Cleveland Bay, Great Barrier Reef shelf, Australia). *Aust. J. Earth Sci.* **40,** 229–255 (1993).
26. Chappell, J., Chivas, A., Wallensky, E., Polach, H. A. & Aharon, P. Holocene palaeo-environmental changes, central to northern Great Barrier Reef inner zone. *AGSO J. Aust. Geol. Geophys.* **8,** 223–235 (1983).
27. Grindrod, J. & Rhodes, E. G. Holocene sea-level history of a tropical estuary: Missionary Bay, North Queensland. in *Coastal Geomorphology in Australia* (ed. Thom, B. G.) 151–178 (Academic Press, 1984).
28. Harvey, N., Belperio, A. P., Bourman, R. P., James, K. & Brunskill, G. New evidence contributing to the debate on the Holocene high sea-level stand in north east Queensland, Australia. in *Third Joint Conference of the New Zealand Geographical Society and the Institute of Australian Geographers* (eds. Holland, P., Stephenson, F. & Wearing, A.) 177–184 (New Zealand Geographical Society, 2001).
29. Kench, P. S., Smithers, S. G. & McLean, R. F. Rapid reef island formation and stability over an emerging reef flat: Bewick cay, northern Great Barrier Reef, Australia. *Geology* **40,** 347–350 (2012).
30. Larcombe, P., Carter, R. M., Dye, J., Gagan, M. K. & Johnson, D. P. New evidence for episodic post-glacial sea-level rise, central Great Barrier Reef, Australia. *Mar. Geol.* **127,** 1–44 (1995).
31. Leonard, N. D. *et al.* Holocene sea level instability in the southern Great Barrier Reef, Australia: high-precision U–Th dating of fossil microatolls. *Coral Reefs* **35,** 625–639 (2016).

32. Lewis, S. E., Wüst, R. A. J., Webster, J. M. & Shields, G. A. Mid-late Holocene sea-level variability in eastern Australia. *Terra Nov.* **20,** 74–81 (2008).
33. Lewis, S. E. *et al.* Rapid relative sea-level fall along north-eastern Australia between 1200 and 800 cal. yr BP: An appraisal of the oyster evidence. *Mar. Geol.* **370,** 20–30 (2015).
34. Ohlenbusch, R. Post-glacial sequence stratigraphy and sedimentary development of the continental shelf off Townsville, Central Great Barrier Reef Province. (James Cook University, 1991).
35. Spenceley, A. P. The geomorphological and zonational development of mangrove swamps in the Townsville Area, North Queensland. (James Cook University, 1980).
36. Tye, S. A stratigraphic and geochemical study of the Holocene evolution of Southern Halifax Bay, North Queensland. (James Cook University, 1992).
37. Veeh, H. H. & Veevers, J. J. Sea level at -175 m off the Great Barrier Reef 13,600 to 17,000 year ago. *Nature* **226,** 536–537 (1970).
38. Yu, K.-F. & Zhao, J. U-series dates of Great Barrier Reef corals suggest at least +0.7 m sea level ~7000 years ago. *The Holocene* **20,** 161–168 (2010).
39. Gibb, J. G. A New Zealand regional Holocene eustatic sea-level curve and its application to determination of vertical tectonic movements. *R. Soc. New Zeal. Bull.* **24,** 377–395 (1986).
40. Ota, Y. *et al.* Holocene marine terraces in the northeastern coast of North Island, New Zealand. in *International Symposium Coastal Evolution in Holocene* 109–112 (Ministry for Education, Science and Culture, 1983).
41. Ota, Y., Berryman, K. R., Hull, A. G., Miyauchi, T. & Iso, N. Age and height distribution of holocene transgressive deposits in eastern North Island, New Zealand. *Palaeogeogr. Palaeoclimatol. Palaeoecol.* **68,** 135–151 (1988).
42. Brown, L. J. Sheet S76-Kaiapoi. Geological Map of New Zealand l. 63360 (1973).
43. Gibb, J. G. Late Quaternary shoreline movements in New Zealand. (Victoria University of Wellington, 1979).
44. Leach, B. F. & Anderson, A. J. The transformation from an estuarine to lacustrine environment in the lower Wairarapa. *J. R. Soc. New Zeal.* **4,** 267–275 (1974).
45. Leach, B. F. The terminal age for the Lower Wairarapa estuarine environment. *J. R. Soc. New Zeal.* **14,** 207–298 (1984).
46. McFagden, B. G. Age relationship between a Maori plaggen soil and Moa-hunter sites on the west Wellington coast. *New Zeal. J. Geol. Geophys.* **23,** 249–256 (1980).
47. Mildenhall, D. C. Holocene pollen diagrams from Pauatahanui Inlet, Porirua, New Zealand. *New Zeal. J. Geol. Geophys.* **22,** 585–591 (1979).
48. Ota, Y., Hull, A. G. & Berryman, K. R. Coseismic uplift of Holocene marine terraces in the Pakarae River Area, eastern North Island, New Zealand. *Quat. Res.* **35,** 331–346 (1991).
49. Schofield, J. C. Sea level fluctuations during the last 4,000 years as recorded by a chenier plain, Firth of Thames, New Zealand. *New Zeal. J. Geol. Geophys.* **3,** 467–485 (1960).
50. Singh, L. J. Uplift and tilting of the Oterei coast, Wairarapa, New Zealand, during the last ten thousand years. *R. Soc. New Zeal. Bull.* **9,** 217–219 (1971).
51. Suggate, R. P. Post-glacial sea-level rise in the Christchurch metropolitan area, New Zealand. *Geol. En Mijnb.* **47,** 291–297 (1968).
52. Woodroffe, C. D., Curtis, R. J. & McLean, R. F. Development of a chenier plain, Firth of Thames, New Zealand. *Mar. Geol.* **53,** 1–22 (1983).
53. Yoshikawa, T., Ota, Y., Yonekura, N., Okada, A. & Nozomi, I. S. O. Marine terraces and their tectonic deformation on the northeast coast of the North Island, New Zealand. *Geogr. Rev. Jpn.* **53,** 238–262 (1980).
54. Chevalier, J. P. & Salvat, B. Etude géomorphologique de l'atoll fermé de Taiaro. *Cah. du Pacifique* **19,** 169–291 (1976).
55. Delibrias, G., Guillier, M. T. & Labeyrie, J. Gif natural radiocarbon measurements VIII. *Radiocarbon* **16,** 15–94 (1974).
56. Montaggioni, L. F. Makatea Island, Tuamotu Archipelago. in *Proceeding of the Fifth International Coral Reef Congress, Tahiti* (eds. Delesalle, B., Galzin, R. & Salvat, B.) 103–158 (1985).
57. Pirazzoli, P. A. & Montaggioni, L. F. Holocene sea-level changes in French Polynesia. *Palaeogeogr. Palaeoclimatol. Palaeoecol.* **68,** 153–175 (1988).
58. Pirazzoli, P. A. & Montaggioni, L. F. Late Holocene sea-level changes in the northwest Tuamotu Islands, French Polynesia. *Quat. Res.* **25,** 350–368 (1986).
59. Pirazzoli, P. A. Bathymetric mapping of coral reefs and atolls from satellite. in *Proceedings of The Fifth International Coral Reef Congress, Tahiti* (eds. Gabrie, C. & Harmelin, M.) 539–599 (1985).
60. Pirazzoli, P. A. *et al.* Leeward islands, Maupiti, Tupai, Bora Bora, Huahine, Society archipelago. *5th Int. Coral Reef Congr. Tahiti, 27 May -1 June 1985* **1,** 17–72 (1985).
61. Pirazzoli, P. A. & Montaggioni, L. F. Variations recentes du niveau de l'ocean et du bilan hydrologique dans l'atoll de Takapoto (Polynásie Française). *Comptes rendus l'Académie des Sci. Série 2, Mécanique, Phys. Chim. Sci. l'univers, Sci. la Terre* **299,** 321–326 (1984).
62. Pirazzoli, P. A., Delibrias, G., Montaggioni, L. F., Saliege, J. F. & Vergnaud-Grazzini, C. Vitesse de croissance latérale des platiers et évolution morphologique récente de l'atoll de Reao, îles Tuamotu, Polynésie française. *Ann. l'Institut Oçanographique* **63,** 57–68 (1987).
63. Pirazzoli, P. A. & Veeh, H. H. Age ^{230}Th/^{234}U d'une encoche émergéee et vitesses de soulèvement quaternaire à Rurutu, îles Australes. *Comptes Rendus l'Acaḍmie des Sci. Paris* **305,** 919–923 (1987).

64. Pirazzoli, P. A. A reconnaissance and survey of Temoe Atoll (South Pacific Ocean). *J. Coast. Res.* **3,** 307–322 (1987).
65. Pirazzoli, P. A., Montaggioni, L. F., Vergnaud-Grazzini, C. & Saliege, J. F. Late Holocene sea levels and coral reef development in Vahitahi Atoll, eastern Tuamotu Islands, Pacific Ocean. *Mar. Geol.* **76,** 105–116 (1987).
66. Pirazzoli, P. A. & Montaggioni, L. F. Les îles Gambier et l'atoll de Temoe (Polynésie française) : anciennes lignes de rivage et comportement géodynamique. *Géodynamique* **2,** 13–25 (1987).
67. Pirazzoli, P. A., Koba, M., Montaggioni, L. F. & Person, A. Anaa (Tuamotu Islands, Central Pacific): an incipient rising atoll? *Mar. Geol.* **82,** 261–269 (1988).
68. Allen, M. S., Morrison, A. E., Lorrey, A. M., Zhao, J. xin & Jacobsen, G. E. Timing, magnitude and effects of late Holocene sea level drawdown on island habitability, Aitutaki, Cook Islands. *Archaeol. Ocean.* **51,** 108–121 (2016).
69. Goodwin, I. D. & Harvey, N. Subtropical sea-level history from coral microatolls in the Southern Cook Islands, since 300 AD. *Mar. Geol.* **253,** 14–25 (2008).
70. Moriwaki, H., Chikamori, M., Okuno, M. & Nakamura, T. Holocene changes in sea level and coastal environments on Rarotonga, Cook Islands, South Pacific Ocean. *The Holocene* **16,** 839–848 (2006).
71. Yonekura, N. *et al.* Holocene fringing reefs and sea-level change in Mangaia Island, Southern Cook Islands. *Palaeogeogr. Palaeoclimatol. Palaeoecol.* **68,** 177–188 (1988).
72. Guilderson, T. P., Burckle, L., Hemming, S. & Peltier, W. R. Late Pleistocene sea level variations derived from the Argentine Shelf. *Geochemistry Geophys. Geosystems* **1,** (2000).
73. Camoin, G. F. *et al.* Holocene sea level changes and reef growth in southwestern Indian ocean. *Coral Reefs* **16,** 247–259 (1997).
74. Colonna, M., Casanova, J., Dullo, W.-C. & Camoin, G. F. Sea-level changes and δ^{18}O record for the past 34,000 yr from Mayotte reef, Indian Ocean. *Quat. Res.* **46,** 335–339 (1996).
75. Zinke, J. *et al.* Postglacial flooding history of Mayotte Lagoon (Comoro Archipelago, southwest Indian Ocean). *Mar. Geol.* **194,** 181–196 (2003).
76. Montaggioni, L. F. & Faure, G. Response of reef coral communities to sea-level rise : a Holocene model fiom Mauritius (Western Indian Ocean). *Sedimentology* **44,** 1053–1070 (1997).
77. Gischler, E., Hudson, J. H. & Pisera, A. Late Quaternary reef growth and sea level in the Maldives (Indian Ocean). *Mar. Geol.* **250,** 104–113 (2008).
78. Kench, P. S., McLean, R. F. & Nichol, S. L. New model of reef-island evolution: Maldives, Indian Ocean. *Geology* **33,** 145–148 (2005).
79. Kench, P. S., Smithers, S. G., McLean, R. F. & Nichol, S. L. Holocene reef growth in the Maldives: evidence of a mid-Holocene sea-level highstand in the central Indian Ocean. *Geology* **37,** 455–458 (2009).
80. Woodroffe, C. D. Morphology and evolution of reef islands in the Maldives. in *Proceedings of the Seventh International Coral Reef Symposium* (ed. Richmond, R. H.) **2,** 1217–1226 (University of Guam Press, 1993).
81. Woodroffe, S. A. *et al.* Radiocarbon dating of mangrove sediments to constrain Holocene relative sea-level change on Zanzibar in the southwest Indian Ocean. *The Holocene* **25,** 820–831 (2015).
82. Wiedicke, M., Kudrass, H. R. & Hübscher, C. Oolitic beach barriers of the last glacial sea-level lowstand at the outer Bengal shelf. *Mar. Geol.* **157,** 7–18 (1999).
83. Banerjee, P. K. Holocene and Late Pleistocene relative sea level fluctuations along the east coast of India. *Mar. Geol.* **167,** 243–260 (2000).
84. Katupotha, J. & Fujiwara, K. Holocene sea level change on the southwest and south coasts of Sri Lanka. *Palaeogeogr. Palaeoclimatol. Palaeoecol.* **68,** 189–203 (1988).
85. Ramsay, P. J. & Cooper, J. A. G. Late Quaternary sea-level change in South Africa. *Quat. Res.* **57,** 82–90 (2002).
86. Grobbler, N. G., Mason, T. R. & Cooper, J. A. G. uMgababa Lagoon: pre- and post-flood sedimentology. in *Sedimentation in estuaries and lagoons (S.E.A.L.)* (Deptartment of Geology and Applied Geology, University of Natal, 1988).
87. King, L. C. *The Natal Monocline: Explaining the Structure and Origin of Natal.* (University of Natal Press, 1972).
88. Maud, R. R. Quaternary geomorphology and soil formation in coastal Natal. *Zeitschrift ̧fr Geomorphol.* **7,** 155–199 (1968).
89. Miller, D. E., Yates, R. J., Jeradino, A. & Parkington, J. E. Late Holocene coastal change in the southwestern Cape, South Africa. *Quat. Int.* **29/30,** 3–10 (1995).
90. Ramsay, P. J. & Mason, T. R. Development of a type zoning scheme for Zululand coral reefs. *J. Coast. Res.* **6,** 829–852 (1990).
91. Ramsay, P. J. Sedimentology, coral reef zonation, and Late Pleistocene coastline models of the Sodwana Bay continental shelf, northern Zululand. (University of Natal, 1991).
92. Reddering, J. S. V. Evidence for a middle Holocene transgression, Keurbooms estuary, South Africa. *Palaeoecol. Africa* **19,** 79–87 (1988).
93. Vogel, J. C. & Marais, M. Pretoria radiocarbon dates I. *Radiocarbon* **13,** 378–394 (1971).
94. Vogel, J. C. & Visser, E. Pretoria radiocarbon dates II. *Radiocarbon* **23,** 43–80 (1981).
95. Yates, R. J. *et al.* A late mid-Holocene high sea level: A preliminary report of geoarchaeology at Elands Bay, western Cape Province, South Africa. *S. Afr. J. Sci.* **82,** 164–165 (1986).
96. Siesser, W. G. Relict and recent beachrock from southern Africa. *Bull. Geol. Soc. Am.* **85,** 1849–1854 (1974).

97. Ramsay, P. J. 9000 years of sea-level change along the southern African coastline. *Quat. Int.* **31,** 71–75 (1996).
98. Hanebuth, T. J. J., Karl Stattegger & Grootes, P. M. Rapid flooding of the Sunda shelf: a late glacial sea level record. *Science (80-.).* **288,** 1033–1035 (2000).
99. Hanebuth, T. J. J., Stattegger, K. & Bojanowski, A. Termination of the Last Glacial Maximum sea-level lowstand: the Sunda-Shelf data revisited. *Glob. Planet. Change* **66,** 76–84 (2009).
100. Tanabe, S., Tateishi, M. & Shibata, Y. The sea-level record of the last deglacial in the Shinano River incised-valley fill, Echigo Plain, central Japan. *Mar. Geol.* **266,** 223–231 (2009).
101. Zong, Y. Mid-Holocene sea-level highstand along the Southeast Coast of China. *Quat. Int.* **117,** 55–67 (2004).
102. Chen, Y. & Lui, T. Sea-level changes in the last several thousand years, Penghu Islands, Taiwan Strait. *Quat. Res.* **45,** 254–262 (1996).
103. Chen, C., Huang, B. & Wang, M. The chronology of Holocene stratigraphy in Fujian coast. *Taiwan Strait* **1,** 64–73 (1982).
104. Dai, P. Holocene peat formation of Mao-Dian-Liu region, southern Yangtze delta plain. (East China Normal University, Shanghai, 1987).
105. Hong, X. Late Quaternary strata and its palaeogeographic significance of Hang-Jia-Hu plain. in *Geography* 224–231 (East China Normal University Press, 1990).
106. Huang, Z., Zhang, Z. & Zong, Y. Changes of sea level in south China coast since the Late Pleistocene. in *China Sea-level Changes* (ed. The IGCP 200 China Working Group) 178–194 (China Ocean Press, 1986).
107. Kim, J. M. & Kennett, J. P. Paleoenvironmental changes associated with the Holocene marine transgression, Yellow Sea (Hwanghae). *Mar. Micropaleontol.* **34,** 71–89 (1998).
108. Li, P., Qiao, P., Zheng, H., Fang, G. & Huang, G. *Environmental evolution of Zhujiang Delta in the past 10000 years*. (China Ocean Press, 1991).
109. Liu, J. *et al.* Sedimentary record of environmental evolution off the Yangtze River estuary, East China Sea, during the last ~13,000 years, with special reference to the influence of the Yellow River on the Yangtze River delta during the last 600 years. *Quat. Sci. Rev.* **29,** 2424–2438 (2010).
110. Saito, Y. *et al.* Delta progradation and chenier formation in the Huanghe (Yellow River) delta, China. *J. Asian Earth Sci.* **18,** 489–497 (2000).
111. Sun, S. & Huang, Y. *Taihu Lake*. (China Ocean Press, 1993).
112. Wang, Z. The marine transgression of Zhejiang Province, China. in *Quaternary Coastline Changes in China* 143–156 (China Ocean Press, 1991).
113. Yang, D. Tidal-level changes in the Changjiang estuary during Holocene. in *Sea-level Changes* 143–156 (China Ocean Press, 1986).
114. Yim, W. W. S. Radiocarbon dates from Hong Kong and their geological implication. *J. Hong Kong Archaeol. Soc.* **11,** 50–63 (1986).
115. Yu, K. F., Zhao, J. X., Done, T. & Chen, T. G. Microatoll record for large century-scale sea-level fluctuations in the mid-Holocene. *Quat. Res.* **71,** 354–360 (2009).
116. Zhang, Z. & Lui, R. The Holocene stratigraphy of the coast of Hainan Island. *Geogr. Sci.* **7,** 129–138 (1987).
117. Zhao, X. *China Sea-level Change*. (Shandong Scientific and Technological Press, 1996).
118. Zhu, C., Cheng, P., Lu, C. & Wang, W. Shoreline movements in the Yangtze delta and Su-bei coastal areas over the last 7000 years. *Sci. Geogr. Sin.* **16,** 207–213 (1996).
119. Zong, Y. Postglacial stratigraphy and sea-level changes in the Han River Delta, China. *J. Coast. Res.* **8,** 1–8 (1992).
120. Chaimanee, N., Tiyapirach, S. & Jongkanjanasoonthorn, Y. *Quaternary Geology of Satting Pra and Khao Chaison Areas*. (1985).
121. Horton, B. P. *et al.* Holocene sea levels and palaeoenvironments, Malay-Tahi Penninsula, southeaast Asia. *Holocene* **8,** 1199–1213 (2005).
122. Scoffin, T. P. & Le Tissier, M. D. A. Late Holocene sea level and reef-flat progradation, Phuket, South Thailand. *Coral Reefs* **17,** 273–276 (1998).
123. Sinsakul, S. Evidence of Quarternary sea level changes in the coastal areas of Thailand: a review. *J. Southeast Asian Earth Sci.* **7,** 23–37 (1992).
124. Somboon, J. R. P. & Thiramongkol, N. Holocene highstand shoreline of the Chao Phraya delta, Thailand. *J. Southeast Asian Earth Sci.* **7,** 53–60 (1992).
125. Thiramongkol, N. Geomorphology of the lower Central Plain, Thailand. in *Proceedings of the 3rd meeting of the working group of river and coastal plain* 103–117 (Chulalongkorn University, 1984).
126. Tiyapunte, S. & Theerarungsikul, N. *The geology of map sheet Amphoe Nongjik and Changwat Pattani, Geological Survey Report*. (1988).
127. Geyh, M. A., Streif, H. & Kudrass, H.-R. Sea-level changes during the late Pleistocene and Holocene in the Strait of Malacca. *Nature* **278,** 441–443 (1979).
128. Hassan, K. bin. Holocene sea level changes in Kelang and Kuantan , Peninsular Malaysia. (Durham University, 2001).
129. Tjia, H. D., Fujii, S. & Kigoshi, K. Holocene shorelines of Tioman Island in the South China Sea. *Geol. En Mijnb.* **62,** 599–604 (1983).
130. Bird, M. I. *et al.* An inflection in the rate of early mid-Holocene eustatic sea-level rise: a new sea-level curve from Singapore. *Estuar. Coast. Shelf Sci.* **71,** 523–536 (2007).
131. Bird, M. I. *et al.* Punctuated eustatic sea-level rise in the early mid-Holocene. *Geology* **38,** 803–806 (2010).

132. Hesp, P. A., Hung, C. C., Hilton, M., Ming, C. L. & Turner, I. M. A first tentative Holocene sea-level curve for Singapore. *J. Coast. Res.* **14,** 308–314 (1998).
133. Michelli, M. Sea-level changes, coastal evolution and paleoceanography of coastal waters in SE Vietnam since the mid-Holocene. (Christian-Albrechts University, 2008).
134. Abdul, N. A., Mortlock, R. A., Wright, J. D. & Fairbanks, R. G. Younger Dryas sea level and meltwater pulse 1B recorded in Barbados reef crest coral *Acropora palmata*. *Paleoceanography* **31,** 330–344 (2016).
135. Fairbanks, R. G. *et al.* Radiocarbon calibration curve spanning 0 to 50,000 years BP based on paired $^{230}Th/^{234}U/^{238}U$ and ^{14}C dates on pristine corals. *Quat. Sci. Rev.* **24,** 1781–1796 (2005).
136. Peltier, W. R. & Fairbanks, R. G. Global glacial ice volume and Last Glacial Maximum duration from an extended Barbados sea level record. *Quat. Sci. Rev.* **25,** 3322–3337 (2006).
137. Mortlock, R. A., Fairbanks, R. G., Chiu, T. C. & Rubenstone, J. $^{230}Th/^{234}U/^{238}U$ and $^{231}Pa/^{235}U$ ages from a single fossil coral fragment by multi-collector magnetic-sector inductively coupled plasma mass spectrometry. *Geochim. Cosmochim. Acta* **69,** 649–657 (2005).
138. Digerfeldt, G. & Hendry, M. D. An 8000 year Holocene sea-level record from Jamaica: implications for interpretation of Caribbean reef and coastal history. *Coral Reefs* **5,** 165–169 (1987).
139. Toscano, M. A. & Macintyre, I. G. Corrected western Atlantic sea-level curve for the last 11,000 years based on calibrated 14C dates from *Acropora palmata* framework and intertidal mangrove peat. *Coral Reefs* **22,** 257–270 (2003).
140. Gischler, E. Holocene lagoonal development in the isolated carbonate platforms off Belize. *Sediment. Geol.* **159,** 113–132 (2003).
141. Gischler, E. & Lomando, A. J. Holocene cemented beach deposits in Belize. *Sediment. Geol.* **110,** 277–297 (1997).
142. Gischler, E. & Hudson, J. H. Holocene development of three isolated carbonate platforms, Belize, Central America. *Mar. Geol.* **144,** 333–347 (1998).
143. Halley, R. B., Shinn, E. A., Hudson, J. H. & Lidz, B. Recent and relict topography of Boo Bee patch reef, Belize. *Proceedings of the Third International Coral Reef Symposium* **2,** 29–35 (1977).
144. Macintyre, I. G., Littler, M. M. & Littler, D. S. Holocene history of Tabacco Range, Belize, Central America. *Atoll Res. Bull.* **430,** 1–18 (1995).
145. Macintyre, I. G., Toscano, M. A., Lighty, R. G. & Bond, G. B. Holocene history of the mangrove and islands of Twin Cays, Belize, Central America. *Atoll Res. Bull.* **510,** 18 (2004).
146. Toscano, M. A. & Macintyre, I. G. Response to Blanchon P, on Toscano M A and Macintyre I G (2003): Corrected western Atlantic sea-level curve for the last 11,000 years based on calibrated ^{14}C dates from *Acropora palmata* framework and intertidal mangrove peat. *Coral Reefs* **22,** 257–270 (2003).
147. Monacci, N. M., Meier-Grünhagen, U., Finney, B. P., Behling, H. & Wooller, M. J. Mangrove ecosystem changes during the Holocene at Spanish Lookout Cay, Belize. *Palaeogeogr. Palaeoclimatol. Palaeoecol.* **280,** 37–46 (2009).
148. Shinn, E. A. *et al.* Geology and sediment accumulation rates at Carrie Bow Cay, Belize. in *The Atlantic Barrier Reef Ecosystem at Carrie Bow Cay, Belize, I Structure and Communities* (eds. Rützler, K. & Macintyre, I. G.) 63–75 (Smithsonian Institution Press, 1982).
149. Wooller, M. J., Behling, H., Smallwood, B. J. & Fogel, M. Mangrove ecosystem dynamics and elemental cycling at Twin Cays, Belize, during the Holocene. *J. Quat. Sci.* **19,** 703–711 (2004).
150. Wooller, M. J., Morgan, R., Fowell, S., Behling, H. & Fogel, M. A multiproxy peat record of Holocene mangrove palaeoecology from Twin Cays, Belize. *The Holocene* **17,** 1129–1139 (2007).
151. Wooller, M. J., Behling, H., Guerrero, J. L., Jantz, N. & Zweigert, M. E. Late Holocene hydrologic and vegetation changes at Turneffe Atoll, Belize, compared with records from mainland Central America and Mexico. *Palaios* **24,** 650–656 (2009).
152. Banks, K. W., Riegl, B. M., Shinn, E. A., Piller, W. E. & Dodge, R. E. Geomorphology of the Southeast Florida continental reef tract (Miami-Dade, Broward, and Palm Beach Counties, USA). *Coral Reefs* **26,** 617–633 (2007).
153. Lighty, R. G., Macintyre, I. G. & Stuckenrath, R. Submerged early Holocene barrier reef south-east Florida shelf. *Nature* **276,** 59–60 (1978).
154. Lighty, R. G., Macintyre, I. G. & Stuckenrath, R. *Acropora palmata* reef framework: a reliable indicator of sea level in the western Atlantic for the past 10,000 years. *Coral Reefs* 125–130 (1982).
155. Multer, H. G., Gischler, E., Lundberg, J., Simmons, K. R. & Shinn, E. A. Key Largo Limestone revisited: Pleistocene shelf-edge facies, Florida Keys, USA. *Facies* **46,** 229–271 (2002).
156. Robbin, D. M. A new Holocene sea-level curve for the upper Florida Keys and Florida reef tract. in *Environments of south Florida, present and past* (ed. Gleason, P. J.) 437–458 (Miami Geological Society, 1984).
157. Scholl, D. W. & Stuiver, M. Recent submergence of southern Florida: A comparison with adjacent coasts and other eustatic data. *Bull. Geol. Soc. Am.* **78,** 437–454 (1967).
158. Stathakopoulos, A. & Riegl, B. M. Accretion history of mid-Holocene coral reefs from the southeast Florida continental reef tract, USA. *Coral Reefs* **34,** 173–187 (2015).
159. Macintyre, I. G., Reid, R. P. & Steneck, Robert, S. Growth history of stromatolites in a Holocene fringing reef, Stocking Island, Bahamas. *J. Sediment. Res.* **66,** 231–242 (1996).
160. Adey, W. H. & Burke, R. Holocene bioherms (algal ridges and bank-barrier reefs) of the eastern Caribbean. *Bull. Geol. Soc. Am.* **87,** 95–109 (1976).
161. Macintyre, I. G. & Glynn, P. W. Evolution of modern Caribbean fringing reef, Galeta Point, Panama. *Am.*

Assoc. Pet. Geol. Bull. **60,** 1054–1072 (1976).
162. Macintyre, I. G., Raymond, B. & Stuckenrath, R. Recent history of a fringing reef, Bahia Salina del Sur, Vieques Island, Puerto Rico. *Atoll Res. Bull.* **268,** 1–9 (1983).
163. Macintyre, I. G. *et al.* Growth and depositional facies of a windward reef complex (Nonsuch Bay, Antigua, WI). in *Proceeding of the Fifth International Coral Reef Congress, Tahiti vol6* 605–610 (1985).
164. Woodroffe, C. D. Mangrove swamp stratigraphy and Holocene transgression, Grand Cayman Island, West Indies. *Mar. Geol.* **41,** 271–294 (1981).
165. Adey, W. H. The Algal Ridges and Coral Reefs of St. Croix their structure and Holocene development. *Atoll Res. Bull.* **187,** 1–67 (1975).
166. MacIntyre, I. G., Toscano, M. A. & Lundberg, J. Complex environmental patterns and holocene sea level changes controlling reef histories along northeastern St. Croix, USVI. *Atoll Res. Bull.* 1–27 (2008). doi:10.5479/si.00775630.556.1
167. Burke, R. B., Adey, W. H. & Macintyre, I. G. Overview of the Holocene history, architecture and structural components of Tague reef and lagoon. *Terr. Mar. Geol. St. Croix, US Virgin Islands. Spec Publ* 105–109 (1989).
168. Hubbard, D. K. Holocene reef development along the northeastern St. Croix Shelf, Buck Island, U.S. Virgin Islands. *J. Sediment. Res.* **75,** 97–113 (2005).
169. Macintyre, I. G. & Adey, W. H. Buck Island Bar, St Croix, USVI: a reef that cannot catch up with sea level. *Atoll Res. Bull.* **330–338,** (1990).
170. Ramcharan, E. K. Mid-to-late Holocene sea level influence on coastal wetland development in Trinidad. *Quat. Int.* **120,** 145–151 (2004).
171. Ramcharan, E. K. & McAndrews, J. H. Holocene development of coastal wetland at Maracas Bay, Trinidad, West Indies. *J. Coast. Res.* **22,** 581–586 (2006).
172. Redfield, A. C. Postglacial change in sea level in the western North Atlantic Ocean. *Science (80-.).* **157,** 687–692 (1967).
173. Reimer, P. J. & Reimer, R. W. Marine reservoir correction database. (2017).
174. Bowman, G. M. Oceanic reservoir correction for marine radiocarbon dates from northwestern Australia. *Aust. Archaeol.* **20,** 58–67 (1985).
175. Gillespie, R. & Polach, H. A. The suitability of marine shells for radiocarbon dating of Australian prehistory. in *Proceedings of the ninth international conference on radiocarbon dating* (eds. Berger, R. & Suess, H. E.) 404–421 (University of California Press, 1979).
176. Gill, E. D. Australian sea levels in the last 15000 years - Victoria, S.E. Australia. in *Australian sea levels in the last 15,000 years, a review. Monograph Series, Occasional Paper N. 3.* (ed. Hopley, D.) 59–63 (James Cook University, 1983).
177. Ulm, S., Petchey, F. & Ross, A. Marine reservoir corrections for Moreton Bay, Australia. *Archaeol. Ocean.* **44,** 160–166 (2009).
178. Ulm, S. Marine and estuarine reservoir effects in central Queensland, Australia: determination of ΔR values. *Geoarchaeology - An Int. J.* **17,** 319–348 (2002).
179. Druffel, E. R. M. & Griffen, S. Variability of surface ocean radiocarbon and stable isotopes in the southwestern Pacific. *J. Geophys. Res.* **104,** 23607–23613 (1999).
180. Southon, J. R., Kashgarian, M., Fontugne, M., Metivier, B. & Yim, W. W.-S. Marine reservoir corrections for the Indian Ocean and southeast Asia. *Radiocarbon* **44,** 167–180 (2002).
181. O'Connor, S., Ulm, S., Fallon, S. J., Barham, A. & Loch, I. Pre-bomb marine reservoir variability in the Kimberley region, Western Australia. *Radiocarbon* **52,** 1158–1165 (2010).
182. Northern Territory Environment Protection Authority. *Blacktip draft Environmental Impact Statement, Section 7 Existing Marine Environment*. (2005).
183. Shennan, I. Flandrian sea-level changes in the Fenland. II: Tendencies of sea-level movement, altitudinal changes, and local and regional factors. *J. Quat. Sci.* **1,** 155–179 (1986).
184. Lambeck, K., Yokoyama, Y. & Purcell, T. Into and out of the last glacial maximum: Sea-level change during oxygen isotope stages 3 and 2. *Quat. Sci. Rev.* **21,** 343–360 (2002).
185. Shennan, I. & Milne, G. A. Sea-level observations around the Last Glacial Maximum from the Bonaparte Gulf, NW Australia. *Quat. Sci. Rev.* **22,** 1543–1547 (2003).
186. Lambeck, K. & Nakada, M. Late Pleistocene and Holocene sea-level change along the Australian coast. *Palaeogeogr. Palaeoclimatol. Palaeoecol.* **89,** 143–176 (1990).
187. Wilson, G. D. F. *DRAFT Report on Benthic Fauna collected during RV Southern Surveyor Voyage 05-2005 (30 April - 28 May 2005) TAXONOMIC RESULTS*. (2010).
188. Mauz, B., Vacchi, M., Green, A., Hoffmann, G. & Cooper, A. Beachrock: A tool for reconstructing relative sea level in the far-field. *Mar. Geol.* **362,** 1–16 (2015).
189. Jacobs, R. *NSW Ocean and River Entrance Tidal Levels Annual Summary 2014–2015: Report MHL2384*. (2015).
190. Stuiver, M., Reimer, P. J. & Reimer, R. W. $\delta^{13}C$ correction spreadsheet CALIB 7.1. (2017).
191. Short, A. D. *Beaches of the New South Wales coast: a guide to their nature, characteristics, surf and safety.* (Sydney University Press, 2007).
192. Hogg, A. G. *et al.* ShCal13 Southern Hemisphere Calibration, 0–50,000 Years Cal Bp. *Radiocarbon* **55,** 1889–1903 (2012).
193. Harris, R. A. *The tides of Australia. Manual of Tides* (United States Coast and Geodetic Survey, 2012).
194. Maritime Safety Queensland. Semidiurnal Tidal Planes 2016. (2016).

195. Australian Hydrographic Service. Australian National Tide Tables. (2007).
196. Reimer, P. J. *et al.* IntCal13 and Marine13 radiocarbon age calibration curves 0–50,000 years cal BP. *Radiocarbon* **55,** 1869–1887 (2013).
197. Scoffin, T. P., Stoddart, D. R. & Rosen, B. R. The nature and significance of microatolls. *Philos. Trans. R. Soc. London. Ser. B Biol. Sci.* **284,** 99–122 (1978).
198. Smithers, S. G. & Woodroffe, C. D. Microatolls as sea-level indicators on a mid-ocean atoll. *Mar. Geol.* **168,** 61–78 (2000).
199. Murray-Wallace, Colin, V. & Woodroffe, C. D. *Quaternary sea-level changes: a global perspective*. (Cambridge University Press, 2014).
200. Hijma, M. P. *et al.* A protocol for a geological sea-level database. in *Handbook of Sea-Level Research* (eds. Shennan, I., Long, A. J. & Horton, B. P.) 536–553 (John Wiley and Sons Ltd., 2015).
201. Gillespie, R. & Temple, R. B. Radiocarbon dating of shell middens. *Archaeol. Phys. Anthropol. Ocean.* **12,** 26–37 (1977).
202. Permanent Service for Mean Sea. Revised Local Reference (RLR) Diagram for Townsville I. (2017). Available at: http://www.psmsl.org/data/obtaining/rlr.diagrams/637.php. (Accessed: 20th January 2017)
203. Pillans, B. A late Quaternary uplift map for North Island, New Zealand. *R. Soc. New Zeal. Bull.* **24,** 409–417 (1986).
204. Carter, L. & Garlick, R. D. *Ocean Circulation New Zealand*. (National Institute of Water and Atmospheric Research, 1998).
205. Higham, T. F. G. & Hogg, A. G. Radiocarbon dating of prehistoric shell from New Zealand and calculation of the ΔR value using fish otoliths. *Radiocarbon* **37,** 409–416 (1995).
206. Rafter, T. A., Jansen, H. S., Lockerbie, L. & Trotter, M. M. New Zealand reference standards. in *Proceedings of the 8th International Conference on Radiocarbon Dating* H29–H79 (Royal Society of New Zealand, 1972).
207. Sikes, E. L., Samson, C. R., Guilderson, T. P. & Howard, W. R. Old radiocarbon ages in the southwest Pacific Ocean during the last glacial period and deglaciation. *Nature* **405,** 555–559 (2000).
208. McSaveney, M. J. *et al.* Late Holocene uplift of beach ridges at Turakirae Head, south Wellington coast, New Zealand. *New Zeal. J. Geol. Geophys.* **49,** 337–358 (2006).
209. Land Information New Zealand. Standard port tidal levels. *Tide Predictions* (2017). Available at: http://www.linz.govt.nz/sea/tides/tide-predictions/standard-port-tidal-levels. (Accessed: 20th January 2017)
210. Land Information New Zealand. Secondary port tide tables. *Tide Predictions* (2017). doi:http://www.linz.govt.nz/sea/tides/tide-predictions
211. Petchey, F., Anderson, A., Zondervan, A., Ulm, S. & Hogg, A. G. New marine ΔR values for the south Pacific subtropical gyre region. *Radiocarbon* **50,** 373–397 (2008).
212. Broecker, Wallace, S. & Olson, E. A. Lamont radiocarbon measurements VIII. *Radiocarbon* **3,** 176–204 (1961).
213. Pirazzoli, P. A., Montaggioni, L. F., Salvat, B. & Faure, G. Late Holocene sea level indicators from twelve atolls in the central and eastern Tuamotus (Pacific Ocean). *Coral Reefs* **7,** 57–68 (1988).
214. Salvat, B., Richard, G., Poli, G., Chevalier, J. P. & Bagnis, R. Geomorphology and biology of Taiari Atoll, Tuamotu Archipelago. in *Proceedings of the Third International Coral Reef Symposium* (ed. Taylor, D. L.) 289–296 (University of Miami, 1977).
215. Pirazzoli, P. A. Sea-level changes in Rangiroa. in *Proceeding of the Fifth International Coral Reef Congress, Tahiti* (ed. Ricard, M.) 192–198 (1985).
216. Schofield, J. C. Notes on late Quaternary sea levels, Fiji and Rarotonga. *New Zeal. J. Geol. Geophys.* **13,** 199–206 (1970).
217. Dickinson, W. R. Geomorphology and geodynamics of the Cook Austral island seamount chain in the South Pacific Ocean: implications for hotspots and plumes. *Int. Geol. Rev.* **40,** 1039–1075 (1998).
218. Woodroffe, C. D., Short, S. A., Stoddart, D. R., Spencer, T. & Harmon, R. S. Stratigraphy and chronology of late Pleistocene reefs in the Southern Cook Islands, south Pacific. *Quat. Res.* **35,** 246–263 (1991).
219. Kopp, R. E., Simons, F. J., Mitrovica, J. X., Maloof, A. C. & Oppenheimer, M. Probabilistic assessment of sea level during the last interglacial stage. *Nature* **462,** 863–867 (2009).
220. Kopp, R. E., Simons, F. J., Mitrovica, J. X., Maloof, A. C. & Oppenheimer, M. A probabilistic assessment of sea level variations within the last interglacial stage. *Geophys. J. Int.* **193,** 711–716 (2013).
221. Hibbert, F. D. *et al.* Coral indicators of past sea-level change: A global repository of U-series dated benchmarks. *Quat. Sci. Rev.* **145,** 1–56 (2016).
222. Guilderson, T. P. *et al.* Southwest subtropical pacific surface water radiocarbon in a high-resolution coral record. *Radiocarbon* **42,** 249–256 (2000).
223. Stoddart, D. R. Almost-atoll of Aitutaki: geomorphology of reefs and islands. in *Almost-Atoll of Aitutaki: Reef Studies in the Cook Islands, South Pacific* (eds. Stoddart, D. R. & Gibbs, P. E.) 31–58 (Smithsonian Institution Press, 1975).
224. Spencer, T., Stoddart, D. R., Woodroffe, C. D. & Harmon, R. S. Lithospheric flexure and raised reef limestones, S. Cooks-Austral Islands. *Proc. 6th Int. Coral Reef Symp. Aust.* **3,** 485–489 (1988).
225. Cheng, H. *et al.* Improvements in ^{230}Th dating, ^{230}Th and ^{234}U half-life values, and U-Th isotopic measurements by multi-collector inductively coupled plasma mass spectrometry. *Earth Planet. Sci. Lett.* **371–372,** 82–91 (2013).
226. Ludwig, K. R. Isoplot: a geochronological toolkit for Microsoft Excel. (2003).
227. Veeh, H. H. ^{230}Th/^{238}U and ^{234}U/^{238}U ages of Pleistocene high sea level stand. *J. Geophys. Res.* **71,** 3379–

3386 (1966).
228. Gómez, E. A., Borel, C. M., Aguirre, M. L. & Martínez, D. E. Radiocarbon reservoir ages and hardwater effect for the northeastern coastal waters of Argentina. *Radiocarbon* **50,** 119–129 (2008).
229. Richards, H. G. & Craig, J. T. Pleistocene sedimentation and fauna of the Argentine Shelf: II Pleistocene mollusks from the continental shelf off Argentina. *Proc. Acad. Nat. Sci. Philadelphia* **115,** 127–152 (1963).
230. Aguirre, M. L. Palaeobiogeography of the Holocene molluscan fauna from northeastern Buenos Aires Province , Argentina : its relation to coastal evolution and sea level changes. *Palaeogeogr. Palaeoclimatol. Palaeoecol.* **102,** 1–26 (1993).
231. Charó, M. P., Fucks, E. E. & Gordillo, S. Late Pleistocene-Recent marine malacological assemblages of the Colorado River delta (south of Buenos Aires Province): Paleoecology and paleoclimatology. *Quat. Int.* **377,** 52–70 (2015).
232. The Academy of Natural Sciences. Malacolog version 4.1.1: a database of western Atlantic marine mollusca. *Malacolog* (2017). Available at: http://www.malacolog.org/search.php?nameid=11613. (Accessed: 4th February 2017)
233. Norris, R. M. & Grant-Taylor, T. L. Late Quaternary shellbeds, western shelf, New Zealand. *New Zeal. J. Geol. Geophys.* **32,** 343–356 (1989).
234. Woodroffe, C. D. *et al.* Mangrove sedimentation and response to relative sea-level rise. *Annu. Rev. Mar. Sci.* **8,** 243–266 (2016).
235. Tomlinson, P. B. The botany of mangroves. in *The Botany of Mangroves* (Cambridge University Press, 1986).
236. Ellison, A. M. Managing mangroves with benthic biodiversity in mind: moving beyond roving banditry. *J. Sea Res.* **59,** 2–15 (2008).
237. Magori, C. *Tidal analysis and predictions in the Western Indian Ocean. Mombasa*. (Kenya Marine and Fisheries Research Institute, 2008).
238. tide-forecast. Tide Times for Dzaoudzi, Ile de Mayotte. *2017 Tide Times and Forecasts for the World* (2017).
239. Delibrias, G. Carbon-14 in the Southern Ocean. *Radiocarbon* **22,** 684–692 (1980).
240. Krump, L. R. & Hine, A. C. Ooids and sea-level indicators. in *Sea-Level Research: a manual for the collection and evaluation of data* (ed. van de Plassche, O.) 175–194 (Geo Books, 1986).
241. Longman, M. W. Carbonate diagenetic textures from near-surface diagenetic environments. *Am. Assoc. Pet. Geol. Bull.* **64,** 461–487 (1980).
242. Flügel, E. *Microfacies Analysis of Limestone*. (1982).
243. Lambeck, K., Rouby, H., Purcell, A., Sun, Y. & Sambridge, M. Sea level and global ice volumes from the Last Glacial Maximum to the Holocene. *Proc. Natl. Acad. Sci. U. S. A.* **111,** 15296–303 (2014).
244. Murty, T. S. & Henry, R. F. Tides in the Bay of Bengal. *J. Geophys. Res.* **88,** 6069 (1983).
245. Sindhu, B. & Unnikrishnan, A. S. Characteristics of tides in the Bay of Bengal. *Mar. Geod.* **36,** 377–407 (2013).
246. Biwas, S. K. & Agrawal, A. Tectonic evolution of the Bengal Foreland Basin since the Early Pliocene and its implication on the development of the Bengal Fan. in *Recent Geoscientific Studies in the Bay of Bengal and the Andaman Sea. Special Publication No, 29.* 5–19 (Geological Survey of India, 1992).
247. Curray, J. R., Emmel, F. J. & Moore, D. G. The Bengal Fan: morphology, geometry, stratigraphy, history and processes. *Mar. Pet. Geol.* **19,** 1191–1223 (2002).
248. Weber, M. E., Wiedicke, M. H., Kudrass, H. R., Hübscher, C. & Erlenkeuser, H. Active growth of the Bengal Fan during sea-level rise and highstand. *Geology* **25,** 315–318 (1997).
249. Dutta, K., Bhushan, R. & Somayajulu, B. L. K. ΔR correction values for the northern Indian Ocean. *Radiocarbon* **43,** 483–488 (2001).
250. Little, E. A. Radiocarbon age calibration at archeological sites of coastal Massachusetts and vicinity. *J. Archaeol. Sci.* **20,** 457–471 (1993).
251. Brückner, H. Late Quaternary shorelines of India. in *Late Quaternary Sea Level Correlation and Applications* (ed. Scott, B. D.) 169–194 (Kluwer Academic, 1989).
252. Permanent Service for Mean Sea. Revised Local Reference (RLR) Diagram for Tangachchimadam. (2017). Available at: http://www.psmsl.org/data/obtaining/rlr.diagrams/1258.php. (Accessed: 4th January 2017)
253. Permanent Service for Mean Sea. Revised Local Reference (RLR) Diagram for Tuticorin. (2017). Available at: http://www.psmsl.org/data/obtaining/rlr.diagrams/1072.php. (Accessed: 4th February 2017)
254. Permanent Service for Mean Sea. Revised Local Reference (RLR) Diagram for Visakhapatnam. (2017).
255. de Vos, A., Pattiaratchi, C. B. & Wijeratne, E. M. S. Surface circulation and upwelling patterns around Sri Lanka. *Biogeosciences* **11,** 5090 (2014).
256. Reimer, P. J. Marine or estuarine radiocarbon reservoir corrections for mollusks? A case study from a medieval site in the south of England. *J. Archaeol. Sci.* **49,** 142–146 (2014).
257. Wündsch, M. *et al.* The impact of changing reservoir effects on the ^{14}C chronology of a Holocene sediment record from South Africa. *Quat. Geochronol.* **36,** 148–160 (2016).
258. Dewar, G., Reimer, P. J., Sealy, J. & Woodborne, S. Late-Holocene marine radiocarbon reservoir correction (ΔR) for the west coast of South Africa. *The Holocene* **22,** 1481–1489 (2012).
259. Donguy, J.-R. & Piton, B. The Mozambique channel revisited. *Oceanol. Acta* **14,** 549–558 (1991).
260. Woodroffe, C. D. Deltaic and estuarine environments and their Late Quaternary dynamics on the Sunda and Sahul shelves. *J. Asian Earth Sci.* **18,** 393–413 (2000).
261. Commission of Subsidence Investigation in Niigata area. *Subsidence in Niigata*. (1959).

262. Kamoi, Y., Yasui, S. & Kobayashi, I. Reconsideration on alluvium stratigraphy in the central Echigo Plain, northern Japan. *Earth Sci. (Chikyu Kagaku)* **56,** 123–138 (2002).
263. Kuzmin, Y., Burr, G. S. & Jull, A. J. T. Radiocarbon reservoir correction ages in the Peter the Great Gulf, Sea of Japan and eastern ocast of the Kunashir, southern Kuriles (northwestern Pacific). *Radiocarbon* **43,** 477–481 (2001).
264. Kong, G. S. & Lee, C. W. Marine reservoir corrections (ΔR) for southern coastal waters of Korea. *Sea* **10,** 124–128 (2005).
265. Yoneda, M. *et al.* Radiocarbon marine reservoir ages in the western Pacific estimated by pre-bomb molluscan shells. *Nucl. Instruments Methods Phys. Res. Sect. B Beam Interact. with Mater. Atoms* **259,** 432–437 (2007).
266. Hirabayashi, S., Yokoyama, Y., Suzuki, A., Miyairi, Y. & Aze, T. Short-term fluctuations in regional radiocarbon reservoir age recorded in coral skeletons from the Ryukyu Islands in the north-western Pacific. *J. Quat. Sci.* **32,** 1–6 (2017).
267. Törnqvist, T. E. *et al.* Deciphering Holocene sea-level history on the U.S. Gulf Coast: a high-resolution record from the Mississippi Delta. *Bull. Geol. Soc. Am.* **116,** 1026–1039 (2004).
268. Woodroffe, S. A. Holocene relative sea-level changes in Cleveland Bay, north Queensland, Australia. (Durham University, 2006).
269. Hanjiang, W. Modernization of National Geodetic Datum in China. in *Achievements and developments in geographical information management in addressing national, regional and global issues.* 1–6 (United Nations, 2012).
270. Hideshima, S., Matsumoto, E., Abe, O. & Kitagawa, H. Northwest Pacific marine reservoir correction estimated from annually banded coral from Ishigaki Island, southern Japan. *Radiocarbon* **43,** 473–476 (2001).
271. Fang, G., Fang, W., Fang, Y. & Wang, K. A survey of studies on the South China sea upper ocean circulation. *Acta Oceanogr. Taiwanica* **37,** 1–16 (1998).
272. Bolton, A., Goodkin, N. F., Druffel, E. R. M., Griffin, S. & Murty, S. A. Upwelling of Pacific Intermediate Water in the South China Sea revealed by coral radiocarbon record. *Radiocarbon* **58,** 37–53 (2016).
273. Dang, P. X., Mitsuguchi, T., Kitagawa, H., Shibata, Y. & Kobayashi, T. Marine reservoir correction in the south of Vietnam estimated from an annually-banded coral. *Radiocarbon* **46,** 657–660 (2004).
274. Bradley, S. L., Milne, G. A., Horton, B. P. & Zong, Y. Modelling sea level data from China and Malay-Thailand to estimate Holocene ice-volume equivalent sea level change. *Quat. Sci. Rev.* **137,** 54–68 (2016).
275. Ferrier, K. L., Mitrovica, J. X., Giosan, L. & Clift, P. D. Sea-level responses to erosion and deposition of sediment in the Indus River basin and the Arabian Sea. *Earth Planet. Sci. Lett.* **416,** 12–20 (2015).
276. Ivins, E. R., Dokka, R. K. & Blom, R. G. Post-glacial sediment load and subsidence in coastal Louisiana. *Geophys. Res. Lett.* **34,** L16303 (2007).
277. Wolstencroft, M., Shen, Z., Törnqvist, T. E., Milne, G. A. & Kulp, M. Understanding subsidence in the Mississippi Delta region due to sediment, ice, and ocean loading: Insights from geophysical modeling. *J. Geophys. Res. Solid Earth* **119,** 3838–3856 (2014).
278. Brain, M. J. Compaction. in *Handbook of Sea-Level Research* (eds. Shennan, I., Long, A. J. & Horton, B. P.) 452–469 (John Wiley and Sons Ltd., 2015).
279. Horton, B. P. & Shennan, I. Compaction of Holocene strata and the implications for relative sea level change on the east coast of England. *Geology* **37,** 1083–1086 (2009).
280. Teatini, P., Tosi, L. & Strozzi, T. Quantitative evidence that compaction of Holocene sediments drives the present land subsidence of the Po Delta, Italy. *J. Geophys. Res. Solid Earth* **116,** 10 (2011).
281. Chen, J. Y., Shen, H. T. & Yun, C. X. *Dynamic Processes and Morphological Evolution of the Changjiang Estuary*. (Shanghai Science and Technology Press, 1988).
282. Lui, C. & Walker, H. J. Sedimentary characteristics of cheniers and the formation of the chenier plains of east China. *J. Coast. Res.* **5,** 353–368 (1989).
283. Xue, C., Zhu, X. & Lin, H. Holocene sedimentary sequence, foraminifer and ostracoda in west coastal lowland of Bohai Sea, China. *Quat. Sci. Rev.* **14,** 521–530 (1995).
284. Xue, C., Ye, Z. & He, Q. Holocene coastal sedimentation of China. in *Quaternary Geology and Environment in China* 64–72 (Science Press, 1991).
285. Xue, C. Historical change in the Yellow River delta. *Mar. Geol.* **113,** 321–330 (1993).
286. Uda, M. Yellow Sea. in *The Encyclopedia of Oceanography* (ed. Fairbridge, R. W.) 994–998 (Van Nostrand Reinhold, 1966).
287. Teague, W. J., T., P. H., R., H. Z. & Jacobs, G. A. Current and tide observations in the southern Yellow Sea. *J. Geophys. Res.* **103,** 27783–27793 (1998).
288. Chu, P. C., Edmons, N. L. & Fan, C. Dynamical mechanisms for the South China Sea seasonal circulation and thermohaline variabilities. *J. Phys. Oceanogr.* **29,** 2971–2989 (1999).
289. Sinsakul, S. Evidence of Quaternary sea level Changes in the coastal areas of Thailand: a review. *Southeast Asian Earth Sci* **7,** 23–37 (1992).
290. Ditlev, H. Zonation of corals (Scleractinia: Coelenterata) on intertidal reef flats at Ko Phuket, Eastern Indian Ocean. *Mar. Biol.* **47,** 29–39 (1978).
291. Pa'suya, M. F., Omar, K. M., Peter, B. N., Din, A. H. M. & Akhir, M. F. M. Seasonal variation of surface circulation along Peninsular Malaysia's east coast. *J. Teknol.* **71,** 25–33 (2014).
292. Camerlengo, A. & Demmler, M. I. Wind-driven circulation of peninsular Malaysia's eastern continental shelf. *Sci. Mar.* **61,** 203–211 (1997).

293. Bird, M. I., Fifield, L. K., Chua, S. & Goh, B. Calculating sediment compaction for radiocarbon dating of intertidal sediments. *Radiocarbon* **46,** 421–435 (2004).
294. Smith, B. N. & Epstein, S. Two categories of $^{13}C/^{12}C$ ratios for higher plants. *Plant Physiol.* **47,** 377–407 (1971).
295. Urish, D. W., Wright, R. M., Feller, I. C. & Rodriguez, W. Dynamic hydrology of a mangrove island: Twin Cays, Belize. *Smithson. Contrib. Mar. Sci.* **38,** 473–490 (2009).
296. Kjerfve, B., Rützler, K. & Kierspe, G. H. Tides at Carrie Bow Cay, Belize. *Smithson. Contrib. Mar. Sci.* **12,** 47–52 (1982).
297. Druffel, E. R. M. Radiocarbon in annual coral rings of Belize and Florida. *Radiocarbon* **22,** 363–371 (1980).
298. Gischler, E. & Hudson, J. H. Holocene development of the Belize Barrier Reef. *Sediment. Geol.* **164,** 223–236 (2004).
299. Gischler, E. & Lomando, A. J. Isolated carbonate platforms of Belize, Central America: sedimentary facies, late Quaternary history and controlling factors. *Geol. Soc. London, Spec. Publ.* **178,** 135–146 (2000).
300. Khan, N. S. *et al.* Drivers of Holocene sea-level change in the Caribbean. *Quat. Sci. Rev.* **155,** 13–36 (2017).
301. Wagner, A. J., Guilderson, T. P., Slowey, N. C. & Cole, J. E. Pre-bomb surface water radiocarbon of the Gulf of Mexico and Caribbean as recorded in hermatypic corals. *Radiocarbon* **51,** 947–954 (2009).
302. Druffel, E. R. M. & Linick, T. W. Radiocarbon in annual coral rings of Florida. *Geophys. Res. Lett.* **5,** 913–916 (1978).
303. Druffel, E. R. M. Pulses of rapid ventilation in the North Atlantic surface ocean during the past century. *Science (80-.).* **275,** 1454–1457 (1997).
304. Kilbourne, K. H., Quinn, T. M., Guilderson, T. P., Webb, R. S. & Taylor, F. W. Decadal- to interannual-scale source water variations in the Caribbean Sea recorded by Puerto Rican coral radiocarbon. *Clim. Dyn.* **29,** 51–62 (2007).
305. Hughen, K. A., Southon, J. R., Bertrand, C. J., Frantz, B. & Zermeño, P. Cariaco Basin calibration update: revisions to calendar and ^{14}C chronologies for core PL07-58PC. *Radiocarbon* **46,** 1161–1187 (2004).
306. Guilderson, T. P., Cole, J. E. & Southon, J. R. Pre-bomb $\Delta^{14}C$ variability and the Suess effect in Cariaco Basin surface waters as recorded in hermatypic corals. *Radiocarbon* **47,** 57–65 (2005).
307. Toscano, M. A., Peltier, W. R. & Drummond, R. ICE-5G and ICE-6G models of postglacial relative sea-level history applied to the Holocene coral reef record of northeastern St Croix, U.S.V.I.: Investigating the influence of rotational feedback on GIA processes at tropical latitudes. *Quat. Sci. Rev.* **30,** 3032–3042 (2011).
308. Stuiver, M. & Braziunas, T. F. Modeling atmospheric ^{14}C influences and ^{14}C ages of marine samples to to 10,000 BC. *Radiocarbon* **35,** 137–189 (1993).
309. Stuiver, M., Pearson, G. W. & Braziunas, T. F. Radiocarbon age calibration of marine samples back to 9000 cal yr BP. *Radiocarbon* **28,** 980–1021 (1986).
310. Rovere, A. *et al.* Geo-environmental cartography of the Marine Protected Area 'Isola di Bergeggi' (Liguria, NW Mediterranean Sea). *J. Maps* **6,** 505–519 (2010).
311. Azzopardi, E. & Sayer, M. Estimation of depth and temperature in 47 models of diving decompression computer. *Underw. Technol.* **31,** 3–12 (2012).
312. Morton, R. A. & White, W. A. Characteristics of and corrections for core shortening in unconsolidated sediments. *J. Coast. Res.* **13,** 761–769 (1997).
313. Adey, W. H., Macintyre, I. G., Stuckenrath, R. & Dill, R. F. Relict barrier reef system off St Croix: its implications with respect to Late Cenozoic coral reef development in the western Atlantic. *Proceedings, Third International Coral Reef Symposium* **2,** 15–21 (1977).
314. Weber, J. C. Neotectonics in the Trinidad and Tobago, West Indies segment of the Caribbean-South American plate boundary. *Occas. Pap. Geol. Inst. Hungary* **204,** 21–29 (2010).
315. Kugler, H. G. *Geological map and sections of Trinidad. Scale 1:100,000*. (1961).
316. National Oceanic and Atmospheric Administration (NOAA). Datums for 2695540, Bermuda, St. Georges Island. *Tides and Water levels* (2017).
